# The MAP3K-Coding *QUI-GON JINN* (*QGJ*) Gene Is Essential to the Formation of Unreduced Embryo Sacs in *Paspalum*

**DOI:** 10.3389/fpls.2018.01547

**Published:** 2018-10-24

**Authors:** Micaela Mancini, Hugo Permingeat, Carolina Colono, Lorena Siena, Fulvio Pupilli, Celeste Azzaro, Diva Maria de Alencar Dusi, Vera Tavares de Campos Carneiro, Maricel Podio, José Guillermo Seijo, Ana María González, Silvina A. Felitti, Juan Pablo A. Ortiz, Olivier Leblanc, Silvina C. Pessino

**Affiliations:** ^1^Instituto de Investigaciones en Ciencias Agrarias de Rosario, CONICET-UNR, Facultad de Ciencias Agrarias, Universidad Nacional de Rosario, Zavalla, Argentina; ^2^Istituto di Bioscienze e BioRisorse, Consiglio Nazionale delle Ricerche, Perugia, Italy; ^3^Parque Estação Biológica, Embrapa Recursos Genéticos e Biotecnologia, Brasília, Brazil; ^4^Instituto de Botánica del Nordeste, CONICET-UNNE, Corrientes, Argentina; ^5^Facultad de Ciencias Exactas y Naturales y Agrimensura, Universidad Nacional del Nordeste, Corrientes, Argentina; ^6^Facultad de Ciencias Agrarias, Universidad Nacional del Nordeste, Corrientes, Argentina; ^7^DIADE, Univ Montpellier, IRD, Montpellier, France

**Keywords:** apomixis, apospory, *LNC-QGJ*, MAP3K, *Paspalum notatum*, plant reproduction, *QGJ*, *QUI-GON*

## Abstract

Apomixis is a clonal mode of reproduction via seeds, which results from the failure of meiosis and fertilization in the sexual female reproductive pathway. In previous transcriptomic surveys, we identified a mitogen-activated protein kinase kinase kinase (N46) displaying differential representation in florets of sexual and apomictic *Paspalum notatum* genotypes. Here, we retrieved and characterized the N46 full cDNA sequence from sexual and apomictic floral transcriptomes. Phylogenetic analyses showed that N46 was a member of the *YODA* family, which was re-named *QUI-GON JINN* (*QGJ*). Differential expression in florets of sexual and apomictic plants was confirmed by qPCR. *In situ* hybridization experiments revealed expression in the nucellus of aposporous plants’ ovules, which was absent in sexual plants. RNAi inhibition of *QGJ* expression in two apomictic genotypes resulted in significantly reduced rates of aposporous embryo sac formation, with respect to the level detected in wild type aposporous plants and transformation controls. The *QGJ* locus segregated independently of apospory. However, a probe derived from a related long non-coding RNA sequence (*PN_LNC_QGJ*) revealed RFLP bands cosegregating with the *Paspalum* apospory-controlling region (ACR). *PN_LNC_QGJ* is expressed in florets of apomictic plants only. Our results indicate that the activity of *QGJ* in the nucellus of apomictic plants is necessary to form non-reduced embryo sacs and that a long non-coding sequence with regulatory potential is similar to sequences located within the ACR.

## Introduction

Asexual reproduction can naturally occur in ovules of several flowering plant taxa through apomixis, an alternative route to sexuality, which allows the formation of maternal embryos within seeds (Nogler, 1984; Carman, 1997). This atypical trait relies on developmental alterations which cause unreduced cells within the ovule to acquire a reproductive fate. Although mechanistically diverse, apomictic pathways are usually classified into two major classes (i.e., sporophytic and gametophytic), depending on the origin of maternal embryos (Hand and Koltunow, 2014). During sporophytic apomixis, embryogenesis occurs spontaneously in somatic cells of the ovule, leading to the formation of seeds that harbor supernumerary maternal embryos. In contrast, gametophytic apomixis involves the differentiation of functional, unreduced embryo sacs (2n-ES) within the ovule, followed by egg cell parthenogenetic development into embryos (Bicknell and Koltunow, 2004). Depending on the origin of 2n-ESs, gametophytic apomixis can be further subcategorized into: (1) diplospory, when the megaspore mother cell (MMC) fails meiosis and enters into gametogenesis; or (2) apospory, when one or several nucellar or integumental cells, which are usually somatic companions of the MMC, acquire a gametic fate. In all gametophytic apomicts the embryo develops autonomously, while the formation of the endosperm can be either autonomous or fertilization-dependent (pseudogamous) (Bicknell and Koltunow, 2004).

In the last decade, transcriptomic surveys have allowed the identification of hundreds of candidate genes allegedly associated with apomixis in di- and monocotyledonous plants. However, predictions for most of these candidates revealed they belong to a few functional categories, including signal transduction, cell-cycle control, protein turnover, intercellular signaling, transposon activity and transcriptional regulation (Pessino et al., 2001; Rodrigues et al., 2003; Albertini et al., 2004; Cervigni et al., 2008; Laspina et al., 2008; Sharbel et al., 2009, 2010; Yamada-Akiyama et al., 2009; Garcia-Aguilar et al., 2010; Polegri et al., 2010; Okada et al., 2013). Particularly, a transcript fragment (N46) displaying homology with mitogen-activated protein kinase kinase kinases (MAP3K/MAPKKK/MEKK) and similarity to the *Arabidopsis* gene At1g53570 (*MAPKKK3*) was identified in *Paspalum notatum*, an aposporous sub-tropical grass (Laspina et al., 2008). Interestingly, another transcript showing homology with the same *Arabidopsis* gene (A-148-3) mapped to the apospory controlling region (ACR) of *Paspalum simplex*, a single, non-recombinant, dominant superlocus, which confers nearly 100% apospory (Polegri et al., 2010), epigenetically controlled parthenogenesis (Podio et al., 2014a) and the capacity to form endosperm with unbalanced parental genome contributions (Ortiz et al., 2013).

Based on these results and considering the essential roles of MAPKs in plant development (Musielak and Bayer, 2014; Xu and Zhang, 2015), we rationalized that the *P. notatum* At1g53570 homolog N46 might be involved in the switch from sexuality to aposporous apomixis in this species. The central biological question of our work was the following: is the *Paspalum* At1g53570 ortholog (N46) involved in the developmental molecular cascade controlling apospory, either as trigger or participant? To test this hypothesis, we first mined transcriptomic resources available for *P. notatum* to complete the characterization of N46 sequences. We also conducted spatio-temporal expression analyses in sexual and apomictic genotypes of *P. notatum*, and used *Brachiaria brizantha*, a related aposporous species, as a validation control. Finally, we made functional analyses in *P. notatum* by producing RNA interference (RNAi) lines. Moreover, we mapped N46 onto the *P. notatum* genome to explore the occurrence of genetic linkage with apomixis. Finally, we determined that the At1g53570-like transcript previously identified by Polegri et al. (2010) (see above) was not a protein-coding ortholog of At1g53570 and N46, but a lncRNA showing only partial similarity with these genes.

## Materials and Methods

### Plant Material

The *P. notatum* genotypes used in this work belong to the IBONE’s germplasm collection (Instituto de Botánica del Nordeste, UNNE-CONICET, Corrientes, Argentina) and are listed below: (i) natural apomictic tetraploid genotype Q4117 (2*n* = 4*x* = 40) (Ortiz et al., 1997); (ii) experimentally obtained sexual tetraploid genotype Q4188 (2*n* = 4*x* = 40) (Quarin et al., 2003); (iii) colchicine-treated sexual double-diploid genotype C4-4x (2*n* = 4*x* = 40) (Quarin et al., 2001); and (iv) 55 F_1_ hybrids derived from a cross between Q4188 and Q4117, 29 of them sexual and 26 apomictic (Stein et al., 2007). The *Brachiaria brizantha* genotypes used here were: (i) sexual diploid genotype BRA 002747 (B105) (2*n* = 2*x* = 18); and (ii) cultivar Marandu BRA 00591 (B30), a facultative tetraploid apomictic (2*n* = 4*x* = 36). Both genotypes belong to the Embrapa’s germplasm collection and are maintained at Embrapa Genetic Resources and Biotechnology, Brasilia Federal District, Brazil.

### Sequence Analysis

The N46 full-length sequences were retrieved from 454/Roche FLX + floral transcriptome databases generated in prior work (Ortiz et al., 2017) and available at DDBJ/ENA/GenBank under the accessions GFMI00000000 and GFNR00000000, versions GFMI02000000 and GFNR01000000, respectively. Analysis of DNA similarity was done by using the BLASTN and BLASTX packages at the NCBI^1^, the *Arabidopsis* Information Resource^2^ and the Gramene^3^ websites, as well as exploring the Oryza Repeats Database^4^. For open reading frame (ORF) detection, the NCBI ORF Finder tool was used^5^. Gene schemes were constructed with the WormWeb Exon-Intron graphic maker^6^. Alignments and phylogenetic analyses were done with ClustalW2 (Larkin et al., 2007) and MEGA6 (Tamura et al., 2013) software packages, respectively. The evolutionary history was inferred using the UPGMA method (Sneath and Sokal, 1973). Evolutionary distances were computed using the Poisson correction method (Zuckerkandl and Pauling, 1965) (units: number of amino acid substitutions per site). The lncRNA similarity survey was done onto the plant lncRNA GreeNC database (Paytuví Gallart et al., 2016).

### PCR Amplifications

Genomic DNA was extracted from 200 mg of leaves by using CTAB (Paterson et al., 1993). To reveal the presence of the 76-nt intron in the genome, amplification reactions were carried out with a primer pair complementary to the intron flanking regions: FIP upper (5′-ATTTGCAAGGACCAACATCC-3′, *T*_m_: 59.80°C) and FIP lower (5′-ATGGCAAGCAACTTCGATTC-3′, *T*_m_: 60.22°C). To amplify the entire *QGJ* sequence we used the following primers: N46full upper: 5′GCGTGTACGCCTCTCTCTCT3′, *T*_m_: 59.78°C; N46full lower: 5′CTGCATCCTGGGTGAAAAAT3′, *T*_m_: 59.93°C. Reactions (final volume 25 μL) included 1X Real mix qPCR (BIODYNAMICS), 2 mM MgCl_2_, 200 μM dNTPs, 200 nM gene-specific primers and 60 ng genomic DNA. Amplifications were performed in a BIO-RAD thermocycler, programmed as follows: 1 min at 94°C, 35 cycles of 1 min at 94°C, 2 min at 57°C and 2 min at 72°C, and a final elongation step of 5 min at 72°C.

To evaluate the representation of different splice variants by semiquantitative RT-PCR, total RNA was extracted from leaves and/or spikelets at premeiosis/meiosis and reverse transcribed using Superscript II (INVITROGEN). Amplifications were conducted with: (1) the same primer pair flanking the 76-nt intron described above; or (2) a primer pair located inside the intron: IP upper (5′-AAACAGCATGGTGCAGTCAA-3′, *T*_m_: 60.31°C) and IP lower (5′-TCAGGTGGACAATTGATGAGA-3′, *T*_m_: 59.07°C). Each reaction (final volume 25 μL) included the same components used for genomic amplifications and were run in the same thermocycler, but 20 ng of cDNA were used as template, and the cycling was 5 min at 94°C, 25 cycles of 30 s at 94°C, 30 s at 57°C and 45 s at 72°C and a final elongation step of 5 min at 72°C.

To quantitate *QGJ* expression in reproductive organs, the following samples were collected: (1) spikelets of apomictic and sexual *P. notatum* and *B. brizantha* plants (*P. notatum*: Q4117 and C4-4x genotypes; *B. brizantha*: B30 and B105 genotypes) at premeiosis, meiosis and postmeiosis; (2) ovaries of apomictic and sexual *B. brizantha* plants (B30 and B105 genotypes) at the same above-mentioned stages; (3) spikelets at meiosis/young leaves of wild type (Q4117 genotype, apomictic) and transformant (RNAi1, RNAi2, TC1, TC2) *P. notatum* plants. Total RNA was extracted with the SV Total RNA Isolation Kit (PROMEGA), which includes a DNAse treatment step. cDNAs were synthetized with Superscript II (INVITROGEN). All qPCR reactions (final volume: 25 μL) included 200 nM gene-specific primers, 1X Real mix qPCR (BIODYNAMICS) and 20 ng of cDNA. Three biological replicates were processed each into three technical replicates. Replicates with templates produced in the absence of Superscript II (INVITROGEN) and without templates were included (negative controls). Amplifications were performed in a Rotor-Gene Q thermocycler (QIAGEN), programmed as follows: 2 min at 94°C, 45 cycles of 15 s at 94°C, 30 s at 62°C and 40 s at 72°C and a final elongation step of 5 min at 72°C. *QGJ*-specific primers were: (1) N46N upper (5′GGCCCTGCATCTCCTACTTCAT3′, *T*_m_: 68°C) and N46N lower (5′’TGCCCAAACGTCCCACTGC3′, *T*_m_: 62°C), which amplified *QGJ* in all allelic contexts (used for chronological expression analysis); (2) N46S upper (5′AATCGAAGTTGCTTGCCATC3′, *T*_m_: 60°C) and N46S lower (5′GCTCTGTTAGACCGCTGCTT3′, *T*_m_: 59°C), which were located outside the N46 segment cloned into the pBS86-N46 vector (used for analysis of expression in transgenic plants). Non-template reactions were included as controls. β-tubulin was used as an internal reference gene, as recommended by Felitti et al. (2011), Ochogavía et al. (2011), and Podio et al. (2014b), who worked in the same plant model. Relative quantitative expression levels were calculated by using REST-RG (Relative Expression Software Tool V 2.0.7 for Rotor Gene, Corbett Life Sciences) considering take-off and amplification efficiency values for each particular reaction.

### *In situ* Hybridization (ISH) Analyses

Spikelets of *P. notatum* (genotypes Q4117 and C4-4x) and *B. brizantha* (genotypes B30 and B105) were collected at premeiosis/meiosis. Flowers were dissected, fixed in 4% paraformaldehyde/0.25% glutaraldehyde/0.01 M phosphate buffer pH 7.2, dehydrated in an ethanol series and embedded in paraffin (for *P. notatum*) or butyl-methyl-methacrylate (BMM) (for *B. brizantha*). Specimens were cut into sections of 10 μm (*Paspalum*) or 3.5 μm (*Brachiaria*) and placed onto slides treated with poly-L-lysine 100 μg/mL. The paraffin or BMM were removed with xylene or acetone series, respectively. Prior to hybridization, control sections were stained with acridine orange and examined under UV light to verify RNA integrity. A plasmid including the original N46 fragment isolated by Laspina et al. (2008) was linearized using restriction enzymes *Nco*I or *Sal*I (Promega). Sense and anti-sense probes were labeled with the Roche Dig RNA Labeling kit (SP6/T7), following the manufacturers’ instructions, and hydrolyzed to 150–200 bp fragments. Prehybridization was carried out in 0.05 M Tris–HCl pH 7.5 buffer containing 1 μg/mL proteinase K in a humid chamber at 37°C for 10 min. Hybridization was carried out overnight in a humid chamber at 42°C, in 10 mM Tris–HCl pH 7.5 buffer containing 300 mM NaCl, 50% formamide (deionized), 1 mM EDTA pH 8, 1 X Denhardt’s solution, 10% dextran sulfate, 600 ng/mL tRNA and 600 ng/mL of probe. Detection was performed following the instructions of the Roche Dig Detection kit, using anti DIG AP and NBT/BCIP as substrates. Sections were mounted in glycerol 50% and observed under Leica DMRX (*Paspalum* experiments) or Zeiss-Axiophot (*Brachiaria* experiments) light microscopes.

### Plant Transformation

A vector containing an N46 hairpin (pBS86-N46) (Pact1D:rfa-n46-s:rga2i:rfa-n46-as:T35s/Pubi:bar:Tnos) was constructed from cloning the complete N46 fragment (451 bp) reported by Laspina et al. (2008) into the selector, bar-containing plasmid pBS86 (Thompson et al., 1987), which includes two insertion sites in opposite orientation (cgf-s and cgf-as). The rice act1 promoter was considered suitable, because it drives expression in male and female reproductive tissues in rice (Zhang et al., 1991) and *P. notatum* (Mancini et al., 2014). Briefly, attB1 and attB2 Gateway sequences were included in the 5′ and 3′ ends of N46-specific PCR primers (Forward primer: GGGGACAAGTTTGTACAAAAAA GCAGGCTTCCCCTCCTCCCCTGTGCCGAC; Reverse primer: GGGGAC CACTTTGTACAAGAAAGCTGGGTTAAGCCTCCCCAAACGGACCAT). An amplicon was generated from a pGemTeasy N46 clone (Laspina et al., 2008), purified by using a Qiagen column and mixed with a Gateway donor vector and BP Clonase enzymes. The recombination mix was used to transform DH5α competent cells (INVITROGEN). The entry clone was then transferred into the Gateway Destination vector pBS86 using LR clonase. The insertion was validated by sequencing at the Plant Biotechnology Centre, Melbourne, VIC, Australia. The pBS86-*N46* vector, together with the reporter plasmid pact1-gfbsd2 (Ochiai-Fukuda et al., 2006) carrying the *eGFP* gene (encoding an enhanced green fluorescent protein) were used to co-transform *P. notatum* plants (Q4117 genotype) with a protocol previously developed in our laboratory (Mancini et al., 2014). Transformation events were identified by PCR amplification of the transgenes from genomic DNA using the following primers: eGFPF 5′GGGGACAGCTTTCTTGTACAAAGTGGGGATGGTGAGCAAGGGCGAGGAGCT3′ (*T*_m_: 65.4°C)/eGFPR 5′-GGGGACAACTTTGTATAAAGTTGGTTACTTGTACAGCTCGTCCATGCC-3′ (*T*_m_: 66.1°C) (used to detect *eGFP* within pact1-gfbsd2) and BARXLF 5′-CCGGCGGTCTGCACCATCGT-3′ (*T*_m_: 66°C)/BARXR 5′-ATCTCGGTGACGGGCAGGAC-3′ (*T*_m_: 66°C) (used to detect *BAR* within pBS86-N46). Reactions of 25 μl final volume included 1x Taq polymerase buffer (PROMEGA), 0.2 mM forward and reverse primers, 2 mM MgCl_2_, 0.2 μM dNTPs, 50 ng genomic DNA and 1U Taq Polymerase (PROMEGA). Positive (with 20 ng of pact1-gfbsd2 or pBS86-N46) and negative (non-template) controls were run in parallel. Cycling consisted of 5 min at 94°C, 35 cycles of 30 s at 94°C, 1 min at the annealing temperature (*T*_a_) and 30 s at 72°C, and a final 10 min extension at 72°C. The *T*_a_ was set at 2°C less than the lower predicted *T*_m_. Calli transient transformation and eGFP Pact1-directed expression in reproductive tissues was followed by using an Eclipse E200 fluorescence microscopy (Nikon, Tokyo, Japan) with an standard filter cube for excitation 470/40 nm; emission 535/50 nm. Transgenic plants were grown in controlled chambers at IICAR, CONICET-Facultad de Ciencias Agrarias, Universidad Nacional de Rosario, Argentina, under a 14 h photoperiod (150–200 μE.m^-2^.s^-1^) at 26 ± 2°C.

### Cytoembryological Observations and Pollen Viability Tests

Spikelets at anthesis were fixed in FAA (70% ethanol:formaldehyde:acetic acid 18:1:1) for 24–48 h. Ovaries were dissected and placed in 70% ethanol for at least 24 h, treated with 3% H_2_O_2_ during 2 h and dehydrated in an ethanol series (50%, 70%, 95% and twice 100%; 30 min each step). Next, they were cleared using a series of methyl salicylate/ethanol (v:v) solutions (1:1, 3:1, 5.6:1; 30 min for each step). Finally, ovaries were incubated in methyl salicylate for at least 12 h and examined using a Leica DM2500 microscope equipped with DIC optics. Pollen viability was estimated by staining with Alexander’s reagent (Alexander, 1980). Purple-stained grains were considered to be viable whereas lack of staining (i.e., pale-green/non-colored grains) indicated sterility. Observations were carried out in a Nikon Eclipse E200 microscope.

### Statistical Methods

The average number of aposporous and meiotic embryo sacs per ovule was compared among four independent transgenic events and the control genotype Q4117. A modified Shapiro–Wilk test was used to test the normal distribution of the variables (Shapiro and Wilk, 1965). Due to the non-normal distribution detected, the variables were compared using the non-parametric tests of Wilcoxon (Wilcoxon, 1945) and Kruskal–Wallis (Kruskal and Wallis, 1952). Confidence intervals for observed proportions were calculated following the method described by Newcombe (1998), derived from a procedure outlined by Wilson (1927) with a correction for continuity^7^. Chi^2^ tests for homogeneity were calculated with the R software^8^.

### Linkage Analyses

An F_1_ population of 55 individuals, derived from a cross between sexual Q4188 as pistillate parent and apomictic Q4117 as male progenitor and characterized for reproductive modes (29 sex: 26 apo) (Stein et al., 2007) was used for linkage analyses. For N46 bulked segregant analysis (BSA), 30 μg of genomic DNA from the two parental lines and two equitable bulks of 10 sexual and 10 apomictic F_1_ hybrid progenies were digested with *EcoR*I, *Hind*III, and *Pst*I. Samples were loaded in 1% agarose gel (1xTAE), electrophoresed at 40 mA and blotted onto nylon membranes (Hybond N, Amersham) using 10x SSC buffer. DNA was fixed at 80°C for 2 h. DIG probe labeling (the same N46 fragment used for ISH analyses), hybridization and detection were performed as described by Ortiz et al. (1997). For A-148-3 linkage analysis, 5 μg of genomic DNA from the two parental lines and the 55 F_1_s were digested using *Eco*RI, electrophoresed and finally blotted onto Nylon membranes. A-148-3 was converted into an RFLP probe according to Polegri et al. (2010). Probe ^32^P labeling, blot hybridization and exposition to X-ray films was performed according to Pupilli et al. (2001).

### LncRNA Expression Analysis

PCR amplifications were conducted from 50 ng of cDNA produced from total RNA extracted from leaves or flowers, with upper primer LNCU: 5′-AATTGTGCGAAATCCAATCA-3′ and lower primer LNCL: 5′-TTCACCATTACTGCCCACAA-3′. The cycling program included 1 cycle of 1 min at 94°C, 30 cycles of 1 min at 94°C, 2 min at 57°C and 2 min at 72°C and a final elongation cycle of 5 min at 72°C.

## Results

### Characterization of N46 Full-Length Sequence

**FIGURE 1:**
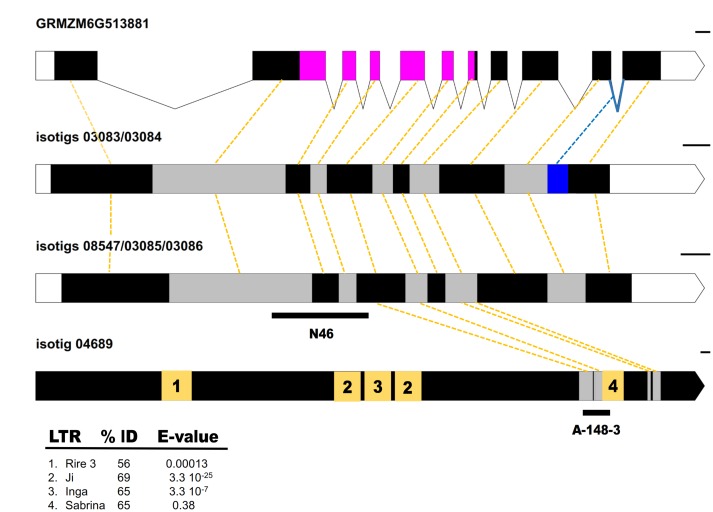
Structural and functional annotations of *Paspalum QUI-GON JINN* and *PN_LNC_QGJ* sequences. **(A)** Annotated genomic sequence of the *QGJ* maize ortholog GRMZM6G513881. White and black boxes denote UTRs and exons, respectively. Lines denote introns. The STK domain is indicated in magenta. The intron involved in the *Paspalum* splice variants in indicated in blue. **(B)** Transcript splice variant corresponding to isotigs 03083/03084, with the last intron represented in the sequence (blue). Gray and black boxes denote different subsequent exons. White boxes denote 5′ and 3′ UTRs. **(C)** Transcript splice variant corresponding to isotigs 08547/03085/03086. The position of the original N46 fragment (Laspina et al., 2008) was indicated below. Gray and black boxes denote different subsequent exons. White boxes denote 5′ and 3′ UTRs. **(D)** *PN_LNC_QGJ* long non-coding RNA. Sequences similar to repetitive elements were numbered on the yellow boxes according to **(E)** and unknown sequences are indicated in black. Sequences similar to *QGJ* are indicated in gray. The position of the original A-148-3 fragment (Polegri et al., 2010) was indicated below. **(E)** Summary table for *PN_LNC_QGJ* BLAST analysis (Gramineae Repeats Database). Yellow dashed lines join corresponding exons. Bars at the right indicate the drawing scale: 100 bases.

Laspina et al. (2008) reported similarity between a mRNA fragment differentially expressed in florets of sexual and apomictic *P. notatum* plants (N46) and a full-length cDNA transcribed from the maize gene GRMZM6G513881 (see footnote 3; NCBI Reference Sequence NM_001137220.1), which encodes a MAP3K protein. Here, we took advantage of *Paspalum* 454/Roche FLX + floral transcriptomes recently developed in our laboratory to recover the N46 full cDNA sequences from apomictic (Q4117) and sexual (C4-4x) genotypes and carry out molecular phylogenetic analysis. In the apomictic floral transcriptome library, we detected one isogroup (apoisogroup 00379) represented by four homologous isotigs, namely apoisotig 03083 (GFMI02003139.1), apoisotig 03084 (GFMI02003140.1), apoisotig 03085 (GFMI02003141.1) and apoisotig 03086 (GFMI02003142.1). In the sexual floral transcriptome library, we also found one isogroup (sexisogroup 02509), but it contained a single isotig (sexisotig 08547; GFNR01008571.1). ClustalW nucleotide (nt) sequence alignments revealed that apoisotigs 03085 and 03086 and sexisotig 08547 were highly similar, differing only by a few polymorphisms (SNPs and INDELs) (Supplementary Figure S1). Apoisotigs 03083 and 03084, were respectively, identical to apoisotigs 03085 and 03086, except for a 76-nt insertion with the canonical donor-receptor sites of GU-AG-type introns, which corresponds to a partially conserved intron in maize GRMZM6G513881. These results suggest that *P. notatum* N46-like floral sequences are genetic and splice variants of a single locus with at least two different alleles in the apomictic genotype (apoisotigs 03085/03083 and 03086/03084) and a third allele detected in the sexual genotype (sexisotig 08547). The intron-like insertion (located between positions 1857–1934 and 1833–1908 in apoisotigs 03083 and 03084, respectively) modifies the reading frame to produce a protein with a variable C-terminal end (Figure 1 and Supplementary Figure S2). Genomic amplification with flanking primers showed that the intron-like region is present in both apomictic and sexual plants (Supplementary Figure S3). Although the non-processed form had been sequenced only from the apomictic samples in the 454/Roche FLX + transcriptome libraries, semi-quantitative RT-PCR experiments showed that both variants (processed and non-processed) were represented in flowers of apomictic and sexual plants (Supplementary Figure S3). Moreover, qPCR of cDNAs originated from a mix of flowers at different developmental stages (from premeiosis to anthesis) with primers located inside the intron revealed no significant differential representation between reproductive modes (not shown). BLASTX searches using N46 full-length sequences as queries identified homology to *MAP3K* genes belonging to the *YODA* family (best annotated match: *Oryza sativa* mitogen-activated protein kinase kinase kinase YODA isoform X1 XP_015617106.1; 79% identity; E-val: 0.0; query coverage: 74%; alignment length: 1,688). A phylogenetic tree inferred using 22 homologous protein sequences from different species showed that all *Paspalum* sequences grouped into a single cluster within the Poaceae clade, supporting the conclusion that they are allelic isoforms or, alternatively, expressed from gene copies that diverged recently (Supplementary Figure S4). Finally, the whole *QGJ* sequence was amplified by using primers located at the borders (see “Materials and Methods”), to confirm its existence without the need of computational assembly. Based on its identity as a member of the *YODA* family, we named the N46 locus *QUI-GON JINN* (*QGJ*), after another character of the Star Wars saga.

### *QGJ* Quantitative Expression in Reproductive Organs

**FIGURE 2:**
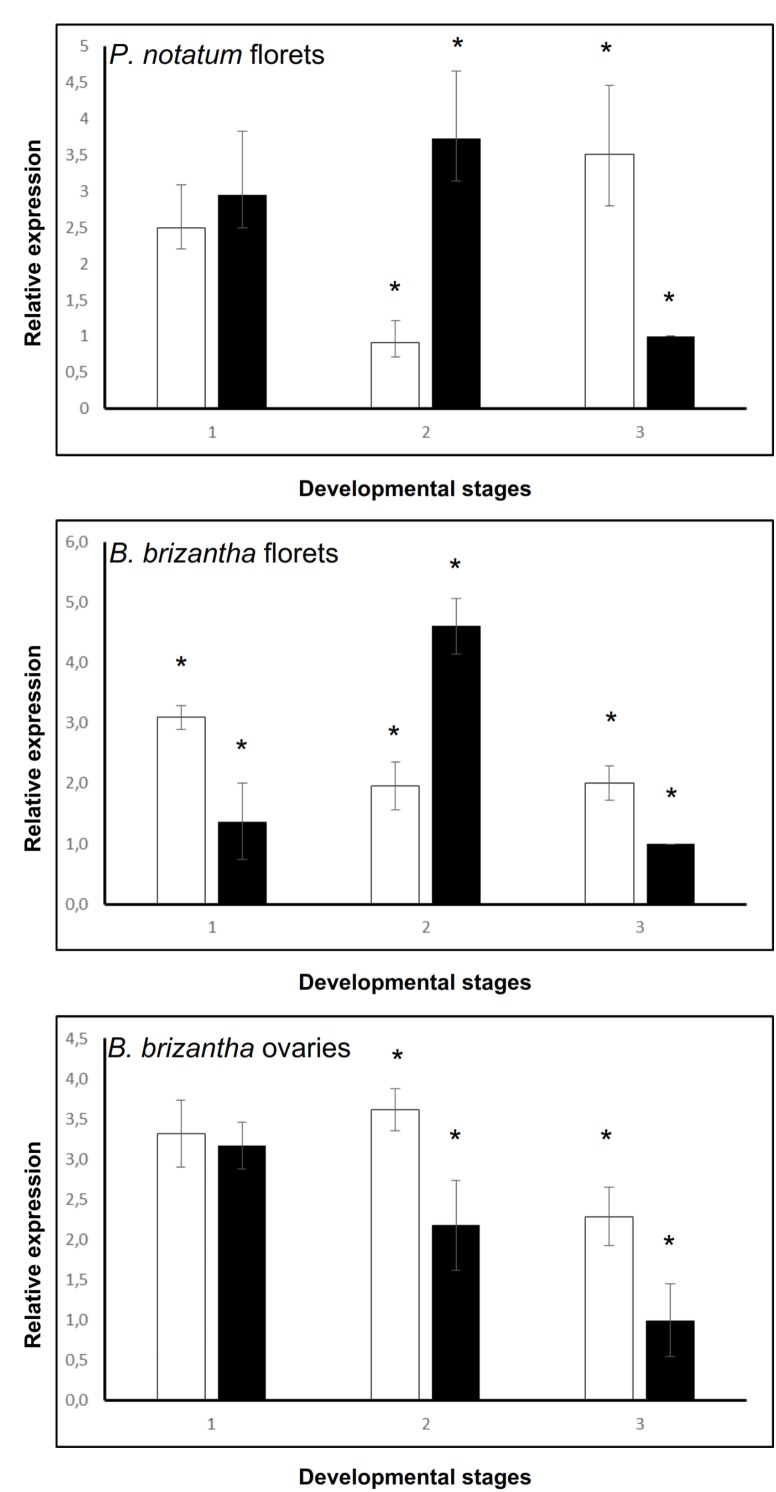
Relative quantitative expression of *QUI-GON JINN* across reproductive stages. **(A)** *P. notatum* florets. **(B)** *B. brizantha* florets. **(C)** *B. brizantha* ovaries. White bars: apomictic plant (genotype Q4117). Black bars: sexual plant (genotype C4-4x). 1: premeiosis; 2: late premeiosis/meiosis; 3: postmeiosis. Control: sexual plant, postmeiosis stage (relative expression = 1). Samples displaying statistically significant differential expression between apomictic and sexual plants were marked with an asterisk at the top.

The *QGJ* expression was quantified in spikelets of sexual and apomictic *P. notatum* genotypes at different developmental stages (1: premeiosis; 2: late premeiosis/meiosis; 3: post-meiosis) by using real-time PCR. The primer pair used for amplification was complementary to all known *QGJ* variants (see “Materials and Methods,” qPCR experiments). At stage 1 (premeiosis), *QGJ* transcripts were equally represented in both *P. notatum* reproductive types (Figure 2A). Later, during late premeiosis/meiosis, the expression in the sexual plant was significantly higher (Figure 2A). In contrast, an opposite pattern was observed at post-meiosis (Figure 2A). Besides, we took advantage of the EMBRAPA (Brasilia, Brazil) collection of *Brachiaria brizantha* plants, another well-characterized aposporous pseudogamous system (Pagliarini et al., 2012), to validate the results. *Brachiaria brizantha* (syn. *Urochloa brizantha)* is, like *P. notatum*, a rhizomatous perennial grass (Poaceae), which reproduces through aposporous pseudogamous apomixis. As *P. notatum* plants, aposporous *Brachiaria* genotypes form supernumerary non-reduced embryo sacs lacking antipodals from nucellar somatic cells surrounding the MMC. The *B. brizantha* genome also includes a single ACR lacking recombination, which may be evolutionary related to the *Paspalum* one, since it is located in a chromosomal background displaying partial synteny to rice chromosome 2 (Pessino et al., 1997, 1998). In *Brachiaria* spikelets a similar expression profile was detected, but at premeiosis overexpression was detected in the apomictic genotype (Figure 2B). However, a more detailed quantification of *QGJ* in RNA samples extracted in *Brachiaria* isolated ovaries revealed overexpression in apomictic plants at late premeiosis/meiosis (Figure 2C), which suggest the occurrence of contrasting representation patterns in different tissues.

### *In situ* Expression Pattern

**FIGURE 3:**
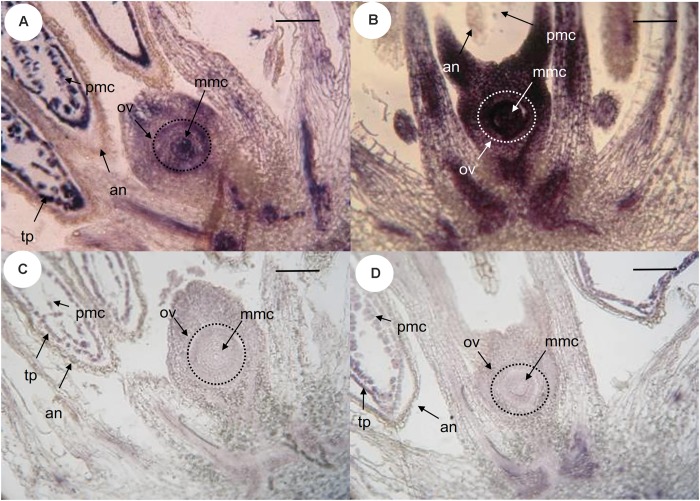
*In situ* hybridization of *QGJ* on Paspalum notatum reproductive organs at late premeiosis stage. Homologous *in situ* hybridization was carried out with the original N46 fragment. **(A,C)** Ovule and anthers of sexual plant (C4-4x), late premeiotic stage. **(B,D)** Ovule and anthers of apomictic plant (Q4117), late premeiotic stage. **(A,B)** Antisense probe. **(C,D)** Sense probe. Bars: 20 μm. References: an, anther; mmc, megaspore mother cell; ov, ovule; tp, tapetum; pmc, pollen mother cells.

**FIGURE 4:**
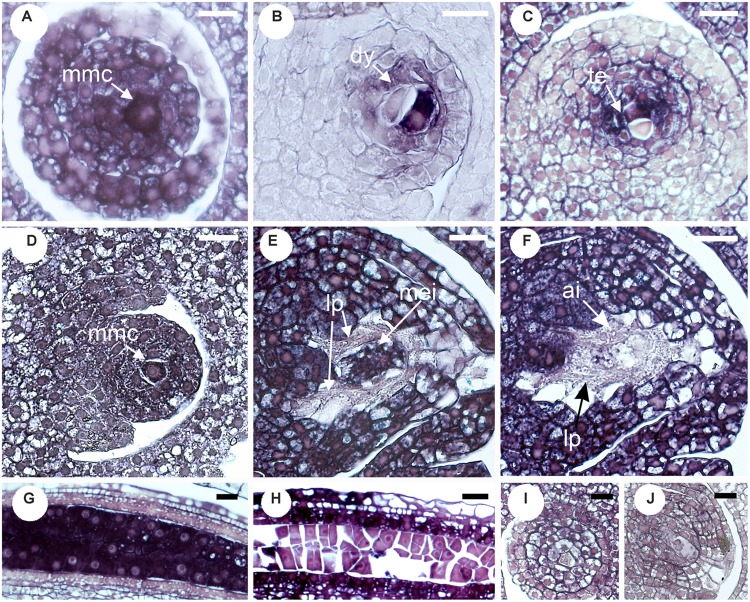
Validation of *QGJ in situ* expression in reproductive organs of aposporous *Brachiaria brizantha*. Heterologous *in situ* hybridization was carried out with the original N46 fragment. Ovules **(A–C,I)** and anther **(G)** of sexual plant B105; ovules **(D–F,J)** and anther **(H)** of apomictic plant B30. **(A–H)** Antisense probe. **(I,J)** Sense probe. **(A)** Megaspore mother cell (mmc). **(B)** Meiosis (dyad stage). **(C)** Meiosis (tetrad stage showing two megaspores). **(D)** Archesporial cell stage. **(E,F)** Meiosis (mmc entering meiosis = meiocyte) and AI formation stage. **(G,H)** Pollen mother cell stage. **(I,J)** Meiosis stage. Bars: 10 μm. References: mmc, megaspore mother cell; dy, dyad; te, tetrads; lp, layer of proximal cells surrounding the MMC; ai, apospory initial.

The site of expression was then examined through *in situ* hybridization in developing ovaries and anthers of *P. notatum*, using the original N46 clone to produce the sense and antisense probes (Figure 3). N46 is complementary to a region conserved among all *QGJ* variants, so the experiment has no potential to differentiate them. In premeiotic flowers of sexual plants, the antisense probe showed weak signal in the ovule nucellus and integuments and moderate to strong signal in the MMC, the anther tapetum and pollen mother cells (Figure 3A). Meanwhile, in apomictic plants the same probe revealed strong signal in the ovule nucellus and MMCs, and a diminished signal in anthers (Figure 3B). The sense probe showed weal signal in ovules of both sexual and apomictic plants (Figures 3C,D). We validated the observed *in situ* differential expression of *QGJ* genes in *B. brizantha* aposporous ovaries, by using the same N46 probe (Figure 4). In the *Brachiaria* experiments, thinner microtome slice cuts, together with a microscope with a higher resolving power were used (see “Materials and Methods”), allowing a more accurate detection of the hybridization pattern. In premeiotic ovules of sexual plants, a weak to moderate signal was detected in the ovule nucellus, while a moderate to strong signal appeared in the MMCs (Figure 4A). After meiosis I, the signal became mainly restricted to the micropylar cell of female dyads, yet some signal could also be observed in the nucellus (Figure 4B and Supplementary Figure S5). In tetrads, a strong signal was detected in the non-functional (micropylar) megaspores, while the functional one (located close to the chalazal end of the ovule) showed low signal (Figure 4C). In premeiotic ovules of apomictic plants, a weak to moderate signal was detected in the ovule nucellus, while a moderate to strong signal appeared in the MMCs (Figure 4D). During aposporous initials (AI) differentiation, moderate to strong signal was detected in the ovule nucellus, except for the cell layer surrounding the MMC (the AI onset site) (Figures 4E,F). Note that at this stage the MMC has enlarged and formed a meiocyte instead of entering meiosis I (meiosis frequently fails in obligate aposporous plants). Apospory initials originate from this proximal cell layer lacking signal (Figure 4F). While strong signal was detected in pollen mother cells of the sexual plant (Figure 4G), a moderate to weak signal was observed in pollen mother cells of the aposporous genotype (Figure 4H). Finally, hybridizations using sense probes detected weak signals in the nucellus of both plant types (Figures 4I,J). Our results indicate that, in sexual plants, *QGJ* is weakly expressed in nucellar tissues during meiosis. At this stage, its expression is restricted to the non-functional (micropylar) megaspores, which are adjacent to the functional (chalazal) megaspore. In contrast, and in agreement with our previous results from *Paspalum*, a strong expression of *QGJ* was observed in nucellar cells of apomictic plants. However, the proximal layer of cells originating the AI lacked signal, suggesting that *QGJ* is expressed in cells adjacent to the functional germ cells, i.e., non-functional reduced megaspores or nucellar cells located aside the non-reduced megaspores in sexual and apomictic plants, respectively.

### A Decrease of *QUI-GON JINN* Expression Impairs the Formation of Aposporous Embryo Sacs (AES)

**FIGURE 5:**
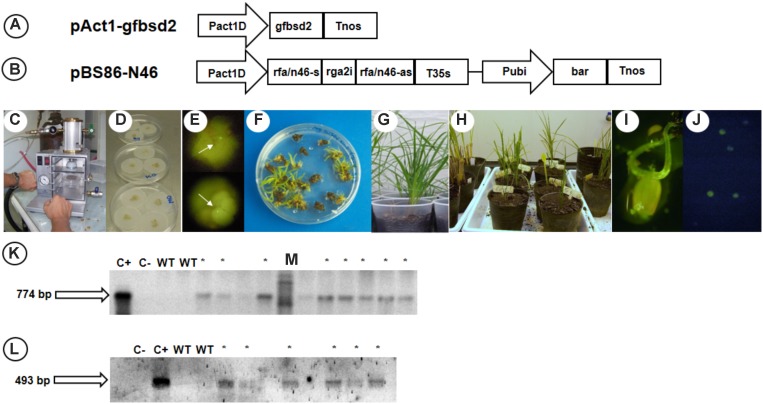
Generation of RNAi lines from apomictic genotype (Q4117). **(A,B)** Schematic representation of transformation vectors pact1-gfbsd2 and pBS86-N46. **(C)** BIOMIX biolistic device used for plant transformation as disposed previous to bombardment. **(D)** Calli in osmotic medium. **(E)** GFP expressing cells (marked by white arrows) indicating transient transformation of calli. **(F)** Regenerating plants subjected to selective agent glufosinate-ammonium 1 mg/L. **(G,H)** Rusticated plant in the growth chamber. **(I,J)** Ovary **(I)** and pollen grains **(J)** expressing *eGFP*. **(K)** PCR amplification of *eGFP* in T_0_ plants. **(L)** PCR amplification of *BAR* in T_0_ plants.

Next, we decided to investigate if a diminished expression of *QGJ* in an apomictic background gives rise to altered reproductive phenotypes. Firstly, a plant transformation vector including an N46 hairpin (pBS86-N46) was constructed by cloning the complete N46 fragment in sense and antisense orientation within plasmid pBS86 (see “Materials and Methods”). Then, *QGJ* RNAi lines were obtained by Q4117 biolistic co-transformation with plasmids pact1-gfbsd2 (which expresses an enhanced green fluorescent protein gene *eGFP* under the rice ACT1 promoter) and pBS86-N46 (see “Materials and Methods”). From 41 positive transgenic events (for pact1-gfbsd2, pBS86-N46 or both), two groups of lines were selected, which had, respectively, been transformed with: (1) the reporter plasmid pact1-gfbsd2; and (2) the RNAi plasmid pBS86-N46 and the reporter plasmid pact1-gfbsd2 (Figures 5A–H,K,L). Plants belonging to the first group were classified as transformation control lines (since they allow evaluation of reproductive phenotypes in plants subjected to *in vitro* culture and transformation procedures), while those corresponding to the second group were labeled as RNAi lines. These plants were classified as T0, since they were regenerated from bombarded calli. Prior to the molecular and cytoembryological analysis, two rounds of small rhizomes subculture were conducted (*P. notatum* is perennial and reproduces by forming rhizomes). Only four plants flowered in the isolated GMO chamber under controlled conditions (transformation control lines #TC1/#TC2 and RNAi lines #RNAi1/#RNAi2), together with a wild type control. Fluorescence analysis in transgenic lines carrying the pact1-gfbsd2 vector (both RNAi and TC) confirmed that the rice Act1 promoter drives expression in male and female reproductive tissues of *P. notatum* (Figures 5I,J).

**FIGURE 6:**
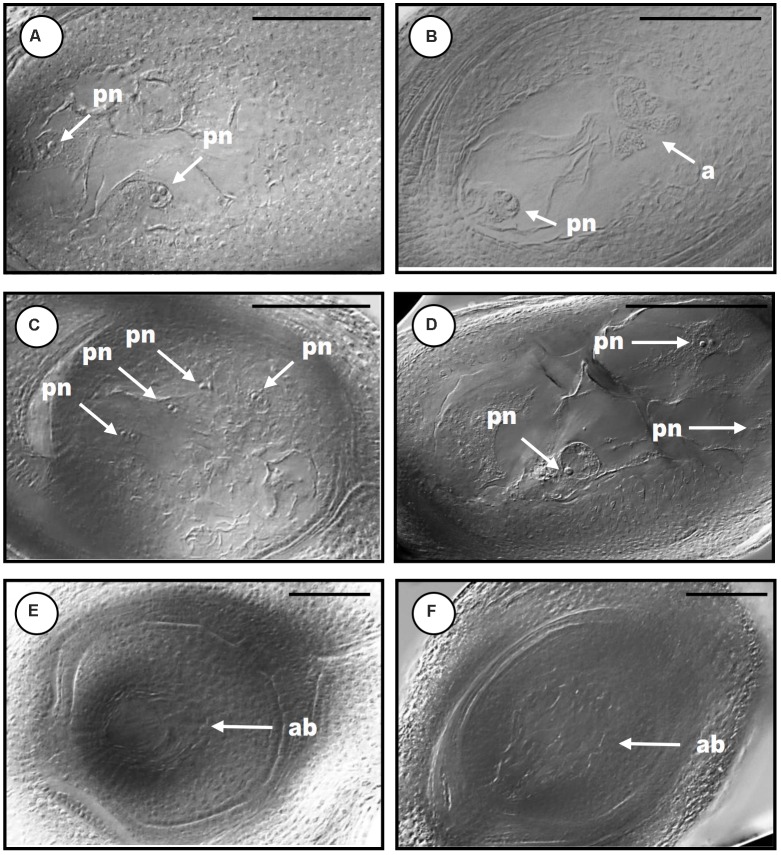
Cytoembryological analysis of the reproductive behavior in *QGJ* RNAi lines. Mature ovules of wild type genotype Q4117 **(A,B)**, transformation control lines #TC1 **(C)** and #TC2 **(D)** and *QGJ* RNAi lines #RNAi1 **(E)** and #RNAi2 **(F)**. Aposporous embryo sacs **(A,C,D)**. Meiotic embryo sac (with a proliferating mass of antipodals) **(B)**. Aborted embryo sacs **(E,F)**. References: pn, polar nuclei; a, antipodals; ab, aborted. Bars: 100 μm.

Quantitative RT-PCR analyses revealed that the *QGJ* expression was significantly attenuated in floral tissues of both RNAi lines compared to the apomictic wild type ecotype (relative expression levels ranging from 0.478 to 0.656; Supplementary Figure S6A). Lower expression levels were detected in leaves in comparison to flowers of Q4117 and no significant reduction in expression was detected in leaves of RNAi lines (Supplementary Figure S6B). Rates of viable pollen were slightly reduced in the two RNAi lines (#RNAi1 and #RNAi2) with respect to both the control lines (#TC1 and #TC2) and the wild type (Q4117) (Table 1). However, even if statistically significant, this minor alteration might not imply physiological consequences. Female reproductive development was examined by observing cleared ovules at anthesis and determining the type and number of ES per ovule. Relatively high proportions of aborted ovaries, i.e., containing no ES, were detected for lines #RNAi1 and #RNAi2 compared to both wild type and control plants (20–30% vs. <10%; Table 2). Defects in both initiation and completion of AES formation affected female reproductive development in RNAi lines (Table 2 and Figure 6). A lower number of AES per ovule was detected for both #RNAi1 and #RNAi2 (Table 2) and most of them exhibited weak/abortive phenotypes such as small size, ragged borders, and no detectable polar nuclei (Figure 6). Conversely, in control lines (#TC1 and #TC2), the average number of AES per ovule was similar to that of Q4117 (Table 2). Finally, the proportion of ovules containing meiotic ES (MES) showed no statistical difference among Q4117, control lines and RNAi lines (Table 2). We concluded that the significant reduction of *QGJ* transcripts after introducing a *QGJ* hairpin construction in the apomictic genotype Q4117 impaired the formation of AESs. Our data suggest that expression of *QGJ* in nucellar cells is necessary for aposporous development in *P. notatum*.

**Table 1:** Pollen viability of transformed and control plants.

Plant	H	E	PN	NVP	VP	PVP (95% CI)
#RNAi1	+	+	4765	636	4129	0.8675 (0.8564 < *p* < 0.876)
#RNAi2	+	+	1833	293	1540	0.8402 (0.8224 < *p* < 0.8565)
#TC1	-	+	2428	298	2130	0.8773 (0.8634 < *p* < 0.89)
#TC2	-	+	1238	118	1120	0.9106 (0.8929 < *p* < 0.9257)
#Q4117	-	-	997	100	897	0.8997 (0.879 < *p* < 0.9173)


*H, *QUI-GON* hairpin; E, *eGFP*; PN, total number of counted pollen grains; NVP, number of non-viable pollen grains; VP, number of viable pollen grains; PVP, proportion of viable pollen. 95% CI (confidence intervals) were calculated by using the Newcombe method (Newcombe, 1998), with a correction for continuity (http://vassarstats.net/prop1.html), as described in the section “Materials and Methods.”*

**Table 2:** Cytoembryological analysis of transformed and control plants.

Plant	H	E	N	Ab	V	AES	AES/N	KW (ranks/group)	MES	MES/N (95% CI)
#RNAi1	+	+	40	17	23	41	1.02	50.54/A	9	0.225 (0.114 < *p* < 0.388)
#RNAi2	+	+	43	9	34	54	1.25	57.38/A	8	0.186 (0.089 < *p* < 0.339)
#TC1	-	+	24	1	23	82	3.41	111.69/B	4	0.166 (0.0548 < *p* < 0.381)
#TC2	-	+	21	3	18	100	4.76	131.64/B	6	0.285 (0.121 < *p* < 0.523)
#Q4117	-	-	42	4	38	142	3.38	109.55/B	9	0.214 (0.108 < *p* < 0.372)


*H, *QUI-GON* hairpin; E, *eGFP*; N, number of ovules analyzed; Ab, aborted ovules (without embryo sacs); V, potentially viable ovules (with sacs); AES, total number of aposporous embryo sacs; AES/N, average number of aposporous embryo sac per ovule; KW, Test of Kruskal–Wallis comparing the AES per ovule variable; MES, total number of meiotic embryo sacs; MES/N, average number of meiotic embryo sacs per ovule; 95% CI, ninety-five % confidence intervals around MES/N proportions were calculated by using the Newcombe method (Newcombe, 1998) with a correction for continuity (http://vassarstats.net/prop1.html), as described in the section “Materials and Methods.” Aposporous embryo sacs (AES) can be readily distinguished from meiotic ones (MES) since they lack antipodal cells.*

### Genetic Linkage Analysis Between the *QGJ* Locus and the ACR

**FIGURE 7:**
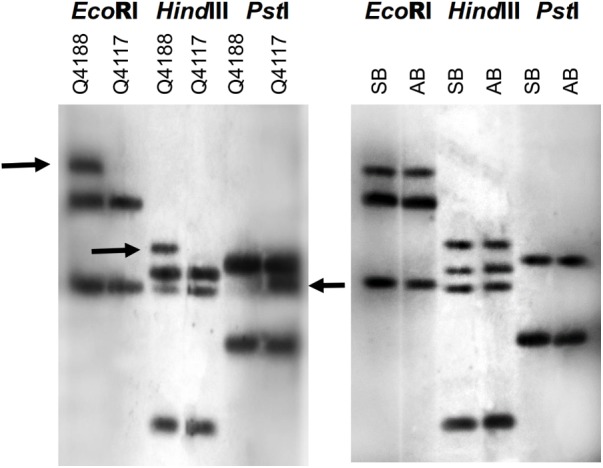
Bulked segregant analysis of the *QGJ* locus on the *P. notatum* genome. Three different restriction enzymes (*EcoR*I, *Hind*III, and *Pst*I) were used to digest the genomic DNA samples. The complete original N46 fragment was used as a probe. Left panel: Polymorphisms (marked with arrows) were detected between Q4188 (sexual genotype, mapping population female parent) and Q4117 (apomictic genotype, mapping population male parent). Right panel: Linkage analysis was performed by bulking genomic DNA extracted from 10 sexual (SB) and 10 apomictic (AB) F_1_ individuals obtained from crossing Q4188 (female) and Q4117 (male).

Possible co-segregation of the *QGJ* locus with apospory was examined in *P. notatum* by using bulked-segregant analysis (Figure 7). The complete N46 original fragment was hybridized onto genomic DNA samples originated from two parental plants (Q4188, sexual female parent and Q4117, apomictic pollen donor) and genomic DNA bulks made from 10 sexual and 10 apomictic F_1_ plants derived from the Q4188 × Q4117 cross. Segregation in F_1_ is expected due to ACR hemizygosity and the heterozygous nature of a high number of parental genomic loci. Although genomic DNA digestion with three different restriction enzymes (*Eco*RI, *Hind*III, and *Pst*I) produced several polymorphic bands between parental plants, none of them resulted polymorphic between F_1_ bulks. The bulked results show the sexual polymorphisms are not specific to sexual F_1_s. However, the apomictic band present in the parental PstI digest disappeared in the apomictic F_1_s. Since PstI is sensitive to certain contexts (CpNpG sites), this observation might suggest a methylation change occurring during hybridization. *In silico* mapping onto the Gramene website revealed that the putative ortholog to *QGJ* is located in chromosome 11 (Os11g0207200, E-value 0.0), in a genomic region showing no synteny with the ACR of *P. notatum*. However, other high score hits (Os02g0555900, Os02t0666300-01, Os12g0577700, E-values 5.7E-86, 3E-58, and 7.6E-15) are protein kinase genes located in a rice genome region (chromosome 2 long arm, positions: 21,002,439–21,008,209, 27,048,920–27,061,955 and chromosome 12 long arm, positions: 23,885,845–23,888,835, respectively) syntenic to the *P. notatum* ACR. Our results showed no evidence of a genetic link between *QUI-GON JINN* and the ACR, but suggested that sequences showing significant similarity to this gene might be located within the genomic region controlling apospory.

### The *Paspalum* ACR Transcribes a Long Non-coding Sequence Showing Partial Similarity to *QGJ*

In a previous work, Polegri et al. (2010) reported full linkage between the *Paspalum simplex* transcriptome fragment A-148-3 and apospory, and determined that this candidate was also homologous to At1g53570. A search for the complete sequence of A-148-3 in the *Paspalum* Roche 454 floral libraries revealed that it was not a *PN_QGJ* ortholog, since it showed the best hit of similarity with apoisotig 04689 (GFMI02004742.1) (query cover 100%; E-value: 6 e^-143^, identities 90%; position on apoisotig 04689: 5588–5955). Interestingly, apoisotig 04689 is a 6835-nt sequence with no coding potential and specific to the apomictic *P. notatum* floral transcriptome libraries (reads in the apomictic library: 133; reads in the sexual library: 0). A search in the GreeNC database revealed similarity with six predicted plant lncRNAs at E-values ≤ 1 e^-10^ (best match: Zmays_GRMZM2G024551_T01; E-value: 1.00411e^-27^; alignment length: 226 nt, involving the segment flanked by positions 4304–4530 within apoisotig 04689; positive: 177). Given its partial similarity with *QGJ*, its lack of protein-coding potential, its similarity to sequences included in the GreeNC database and its detected expression in the apomictic floral transcriptome, we inferred that isotig 04689 is a long non-coding RNA (lncRNA) related to *QGJ*, and was renamed accordingly as *PN_LNC_QGJ* (after *P. notatum* long non-coding *QGJ*). The complete *QGJ* functional sequence (apoisotig 03085, 2377 nt) has similarity to the *PN_LNC_QGJ* transcript in positions ranging 1033–1326/1352–1475 (matching positions 5493–5800/6255–6387 in the *PN_LNC_QGJ* sequence) (Figure 1). Meanwhile, the original N46 fragment spanned positions 733–1174 in the *QGJ* sequence (apoisotig 03085) (Figure 1).

**FIGURE 8:**
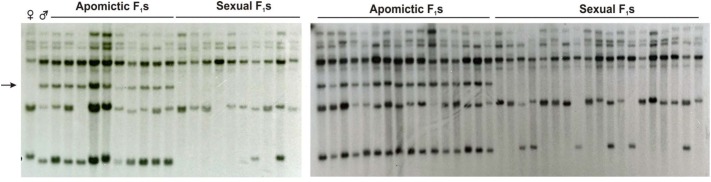
A-148-3 mapping onto the *P. notatum* genome by RFLP analysis. Hybridizing banding pattern of genomic DNA *Eco*RI digests of a *P. notatum* segregating population, composed of a female sexual parent (Q4188,♀), a male aposporous parent (Q4177, ♂) together with 29 sexual and 26 aposporous F_1_ plants. The arrow indicates a band inherited from the aposporous parent, which strictly co-segregates with apospory.

Mapping of the A-148-3 original transcript in a *P. notatum* population (55 F_1_ plants) revealed one polymorphic band strictly cosegregating with apomixis (Figure 8). Another band showed partial linkage, confirming association of the sequence with proximal regions. Besides, the two additional monomorphic/non-segregating bands were detected, which could be related with the same locus or, alternatively, with other genomic regions located elsewhere (Figure 8). Furthermore, reverse-transcribed PCR experiments with *PN_LNC_QGJ* specific primers conducted in several apomictic and sexual *P. notatum* individuals showed that *PN_LNC_QGJ* is expressed only in apomictic plants (Supplementary Figure S7).

Based on these observations, we concluded that original transcript fragment A-148-3 is part of a long non-coding RNA (namely *PN_LNC_QGJ*) differentially expressed in apomictic and sexual plants. Besides, a sequence similar to the A-148-3 probe is located in the *P. notatum* ACR. However, further experiments should be conducted to determine if the copy of the gene located at the ACR is producing the differentially expressed lncRNA (considering the existence of monomorphic bands, which could represent copies located in other parts of the genome). Moreover, the existence of a functional link between the altered *QGJ* expression and a possible regulator activity of *PN_LNC_QGJ* has yet to be investigated through functional analysis. The alignments between the *Z. mays* reference genomic sequence GRMZM6G513881 and the sequences of all *PN_QGJ* and *PN_LNC_QGJ* isotigs are displayed in Supplementary Figure S8.

## Discussion

The extracellular signal-regulated kinase 1/2 (ERK1/2) cascade is a central signaling pathway that modulates a wide variety of cellular processes, including proliferation, differentiation, survival, apoptosis, and stress response (Wortzel and Seger, 2011). The intracellular communication between membrane receptors and their nuclear or cytoplasmic targets upon stimulation is mediated by a limited number of signaling pathways, including a group of mitogen-activated protein kinase (MAPK) cascades (Wortzel and Seger, 2011). MAPK signal transduction cascades consist of three sequentially activated kinases. Upstream signals activate MAPK kinase kinases (MAPKKKs), which in turn phosphorylate MAPK kinases (MKKs); subsequently, MKKs activate specific MAPKs. The downstream targets of MAPKs can be either transcription factors or cytoskeletal proteins. Phosphorylation and activation of a MAPK can lead to changes in its subcellular localization and its activity on transcriptional effectors, thereby reprogramming gene expression (Fiil et al., 2009). In particular, the *Arabidopsis* genome encodes 60 putative MAP3Ks (including the MEKK, RAF, and ZIK subfamilies), 10 MAPKKs, and 20 MAPKs, involved in a plethora of different responses to specific ligands (Ichimura et al., 2002). Here, we characterized the expression and function of *QUI-GON JINN* (*QGJ*), the putative ortholog to *Arabidopsis* At1g53570/*AtMEKK3*, in reproductive organs of sexual and apomictic *P. notatum* plants. *AtMEKK3* belongs to the MEKK subfamily related to budding yeast Ste11p (Lukowitz et al., 2004). It comprises 12 members (Ichimura et al., 2002), including critical regulators of plant cell division and differentiation during reproduction, i.e., YODA/AtMAPKKK4 (early embryogenesis) (Lukowitz et al., 2004), AtMEKK20 (male gamete differentiation) (Borg et al., 2011) and ScFRK/AtMEKK19/20 (female gametophyte development) (Daigle and Matton, 2015).

Our results suggests that *QGJ* plays a role in promoting the acquisition of a gametophytic cell fate by AIs, a critical step in the establishment of the aposporous pathway, or alternatively affects the development of the embryo sacs. The fact that *QGJ* is not expressed in the cell layer originating AIs, but in the adjacent ones, suggests that a non-cell-autonomous signaling mechanism might be operating. Such mechanisms are commonly used for intercellular communication during plant development (Van Norman et al., 2011). In contrast, pollen viability and meiotic embryo sac formation seem poorly affected in RNAi plants, showing that the *QGJ* partial silencing is not influencing male/female meiosis in an obligate apomictic background. However, since the meiotic pathway is *per se* diminished in obligate apomictic plants (the Q4117 apomictic genotype naturally shows a rate of meiotic embryo sac development of around 3–4%) (Ortiz et al., 1997), it is difficult to evaluate the *QGJ* post-transcriptional attenuation consequences on the sexual developmental pathway when using the genetic background transformed here. Such analyses should be conducted in a mutant/transformant line derived from a sexual plant, an experiment that we plan to complete in the near future. Moreover, the slight decrease in pollen viability detected in the *Paspalum* transgenic lines might reflect a mild response to the introduction of the hairpin in a tissue where the gene is naturally down-regulated with respect to sexual plants, as revealed by *in situ* hybridization. Podio et al. (2012) had analyzed anaphase I configurations and pollen viability in aposporous and sexual tetraploid cytotypes of *P. notatum* and found reduced pollen viability in the aposporous genotypes, including Q4117. A reduced activity of *QGJ* in pollen mother cells of aposporous plants (Figures 3, 4) could also explain the diminished expression detected in apo plants in comparative qPCR experiments conducted on florets (Figure 2), since anthers represent a high proportion of the floret tissue at this stage. Based on all these observations, we hypothesized that, similarly to *YODA* (Lukowitz et al., 2004), *QGJ* mediates a signaling pathway acting as a key regulator to define cell lineage during plant reproduction. While *YODA* is required for normal development of the zygote and the cells of the basal lineage originating the suspensor, *QGJ* might play a role during the sporophytic-to-gametophytic transition phase. However, although our results indicate that *QGJ* activity is essential to non-reduced embryo sacs formation, overexpression experiments under ovule specific promoters will be necessary to assess if expression in the nucellus of sexual plants is fully responsible for apomictic development. An alternative hypothesis is that this candidate is involved later, after the fate decision has been made, in either sexual or aposporous embryo formation.

Polegri et al. (2010) reported the isolation of A-148-3, a *P. simplex* transcript homologous to the predicted *Arabidopsis QGJ* ortholog (At1g53570), showing constitutive expression in apomictic genotypes during all reproductive developmental stages and linkage to the *P. simplex* ACR. However, full sequence analysis revealed that A-148-3 is not a *QGJ* ortholog, but an lncRNA with partial similarity to *QGJ*. Genetic linkage analyses in a Q4188 (sexual) × Q4117 (apomictic) F_1_ population confirmed that a sequence showing similarity with A-148-3 is located within the *P. notatum* ACR. On the contrary, we found no evidence that N46 and the ACR are genetically linked. Note that the A-148.3 probe has some potential to hybridize *QGJ* (the original 354 bp-long A-148-3 sequence includes a 147-nt insertion with 78 % similarity to *P. notatum QGJ*). Moreover, from the 451 nt covered by the original fragment N46, a 112-nucleotide segment keeps partial similarity (72%) with *PN_LNC_QGJ*. Although these similarities are limited, involve only a portion of the probes (40% of A-148.3 and 25% of N46), and the experimental conditions were strict enough to ensure specific detection, the possibility of some cross hybridization cannot be fully discarded, especially for A-148.3. However, the detection of contrasting genomic hybridization patterns when using N46 and A-148.3 as alternative probes (Figures 7, 8), suggests that each of them has capacity for specific detection. The ACR is a genomic region specific to apomictic genotypes, which is highly heterochromatic and harbors almost intact exonic sequences interlaced within highly repetitive sequences (Podio et al., 2014a). *PN_LNC_QGJ* is expressed in floral tissues of aposporous plants only, and it includes large, non-coding stretches similar to retrotransposons and two short exonic *QGJ* regions (439 nt in total out of 6835). Long non-coding RNAs have recently emerged as critical regulators of gene expression in many eucaryotes, including plants (Ariel et al., 2015; Chekanova, 2015; Liu et al., 2015). Therefore, considering the sequence relationship between *QGJ* and *PN_LNC_QGJ*, it is tempting to speculate that *PN_LNC-QGJ* could mediate *QGJ* modulation in reproductive tissues of *Paspalum* apomicts. Among many putative mechanisms, our results point out at least two of them: (1) a change in splicing leading to the formation of variants; and (2) the induction of nucellar expression via miRNA hijacking. However, no claim of a functional relationship between *QGJ* and *PN_LNC_QGJ* can be currently made, since it is not supported by functional analysis data. Moreover, expression of *PN_LNC_QGJ* from the ACR should be further confirmed. Shortly, we will focus in determining if the miss-expression of *QGJ* is caused by a transcriptional regulatory event or is alternatively influenced by *PN_LNC-QGJ*, and to determine if *PN_LNC-QGJ* is expressed from the ACR. Interestingly, non-coding transcripts carrying exonic sequences were proposed to regulate *PN_SERK* and *PS_ORC3*, two genes putatively involved in apomixis in *P. notatum* and *P. simplex*, respectively (Podio et al., 2014b; Siena et al., 2016).

In the past decade, numerous candidate genes for apomixis were identified (Hand and Koltunow, 2014; Ronceret and Vielle-Calzada, 2015) but how the underlying networks integrate into sexual reproduction and alter expression patterns remain largely unknown. Our work posits that a MAP3K signaling pathway of an ERK1/2 cascade is pivotal to aposporous embryo sac differentiation. However, the rest of the members of the ERK cascade and their interactions with this kinase remain unknown. Interestingly, besides N46 (*QGJ*), Laspina et al. (2008) reported the differential expression of several other genes involved in ERK cascades in comparisons between sexual and apomictic plants: an LRR family protein (N79), a GPI anchored protein (N20), phosphatidylinositol 4K (N23), a Ser/Thr phosphatase (N102), the PRIP-interacting protein (N69) and a kinesin (N114). From them, only N20 and N69 have been further characterized (Felitti et al., 2011; Siena et al., 2014). N20 (later renamed *N20GAP-1*) is ortholog to genes At4g26466 (*LORELEI*, encoding a GPI-anchored protein) and/or At5g56170 (*LORELEI*-like), shows partial cosegregation with apospory and is increasingly overexpressed in apomictic plants from premeiosis to antesis (Felitti et al., 2011). N69 is ortholog to gene AT1G45231 (*TGS1*, encoding a trimethylguanosine synthase which has a dual role in splicing and transcription), and, contrarily, is increasingly overexpressed in sexual plants from premeiosis to antesis (Siena et al., 2014). Moreover, evidence was shown that TGS1 Ser^298^ phosphorylation is promoted by an ERK cascade to activate transcriptional activity at some promoters (Kapadia et al., 2013). The availability of *Paspalum* RNAi lines for N20 (*LORELEI*), N69 (*TGS1*) and N46 (*QGJ* MAP3K, reported here) would allow to investigate in detail a possible biological link among these molecules. Though functional approaches are challenging in polyploid, highly heterozygous apomictic species like *P. notatum*, the development of reference genomes, transformation protocols and advanced microscopy tools will likely accelerate the discovery of the central mechanisms underlying the switch from sexuality to apomixis.

## Author Contributions

MM: transformation experiments and the genotypic/phenotypic analysis. HP: transformation experiments supervision. CC and CA: RNA extractions, real time experiments, and bioinformatic analysis. LS: developmental stage classification and RNA extraction. FP: PN_LNC_QGJ mapping. DdAD, VdCC, and MP: *Brachiaria in situ* hybridization. MP: PN_QGJ genomic hybridization. JS and AG: *Paspalum in situ* hybridization. SF: transformation vector construction. JO: phylogenetic analysis and experimental design. OL: experimental design, sequence analysis, and manuscript writing. SP: experimental design, *in silico* analysis, qPCR analysis, and manuscript writing.

## Conflict of Interest Statement

The authors declare that the research was conducted in the absence of any commercial or financial relationships that could be construed as a potential conflict of interest. The handling Editor is currently organizing a Research Topic with one of the authors FP, and confirms the absence of any other collaboration.

## Footnotes

1 http://www.ncbi.nlm.nih.gov/BLAST/

2 https://www.arabidopsis.org/Blast/index.jsp

3 www.gramene.org

4 http://rice.plantbiology.msu.edu/annotation_oryza.shtml

5 http://www.ncbi.nlm.nih.gov/gorf/gorf.html

6 http://wormweb.org/exonintron

7 http://vassarstats.net/prop1.html

8 http://cran.r-project.org/doc/contrib/Owen-TheRGuide.pdf

## References

[B1] Albertini E. Marconi G. Barcaccia G. Raggi L. Falcinelli M. (2004). Isolation of candidate genes for apomixis in *Poa pratensis*. *Plant Mol. Biol.* 56 879–894. 10.1007/s11103-004-5211-y 15821987

[B2] Alexander M. P. (1980). Differential staining of aborted and non-aborted pollen. *Stain Technol.* 44 117–122. 10.3109/10520296909063335 4181665

[B3] Ariel F. Romero-Barrios N. Jégu T. Benhamed M. Crespi M. (2015). Battles and hijacks: noncoding transcription in plants. *Trends Plant Sci.* 20 362–371. 10.1016/j.tplants.2015.03.003 25850611

[B4] Bicknell R. A. Koltunow A. M. (2004). Understanding apomixis: recent advances and remaining conundrums. *Plant Cell* 16 228–245. 10.1105/tpc.017921 15131250 PMC2643386

[B5] Borg M. Brownfield L. Khatab H. Sidorova A. Lingaya M. Twell D. (2011). The R2R3 MYB transcription factor DUO1 activates a male germline-specific regulon essential for sperm cell differentiation in *Arabidopsis*. *Plant Cell* 23 534–549. 10.1105/tpc.110.081059 21285328 PMC3077786

[B6] Carman J. G. (1997). Asynchronous expression of duplicate genes in angiosperms may cause apomixis, bispory, tetraspory, and polyembryony. *Biol. J. Linn. Soc.* 61 51–94. 10.1111/j.1095-8312.1997.tb01778.x

[B7] Cervigni G. D. Paniego N. Díaz M. Selva J. P. Zappacosta D. Zanazzi D. (2008). Expressed sequence tag analysis and development of associated markers in a near-isogenic plant system of *Eragrostis curvula*. *Plant Mol. Biol.* 67 7–10. 10.1007/s11103-007-9282-4 18196464

[B8] Chekanova J. A. (2015). Long non-coding RNA and their functions in plants. *Curr. Opin. Plant Biol.* 27 207–216. 10.1016/j.pbi.2015.08.003 26342908

[B9] Daigle C. Matton D. P. (2015). Genome-wide analysis of MAPKKKs shows expansion and evolution of a new MEKK class involved in solanaceous species sexual reproduction. *BMC Genomics* 16:1037. 10.1186/s12864-015-2228-3 26645086 PMC4673785

[B10] Felitti S. A. Seijo J. G. González A. M. Podio M. Laspina N. V. Siena L. (2011). Expression of *lorelei*-like genes in aposporous and sexual *Paspalum notatum* plants. *Plant Mol. Biol.* 77 337–354. 10.1007/s11103-011-9814-9 21826430

[B11] Fiil B. K. Petersen K. Petersen M. Mundy J. (2009). Gene regulation by MAP kinase cascades. *Curr. Opin. Plant Biol.* 12 615–621. 10.1016/j.pbi.2009.07.017 19716758

[B12] Garcia-Aguilar M. Michaud C. Leblanc O. Grimanelli D. (2010). Inactivation of a DNA methylation pathway in maize reproductive organs results in apomixis-like phenotypes. *Plant Cell* 22 3249–3267. 10.1105/tpc.109.072181 21037104 PMC2990141

[B13] Hand M. Koltunow A. (2014). The genetic control of apomixis: asexual seed formation. *Genetics* 197 441–450. 10.1534/genetics.114.163105 24939990 PMC4063905

[B14] Ichimura K. Shinozaki K. Tena G. Sheen J. Henry Y. Champion A. (2002). Mitogen-activated protein kinase cascades in plants: a new nomenclature. *Trends Plant Sci.* 7 301–308. 10.1016/S1360-1385(02)02302-6 12119167

[B15] Kapadia B. Viswakarma N. Parsa K. V. L. Kain V. Behera S. Suraj S. K. (2013). ERK2-mediated phosphorylation of transcriptional coactivator binding protein PIMT/NcoA6IP at Ser298 augments hepatic gluconeogenesis. *PLoS One* 8:e83787. 10.1371/journal.pone.0083787 24358311 PMC3866170

[B16] Kruskal W. H. Wallis W. A. (1952). Use of ranks in one-criterion variance analysis. *J. Am. Stat. Assoc.* 47 583–621. 10.1080/01621459.1952.10483441

[B17] Larkin M. A. Blackshields G. Brown N. P. Chenna R. MCGettigan P. A. McWilliam H. (2007). Clustal W and Clustal X version 2.0. *Bioinformatics* 23 2947–2948. 10.1093/bioinformatics/btm404 17846036

[B18] Laspina N. V. Vega T. Martelotto L. Stein J. Podio M. Ortiz J. P. (2008). Gene expression analysis at the onset of aposporous apomixis in *Paspalum notatum*. *Plant Mol. Biol.* 67 615–628. 10.1007/s11103-008-9341-5 18481185

[B19] Liu X. Hao L. Li D. Zhu L. Hu S. (2015). Long non-coding RNAs and their biological roles in plants. *Genomics Proteomics Bioinformatics* 13 137–147. 10.1016/j.gpb.2015.02.003 25936895 PMC4563214

[B20] Lukowitz W. Roeder A. Parmenter D. Somerville C. (2004). A MAPKK kinase gene regulates extra-embryonic cell fate in *Arabidopsis*. *Cell* 116 109–119. 10.1016/S0092-8674(03)01067-5 14718171

[B21] Mancini M. Woitovich N. Permingeat H. Podio M. Siena L. A. Ortiz J. P. A. (2014). Development of a modified transformation platform for apomixis candidate genes research in *Paspalum notatum* (bahiagrass). *In Vitro Cell. Dev. Biol. Plant* 50 412–424. 10.1007/s11627-014-9596-2

[B22] Musielak T. J. Bayer M. (2014). YODA signalling in the early *Arabidopsis* embryo. *Biochem. Soc. Trans.* 42 408–412. 10.1042/BST20130230 24646252

[B23] Newcombe R. G. (1998). Two-sided confidence intervals for the single proportion: comparison of seven methods. *Stat. Med.* 17 857–872. 10.1002/(SICI)1097-0258(19980430)17:8<857::AID-SIM777>3.0.CO;2-E 9595616

[B24] Nogler G. A. (1984). “Gametophytic apomixis,” in *Embryology of Angiosperms*, ed. Johri B. M. (Berlin, Germany: Springer-Verlag), 475–518.

[B25] Ochiai-Fukuda T. Takahashi-Ando N. Ohsato S. Igawa T. Kadokura K. Hamamoto H. (2006). A fluorescent antibiotic resistance marker for rapid production of transgenic rice plants. *J. Biotechnol.* 122 521–527. 10.1016/j.jbiotec.2005.09.015 16271791

[B26] Ochogavía A. C. Seijo J. G. González A. M. Podio M. Laspina N. V. Duarte Silveira E. (2011). Characterization of retrotransposon sequences expressed in inflorescences of apomictic and sexual *Paspalum notatum* plants. *Sex. Plant Reprod.* 24 231–246. 10.1007/s00497-011-0165-0 21394488

[B27] Okada T. Hu Y. Tucker M. R. Taylor J. M. Johnson S. D. Spriggs A. (2013). Enlarging cells initiating apomixis in *Hieracium praealtum* transition to an embryo sac program prior to entering mitosis. *Plant Physiol.* 163 216–231. 10.1104/pp.113.219485 23864557 PMC3762643

[B28] Ortiz J. P. Pessino S. C. Leblanc O. Hayward M. D. Quarin C. L. (1997). Genetic fingerprint for determinig the mode of reproduction in *Paspalum notatum*, a subtropical apomictic forage grass. *Theoret. Appl. Genet.* 95 850–856. 10.1007/s001220050635

[B29] Ortiz J. P. A. Quarin C. L. Pessino S. C. Acuña C. Martínez E. J. Espinoza F. (2013). Harnessing apomictic reproduction in grasses: what we have learned from *Paspalum*. *Ann. Bot.* 112 767–787. 10.1093/aob/mct152 23864004 PMC3747805

[B30] Ortiz J. P. A. Revale S. Siena L. A. Podio M. Delgado L. Stein J. (2017). A reference floral transcriptome of sexual and apomictic *Paspalum notatum*. *BMC Genomics* 18:318. 10.1186/s12864-017-3700-z 28431521 PMC5399859

[B31] Pagliarini M. S. Carneiro Vieira M. L. Borges do Valle C. (2012). “Meiotic behavior in intra- and interspecific sexual and somatic polyploid hybrids of some tropical species,” in *Meiosis Molecular Mechanisms and Cytogenetic Diversity*, ed. Swan A. (London: Intech Open). 10.5772/30518

[B32] Paterson A. H. Brubaker C. L. Wendel J. F. (1993). A rapid method for extraction of cotton (*Gossypium spp*.) genomic DNA suitable for RFLP or PCR analysis. *Plant Mol. Biol. Rep.* 11 122–127. 10.1007/BF02670470

[B33] Paytuví Gallart A. Hermoso Pulido A. Martínez de Lagrán I. A. Sanseverino W. Aiese Cigliano R. (2016). GREENC: a Wiki-based database of plant lncRNAs. *Nucleic Acids Res.* 44 1161–1166. 10.1093/nar/gkv1215 26578586 PMC4702861

[B34] Pessino S. C. Espinoza F. Martínez E. J. Ortiz J. P. A. Valle E. M. Quarin C. L. (2001). Isolation of cDNA clones differentially expressed in flowers of apomictic and sexual *Paspalum notatum*. *Hereditas* 134 35–42. 10.1111/j.1601-5223.2001.00035.x 11525063

[B35] Pessino S. C. Evans C. Ortiz J. P. A. Armstead I. do Valle C. B. Hayward M. D. (1998). A genetic map of the apospory region in *Brachiaria hybrids*: identification of two markers closely associated with the trait. *Hereditas* 128 153–158. 10.1111/j.1601-5223.1998.00153.x

[B36] Pessino S. C. Ortiz J. P. A. Leblanc O. do Valle C. B. Evans C. Hayward M. D. (1997). Identification of a maize linkage group related to apomixis in *Brachiaria*. *Theoret. Appl. Genet.* 94 439–444. 10.1007/s001220050434

[B37] Podio M. Cáceres M. E. Samoluk S. S. Seijo J. G. Pessino S. C. Ortiz J. P. A. (2014a). A methylation status analysis of the apomixis specific region in *Paspalum spp.* suggests an epigenetic control of parthenogenesis. *J. Exp. Bot.* 65 6411–6424. 10.1093/jxb/eru354 25180110

[B38] Podio M. Felitti S. A. Siena L. A. Delgado L. Mancini M. Seijo G. (2014b). Characterization and expression analysis of *SOMATIC EMBRYOGENESIS RECEPTOR KINASE* (SERK) genes in sexual and apomictic *Paspalum notatum*. *Plant Mol. Biol.* 84 479–495. 10.1007/s11103-013-0146-9 24146222

[B39] Podio M. Siena L. A. Hojsgaard D. Stein J. Quarin C. L. Ortiz J. P. A. (2012). Evaluation of meiotic abnormalities and pollen viability in aposporous and sexual tetraploid *Paspalum notatum* (Poaceae). *Plant Syst. Evol.* 298 1625–1633. 10.1007/s00606-012-0664-y

[B40] Polegri L. Calderini O. Arcioni S. Pupilli F. (2010). Specific expression of apomixis-linked alleles revealed by comparative transcriptomic analysis of sexual and apomictic *Paspalum* simplex Morong flowers. *J. Exp. Bot.* 61 1869–1883. 10.1093/jxb/erq054 20231327

[B41] Pupilli F. Labombarda P. Cáceres M. E. Quarin C. L. Arcioni S. (2001). The chromosome segment related to apomixis in Paspalum simplex is homoeologous to the telomeric region of the long arm of rice chromosome 12. *Mol. Breed.* 8 53–61. 10.1023/A:1011966922301

[B42] Quarin C. L. Espinoza F. Martínez E. J. Pessino S. C. Bovo O. A. (2001). A rise of ploidy level induces the expression of apomixis in *Paspalum notatum*. *Sex. Plant Reprod.* 13 243–249. 10.1007/s004970100070

[B43] Quarin C. L. Urbani M. H. Blount A. R. Martínez E. J. Hack C. M. Burton G. W. (2003). Registration of Q4188 and Q4205, sexual tetraploid germplasm lines of Bahiagrass. *Crop Sci.* 43 745–746. 10.2135/cropsci2003.0745

[B44] Rodrigues J. C. Cabral G. B. Dusi D. M. A. Mello L. V. Rinden D. Carneiro V. T. C. (2003). Identification of differentially expressed cDNA sequences in ovaries of sexual and apomictic plants of *Brachiaria brizantha*. *Plant Mol. Biol.* 53 745–757. 10.1023/B:PLAN.0000023664.21910.bd 15082923

[B45] Ronceret A. Vielle-Calzada J. P. (2015). Meiosis, unreduced gametes, and parthenogenesis: implications for engineering clonal seed formation in crops. *Plant Reprod.* 28 91–102. 10.1007/s00497-015-0262-6 25796397

[B46] Siena L. A. Ortiz J. P. A. Leblanc O. Pessino S. (2014). PnTgs1-like expression during reproductive development supports a role for RNA methyltransferases in the aposporous pathway. *BMC Plant Biol.* 14:297. 10.1186/s12870-014-0297-0 25404464 PMC4243328

[B47] Shapiro S. S. Wilk M. B. (1965). An analysis of variance test for normality (complete samples). *Biometrika* 52 591–611. 10.1093/biomet/52.3-4.591

[B48] Sharbel T. F. Voigt M. L. Corral J. M. Galla G. Kumlehn J. Klukas C. (2010). Apomictic and sexual ovules of *Boechera* display heterochronic global gene expression patterns. *Plant Cell* 22 655–671. 10.1105/tpc.109.072223 20305122 PMC2861462

[B49] Sharbel T. F. Voigt M. L. Corral J. M. Thiel T. Varshney A. Kumlehn J. (2009). Molecular signatures of apomictic and sexual ovules in the *Boechera holboellii* complex. *Plant J.* 58 870–882. 10.1111/j.1365-313X.2009.03826.x 19220792

[B50] Siena L. A. Ortiz J. P. A. Calderini O. Paolocci F. Cáceres M. E. Kaushal P. (2016). An apomixis-linked *ORC3*-like pseudogene is associated with silencing of its functional homolog in apomictic *Paspalum simplex*. *J. Exp. Bot.* 67 1965–1978. 10.1093/jxb/erw018 26842983

[B51] Sneath P. H. A. Sokal R. R. (1973). “The estimation of taxonomic resemblance,” in *Numerical Taxonomy. The Principles and Practice of Numerical Classification*, eds Kennedy D. Park R. B. (San Francisco, CA: Freeman), 129–132.

[B52] Stein J. Pessino S. C. Martínez E. J. Rodríguez M. P. Siena L. A. Quarin C. L. (2007). A genetic map of tetraploid *Paspalum notatum* Flügge (bahiagrass) based on single-dose molecular markers. *Mol. Breed.* 20 153–166. 10.1007/s11032-007-9083-0

[B53] Tamura K. Stecher G. Peterson D. Filipski A. Kumar S. (2013). MEGA6: molecular evolutionary genetics analysis Version 6.0. *Mol. Biol. Evol.* 30 2725–2729. 10.1093/molbev/mst197 24132122 PMC3840312

[B54] Thompson C. J. Movva N. R. Tizard R. Crameri R. Davies J. E. Lauwereys M. (1987). Characterization of the herbicide-resistance gene bar from *Streptomyces hygroscopicus*. *EMBO J.* 6 2519–2523. 10.1002/j.1460-2075.1987.tb02538.x 16453790 PMC553668

[B55] Van Norman J. M. Breakfield N. W. Benfey P. N. (2011). Intercellular communication during plant development. *Plant Cell* 23 855–864. 10.1105/tpc.111.082982 21386031 PMC3082268

[B56] Wilcoxon F. (1945). Individual comparisons by ranking methods. *Biometrics Bull.* 1 80–83. 10.2307/3001968

[B57] Wilson E. B. (1927). Probable inference, the law of succession, and statistical inference. *J. Am. Stat. Assoc.* 22 209–212. 10.1080/01621459.1927.10502953

[B58] Wortzel I. Seger R. (2011). The ERK cascade: distinct functions within various subcellular organelles. *Genes Cancer* 2 195–209. 10.1177/1947601911407328 21779493 PMC3128630

[B59] Xu J. Zhang S. (2015). Mitogen-activated protein kinase cascades in signalling plant growth and development. *Trends Plant Sci.* 20 56–64. 10.1016/j.tplants.2014.10.001 25457109

[B60] Yamada-Akiyama H. Akiyama Y. Ebina M. Xu Q. Tsuruta S. Yazaki J. (2009). Analysis of expressed sequence tags in apomictic *Guinea grass* (*Panicum maximum*). *J. Plant Physiol.* 166 750–761. 10.1016/j.jplph.2008.10.001 19046615

[B61] Zhang W. McElroy D. Wu R. (1991). Analysis of rice actl 5′ region activity in transgenic rice plants. *Plant Cell* 3 1155–1165. 1821763 10.1105/tpc.3.11.1155 PMC160082

[B62] Zuckerkandl E. Pauling L. (1965). “Evolutionary divergence and convergence in proteins,” in *Evolving Genes and Proteins*, eds Bryson V. Vogel H. J. (New York, NY: Academic Press), 97–166.

