# Supplementary material for: The MAP3K-Coding QUI-GON JINN (QGJ) Gene Is Essential to the Formation of Unreduced Embryo Sacs in Paspalum
Source: Front Plant Sci. 2018 Oct 24;9:1547. doi: 10.3389/fpls.2018.01547 (PMC6207905; doi:10.3389/fpls.2018.01547)
Supplement: Supplementary file 1 [file Table_1.DOCX]

Supplementary Material

The MAP3K-coding *QUI-GON JINN* (*QGJ*) Gene Is Essential To The Formation of Unreduced Embryo Sacs In *Paspalum*

**Micaela Mancini, Hugo Permingeat, Carolina Colono, Lorena Siena, Fulvio Pupilli, Celeste Azzaro, Diva Maria de Alencar Dusi, Vera Tavares de Campos Carneiro, Maricel Podio, José Guillermo Seijo, Ana María González, Silvina A. Felitti, Juan Pablo A. Ortiz, Olivier Leblanc, Silvina C. Pessino^*^**

*** Correspondence:** Corresponding Author: pessino@arnet.com.ar

# Supplementary Figures and Tables

## Supplementary Figures

**Supplementary Fig. S1**

N46sex47 -GTCGTGTaCGCCTCTCTCTCTCCACACAGCTCGCTCTCTCTCCCTCTCTACACCTCATC 59

N46apo83 GTGTACGCCTCTCTCTCTCTCTCCACACAGCTCGCTCTCTCTCCCTcTcTACACCTCATC 60

N46apo85 GTGTACGCCTCTCTCTCTCTCTCCACACAGCTCGCTCTCTCTCCCTcTcTACACCTCATC 60

N46apo84 ----------------------------------CCCTCTCTCCCTCTCTACACCTCATC 26

N46apo86 ----------------------------------CCCTCTCTCCCTCTCTACACCTCATC 26

* ************************

N46sex47 TCTCTCGCTCGCAACCGTGGAGCGGCAGGATGCCGGCGTGGTGGAAGGGCAAGGGCAGGA 119

N46apo83 TCTCTCGCTCGCAACCGTGGAGCGGCAGGATGCCGGCGTGGTGGAAGGGCAAGGGCAGGA 120

N46apo85 TCTCTCGCTCGCAACCGTGGAGCGGCAGGATGCCGGCGTGGTGGAAGGGCAAGGGCAGGA 120

N46apo84 TCTCTCGCTCGCAGCCGTGGAGCGGCAGGATGCCGGCGTGGTGGAAGGGCAAGGGCAGGA 86

N46apo86 TCTCTCGCTCGCAGCCGTGGAGCGGCAGGATGCCGGCGTGGTGGAAGGGCAAGGGCAGGA 86

************* **********************************************

N46sex47 GCAAGTCTAGCAAGGCCGGGGCGGCGGCGCCGGTGgACTCAATCCCGGCGCCCGCA---- 175

N46apo83 GCAAGTCTAGCAAGGCCGGGGCGGCGGCGCCGGTGGACTCAATCCCGGCGCCCGCA---- 176

N46apo85 GCAAGTCTAGCAAGGCCGGGGCGGCGGCGCCGGTGGACTCAATCCCGGCGCCCGCA---- 176

N46apo84 GCAAGTCTAGCAAGGCCGGGGCGGCGGCGCCGGTGGACTCAATCCCGGCCCccGCCGCCG 146

N46apo86 GCAAGTCTAGCAAGGCCGGGGCGGCGGCGCCGGTGGACTCAATCCCGGCCCccGCCGCCG 146

************************************************* *****

N46sex47 -----GCCGCCGGGGAGGTTAAGGAGGCGAAGGGGAAGAAGAAGGCGAGCAGCTTCGACG 230

N46apo83 -----GCCGCCGGGGAGGTTAAGGAGGCGAAGGGGAAGAAGAAGGCgAGCAGCTTCGACG 231

N46apo85 -----GCCGCCGGGGAGGTTAAGGAGGCGAAGGGGAAGAAGAAGGCgAGCAGCTTCGACG 231

N46apo84 CCGCCGCCGCCGGGGAGGTTAAGGAGGCGAAGGGgAAGAAGAAGGCGAGCAGCTTCGACG 206

N46apo86 CCGCCGCCGCCGGGGAGGTTAAGGAGGCGAAGGGgAAGAAGAAGGCGAGCAGCTTCGACG 206

*******************************************************

N46sex47 AGGCGCTGCTCGCCAAGGGAGTCCGCGGGAAGCAGCAGCAGCAGCaGCCGCCGGCGGCgG 290

N46apo83 AGGCGCtGCTCGCCAAGGGAGTCCGCGGGAAGCAGCAGCAGCAG---CCGCCGGCaGCGG 288

N46apo85 AGGCGCtGCTCGCCAAGGGAGTCCGCGGGAAGCAGCAGCAGCAG---CCGCCGGCaGCGG 288

N46apo84 AGGCGCTGCTCGCCAAGGGAGTCCGCGGGAAGCAGCAGCAGCAG---CCGCCGGCGGCGG 263

N46apo86 AGGCGCTGCTCGCCAAGGGAGTCCGCGGGAAGCAGCAGCAGCAG---CCGCCGGCGGCGG 263

******************************************** ******** ****

N46sex47 CCGCcGctGTTGTCGGcCTCCCGCTCCCGCGGCCGGCGTCCTTGCCGGCGCCGCTGCCGT 350

N46apo83 CCGCCGCTGTTGTCGGCCTCCCGCTCCCGCGGCCGGCGTCCTTGCCGGCGCCGCTGCCGT 348

N46apo85 CCGCCGCTGTTGTCGGCCTCCCGCTCCCGCGGCCGGCGTCCTTGCCGGCGCCGCTGCCGT 348

N46apo84 CTGCCTCGGTTGTCGGGCTCCCGCTCCCGCGGCCGGCGTCCTTGCCGGCGCCGCTGCCGT 323

N46apo86 CTGCCTCGGTTGTCGGGCTCCCGCTCCCGCGGCCGGCGTCCTTGCCGGCGCCGCTGCCGT 323

* *** * ******** *******************************************

N46sex47 CTGCGTCCGCCTCGGCATCCGCCTCGGGGTCCAGCGGCGGCGGATCCTCGCTGGTGTCCT 410

N46apo83 CTGCGTCCGCCTCGGCATCCGCCTCGGGGTCCAGCGGCGGCGGATCCTCGCTGGTGTCCT 408

N46apo85 CTGCGTCCGCCTCGGCATCCGCCTCGGGGTCCAGCGGCGGCGGATCCTCGCTGGTGTCCT 408

N46apo84 CTGCGTCCGCCTCGGCATCCGCCTCGGGGTCCAGCGGCGGCGGATCCTCGCTGGTGTCCT 383

N46apo86 CTGCGTCCGCCTCGGCATCCGCCTCGGGGTCCAGCGGCGGCGGATCCTCGCTGGTGTCCT 383

************************************************************

N46sex47 CGGCGGCGTCCGACGAGCAGCTGGATTTCGGTGTTTACAGGTTGTCAGAAACAAGCAGCA 470

N46apo83 CGGCGGCGTCCGACGAGCAGCTGGATTTCGGTGTTTACAGGTTGTCAGAAACAAGCAGCA 468

N46apo85 CGGCGGCGTCCGACGAGCAGCTGGATTTCGGTGTTTACAGGTTGTCAGAAACAAGCAGCA 468

N46apo84 CGGCGGCGTCCGACGAGCAGCTGGATTTCGGTGTTTACAGGTTGTCAGAAACAAGCAGCA 443

N46apo86 CGGCGGCGTCCGACGAGCAGCTGGATTTCGGTGTTTACAGGTTGTCAGAAACAAGCAGCA 443

************************************************************

N46sex47 CACTTCCTGGCAGAACAGTACCAGCTGAATCTCGGAAACAAAGTCATGTGCTAGCAGAAG 530

N46apo83 CACTTCCTGGCAGAACAGTACCAGCTGAATCTCGGAAACAAAGTCATGTGCTAGCAGAAG 528

N46apo85 CACTTCCTGGCAGAACAGTACCAGCTGAATCTCGGAAACAAAGTCATGTGCTAGCAGAAG 528

N46apo84 CACTTCCTGGCAGAACAGTACCAGCTGAATCTCGGAAACAAAGTCATGTGCTAGCAGAAG 503

N46apo86 CACTTCCTGGCAGAACAGTACCAGCTGAATCTCGGAAACAAAGTCATGTGCTAGCAGAAG 503

************************************************************

N46sex47 GGCGCATTTTTACAAATAGTCAGGCTTTGGACCATTCCCGATTGTCTGAAACCTCAGTTT 590

N46apo83 GGCGCATTTTTACAAATAGTCAGGCTTTGGACCATTCCCGATTGTCTGAAACCTCAGTTT 588

N46apo85 GGCGCATTTTTACAAATAGTCAGGCTTTGGACCATTCCCGATTGTCTGAAACCTCAGTTT 588

N46apo84 GGCGCATTTTTACAAATAGTCAGGCTTTGGACCATTCCCGATTGTCTGAAACCTCAGTTT 563

N46apo86 GGCGCATTTTTACAAATAGTCAGGCTTTGGACCATTCCCGATTGTCTGAAACCTCAGTTT 563

************************************************************

N46sex47 CTCCAAGGAAAGAATTTCACCTTCAAAATCTGGATCTGGCAAATGATCAAACTAGGTACT 650

N46apo83 CTCCAAGGAAAGAATTTCACCTTCAAAATCTGGATCTGGCAAATGATCAAACTAGGTACT 648

N46apo85 CTCCAAGGAAAGAATTTCACCTTCAAAATCTGGATCTGGCAAATGATCAAACTAGGTACT 648

N46apo84 CTCCAAGGAAAGAATTTCACCTTCAAAATCTGGATCTGGCAAATGATCAAACTAGGTACT 623

N46apo86 CTCCAAGGAAAGAATTTCACCTTCAAAATCTGGATCTGGCAAATGATCAAACTAGGTACT 623

************************************************************

N46sex47 GTCGTGGTCGGAAATCAACAGAAATTGTGTTCAGTCCACAAGTGCCCGCTTCTCCACCTA 710

N46apo83 GTCGTGGTCGGAAATCAACAGAAATTGTGTTCAGTCCACAAGTGCCCGCTTCTCCACCTA 708

N46apo85 GTCGTGGTCGGAAATCAACAGAAATTGTGTTCAGTCCACAAGTGCCCGCTTCTCCACCTA 708

N46apo84 GTCGTGGTCGGAAATCAACAGAAATTGTGTTCAGTCCACAAGTGCCCGCTTCTCCACCTA 683

N46apo86 GTCGTGGTCGGAAATCAACAGAAATTGTGTTCAGTCCACAAGTGCCCGCTTCTCCACCTA 683

************************************************************

N46sex47 ATTCCAGAGGACATCACTATCCAACCTCCCCTGTGCCGACAAGAACATTTGGGCAAGGCC 770

N46apo83 ATTCCAGAGGACATCACTATCCAACCTCCCCTGTGCCGACAAGAACATTTGGGCAAGGCC 768

N46apo85 ATTCCAGAGGACATCACTATCCAACCTCCCCTGTGCCGACAAGAACATTTGGGCAAGGCC 768

N46apo84 ATTCCAGAGGACATCACTATCCAACCTCCCCTGTGCCGACAAGAACATTTGGGCAAGGCC 743

N46apo86 ATTCCAGAGGACATCACTATCCAACCTCCCCTGTGCCGACAAGAACATTTGGGCAAGGCC 743

************************************************************

N46sex47 CTGCATCTCCTACTTCATGGCAGGAgGATTCCCGAAGCTCAAGCTTACCTCAgCCTCTTC 830

N46apo83 CTGCATCTCCTACTTCATGGCAGGAgGATTCCCGAAGCTCAAGCTTACCTCAGCCTCTTC 828

N46apo85 CTGCATCTCCTACTTCATGGCAGGAgGATTCCCGAAGCTCAAGCTTACCTCAGCCTCTTC 828

N46apo84 CTGCATCTCCTACTTCATGGCAGGAgGATTCCCGAAGCTCAAGCTTACCTCAGCCTCTTC 803

N46apo86 CTGCATCTCCTACTTCATGGCAGGAgGATTCCCGAAGCTCAAGCTTACCTCAGCCTCTTC 803

************************************************************

N46sex47 CTCTTCCTCCAGGCTCCCCATGCTTACCTTCCCGCTCTCTACAGTGGAAAAAGGGGAAGT 890

N46apo83 CTCTTCCTCCAGGCTCCCCATGCTTACCTTCCCGCTCTCTACAGTGGAAAAAGGGGAAGT 888

N46apo85 CTCTTCCTCCAGGCTCCCCATGCTTACCTTCCCGCTCTCTACAGTGGAAAAAGGGGAAGT 888

N46apo84 CTCTTCCTCCAGGCTCCCCATGCTTACCTTCCCGCTCTCTACAGTGGAAAAAGGGGAAGT 863

N46apo86 CTCTTCCTCCAGGCTCCCCATGCTTACCTTCCCGCTCTCTACAGTGGAAAAAGGGGAAGT 863

************************************************************

N46sex47 TGCTTGGCAGTGGGACGTTTGGGCAAGTATATTTGGGATTCAACAGTGAAGGTGGTCAAA 950

N46apo83 TGCTTGGCAGTGGGACGTTTGGGCAAGTATATTTGGGATTCAACAGTGAAGGTGGTCAAA 948

N46apo85 TGCTTGGCAGTGGGACGTTTGGGCAAGTATATTTGGGATTCAACAGTGAAGGTGGTCAAA 948

N46apo84 TGCTTGGCAGTGGGACGTTTGGGCAAGTATATTTGGGATTCAACAGTGAAGGTGGTCAAA 923

N46apo86 TGCTTGGCAGTGGGACGTTTGGGCAAGTATATTTGGGATTCAACAGTGAAGGTGGTCAAA 923

************************************************************

N46sex47 TGTGTGCAATTAAAGAGGTTAAGGTCATTTCTGATGATTCTAACTCAAAAGAGTGCCTCA 1010

N46apo83 TGTGTGCAATTAAAGAGGTTAAGGTCATTTCTGATGATTCTAACTCAAAAGAGTGCCTCA 1008

N46apo85 TGTGTGCAATTAAAGAGGTTAAGGTCATTTCTGATGATTCTAACTCAAAAGAGTGCCTCA 1008

N46apo84 TGTGTGCAATTAAAGAGGTTAAGGTCATTTCTGATGATTCTAACTCAAAAGAGTGCCTCA 983

N46apo86 TGTGTGCAATTAAAGAGGTTAAGGTCATTTCTGATGATTCTAACTCAAAAGAGTGCCTCA 983

************************************************************

N46sex47 GGCAGCTAAATCAGGAAATCATGCTGCTGAGTCAGCTGTCACATCCAAACATTGTACAGT 1070

N46apo83 GGCAGCTAAATCAGGAAATCATGCTGCTGAGTCAGCTGTCACATCCAAACATTGTACAGT 1068

N46apo85 GGCAGCTAAATCAGGAAATCATGCTGCTGAGTCAGCTGTCACATCCAAACATTGTACAGT 1068

N46apo84 GGCAGCTAAATCAGGAAATCATGCTGCTGAGTCAGCTGTCACATCCAAACATTGTACAGT 1043

N46apo86 GGCAGCTAAATCAGGAAATCATGCTGCTGAGTCAGCTGTCACATCCAAACATTGTACAGT 1043

************************************************************

N46sex47 ACTATGGCAGTGATCTGTCTAATGAGACACTCTCGGTCTATCTCGAGTACGTTTCTGGGG 1130

N46apo83 ACTATGGCAGTGATCTGTCTAATGAGACACTCTCGGTCTATCTCGAGTACGTTTCTGGGG 1128

N46apo85 ACTATGGCAGTGATCTGTCTAATGAGACACTCTCGGTCTATCTCGAGTACGTTTCTGGGG 1128

N46apo84 ACTATGGCAGTGATCTGTCTAATGAGACACTCTCGGTCTATCTCGAGTACGTTTCTGGGG 1103

N46apo86 ACTATGGCAGTGATCTGTCTAATGAGACACTCTCGGTCTATCTCGAGTACGTTTCTGGGG 1103

************************************************************

N46sex47 GCTCCATCCATAAGTTGATTCAAGAATATGGTCCGTTTGGGGAGGCAGTTCTTCGGAATT 1190

N46apo83 GCTCCATCCATAAGTTGATTCAAGAATATGGTCCGTTTGGGGAGGCAGTTCTTCGGAATT 1188

N46apo85 GCTCCATCCATAAGTTGATTCAAGAATATGGTCCGTTTGGGGAGGCAGTTCTTCGGAATT 1188

N46apo84 GCTCCATCCATAAGTTGATTCAAGAATATGGTCCGTTTGGGGAGGCAGTTCTTCGGAATT 1163

N46apo86 GCTCCATCCATAAGTTGATTCAAGAATATGGTCCGTTTGGGGAGGCAGTTCTTCGGAATT 1163

************************************************************

N46sex47 ACACTGCGCAAATCCTTTCTGGTCTTGCATACTTGCATGGGCGGAATACAGTGCATAGGG 1250

N46apo83 ACACTGCGCAAATCCTTTCTGGTCTTGCATACTTGCATGGGCGGAATACAGTGCATAGGG 1248

N46apo85 ACACTGCGCAAATCCTTTCTGGTCTTGCATACTTGCATGGGCGGAATACAGTGCATAGGG 1248

N46apo84 ACACTGCGCAAATCCTTTCTGGTCTTGCATACTTGCATGGGCGGAATACAGTGCATAGGG 1223

N46apo86 ACACTGCGCAAATCCTTTCTGGTCTTGCATACTTGCATGGGCGGAATACAGTGCATAGGG 1223

************************************************************

N46sex47 ATATCAAAGGGGCAAACATACTTGTCGATCCTAATGGTGACATCAAGCTTGCTGATTTTG 1310

N46apo83 ATATCAAAGGGGCAAACATACTTGTCGATCCTAATGGTGACATCAAGCTTGCTGATTTTG 1308

N46apo85 ATATCAAAGGGGCAAACATACTTGTCGATCCTAATGGTGACATCAAGCTTGCTGATTTTG 1308

N46apo84 ATATCAAAGGGGCAAACATACTTGTCGATCCTAATGGTGACATCAAGCTTGCTGATTTTG 1283

N46apo86 ATATCAAAGGGGCAAACATACTTGTCGATCCTAATGGTGACATCAAGCTTGCTGATTTTG 1283

************************************************************

N46sex47 GCATGGCCAAGCATATATCAGCATACACATCTATCAGATCCTTCAAAGGGAGCCCTTACT 1370

N46apo83 GCATGGCCAAGCATATATCAGCATACACATCTATCAGATCCTTCAAAGGGAGCCCTTACT 1368

N46apo85 GCATGGCCAAGCATATATCAGCATACACATCTATCAGATCCTTCAAAGGGAGCCCTTACT 1368

N46apo84 GCATGGCCAAGCATATATCAGCATACACATCTATCAGATCCTTCAAAGGGAGCCCTTACT 1343

N46apo86 GCATGGCCAAGCATATATCAGCATACACATCTATCAGATCCTTCAAAGGGAGCCCTTACT 1343

************************************************************

N46sex47 GGATGGCACCAGAGGTTATCATGAATAGCAATGGTTACAGCCTTTCAGTAGACATTTGGA 1430

N46apo83 GGATGGCACCAGAGGTTATCATGAATAGCAATGGTTACAGCCTTTCAGTAGACATTTGGA 1428

N46apo85 GGATGGCACCAGAGGTTATCATGAATAGCAATGGTTACAGCCTTTCAGTAGACATTTGGA 1428

N46apo84 GGATGGCACCAGAGGTTATCATGAATAGCAATGGTTACAGCCTTTCAGTAGACATTTGGA 1403

N46apo86 GGATGGCACCAGAGGTTATCATGAATAGCAATGGTTACAGCCTTTCAGTAGACATTTGGA 1403

************************************************************

N46sex47 GCCTTGGCTGCACCATTCTTGAGATGGCAACAGCAAAACCTCCTTGGAGTCAGTATGAAG 1490

N46apo83 GCCTTGGCTGCACCATTCTTGAGATGGCAACAGCAAAACCTCCTTGGAGTCAGTATGAAG 1488

N46apo85 GCCTTGGCTGCACCATTCTTGAGATGGCAACAGCAAAACCTCCTTGGAGTCAGTATGAAG 1488

N46apo84 GCCTTGGCTGCACCATTCTTGAGATGGCAACAGCAAAACCTCCTTGGAGTCAGTATGAAG 1463

N46apo86 GCCTTGGCTGCACCATTCTTGAGATGGCAACAGCAAAACCTCCTTGGAGTCAGTATGAAG 1463

************************************************************

N46sex47 GGGTGGCTGCAATATTTAAGATTGGAAACAGCAAAGACATACCTGATATCCCAGATCATC 1550

N46apo83 GGGTGGCTGCAATATTTAAGATTGGAAACAGCAAAGACATACCTGATATCCCAGATCATC 1548

N46apo85 GGGTGGCTGCAATATTTAAGATTGGAAACAGCAAAGACATACCTGATATCCCAGATCATC 1548

N46apo84 GGGTGGCTGCAATATTTAAGATTGGAAACAGCAAAGACATACCTGATATCCCAGATCATC 1523

N46apo86 GGGTGGCTGCAATATTTAAGATTGGAAACAGCAAAGACATACCTGATATCCCAGATCATC 1523

************************************************************

N46sex47 TTTCTCCTGAGGCGAAAAGCTTTCTTAAACTATGTTTGCAGCGTGATCCTGCTGCCCGGC 1610

N46apo83 TTTCTCCTGAGGCGAAAAGCTTTCTTAAACTATGTTTGCAGCGTGATCCTGCTGCCCGGC 1608

N46apo85 TTTCTCCTGAGGCGAAAAGCTTTCTTAAACTATGTTTGCAGCGTGATCCTGCTGCCCGGC 1608

N46apo84 TTTCTCCTGAGGCGAAAAGCTTTCTTAAACTATGTTTGCAGCGTGATCCTGCTGCCCGGC 1583

N46apo86 TTTCTCCTGAGGCGAAAAGCTTTCTTAAACTATGTTTGCAGCGTGATCCTGCTGCCCGGC 1583

************************************************************

N46sex47 CTACTGCTGCTCAGTTGATGGATCACCCTTTTGTCAAGGACCATGCTACAGTTAGGAGTT 1670

N46apo83 CTACTGCTGCTCAGTTGATGGATCACCCTTTTGTCAAGGACCATGCTACAGTTAGGAGTT 1668

N46apo85 CTACTGCTGCTCAGTTGATGGATCACCCTTTTGTCAAGGACCATGCTACAGTTAGGAGTT 1668

N46apo84 CTACTGCTGCTCAGTTGATGGATCACCCTTTTGTCAAGGACCATGCTACAGTTAGGAGTT 1643

N46apo86 CTACTGCTGCTCAGTTGATGGATCACCCTTTTGTCAAGGACCATGCTACAGTTAGGAGTT 1643

************************************************************

N46sex47 CCAGGTCCAGCACCCCAAGGGATATGTTTCCTACTTCAACTGATGGAAAAAACAGCATGG 1730

N46apo83 CCAGGTCCAGCACCCCAAGGGATATGTTTCCTACTTCAACTGATGGAAAAAACAGCATGG 1728

N46apo85 CCAGGTCCAGCACCCCAAGGGATATGTTTCCTACTTCAACTGATGGAAAAAACAGCATGG 1728

N46apo84 CCAGGTCCAGCACCCCAAGGGATATGTTTCCTACTTCAACTGATGGAAAAAACAGCATGG 1703

N46apo86 CCAGGTCCAGCACCCCAAGGGATATGTTTCCTACTTCAACTGATGGAAAAAACAGCATGG 1703

************************************************************

N46sex47 TGCAGTCAAGCATTGCAGTTTCATCATACAGAAGCTTATCTCCATTAAGAGATCCTGATG 1790

N46apo83 TGCAGTCAAGCATTGCAGTTTCATCATACAGAAGCTTATCTCCATTAAGAGATCCTGATG 1788

N46apo85 TGCAGTCAAGCATTGCAGTTTCATCATACAGAAGCTTATCTCCATTAAGAGATCCTGATG 1788

N46apo84 TGCAGTCAAGCATTGCAGTTTCATCATACAGAAGCTTATCTCCATTAAGAGATCCTGATG 1763

N46apo86 TGCAGTCAAGCATTGCAGTTTCATCATACAGAAGCTTATCTCCATTAAGAGATCCTGATG 1763

************************************************************

N46sex47 TCGTGATAAGAAATTTGCAAGGACCAACATCCCCCATTCCTCCGATGTCAAATCGCAGGA 1850

N46apo83 TCGTGATAAGAAATTTGCAAGGACCAACATCCCCCATTCCTCCGATGTCAAATCGCAGGA 1848

N46apo85 TCGTGATAAGAAATTTGCAAGGACCAACATCCCCCATTCCTCCGATGTCAAATCGCAGGA 1848

N46apo84 TCGTGATAAGAAATTTGCAAGGACCAACATCCCCCATTCCTCCGATGTCAAATCGCAGGA 1823

N46apo86 TCGTGATAAGAAATTTGCAAGGACCAACATCCCCCATTCCTCCGATGTCAAATCGCAGGA 1823

************************************************************

N46sex47 TTGCGGCAA--------------------------------------------------- 1859

N46apo83 TTGCGGCAATGTATGTTATCCTTTTGTTAGACTTTACAAATTACATCTCATCAATTGTCC 1908

N46apo85 TTGCGGCAA--------------------------------------------------- 1857

N46apo84 TTGCGGCAATGTATGTTATCCTTTTGTTAGACTTTACAAATTACATCTCATCAATTGTCC 1883

N46apo86 TTGCGGCAA--------------------------------------------------- 1832

*********

N46sex47 -------------------------TCAACCCATCCAATATTCGGATGAACATGTCCGTG 1894

N46apo83 ACCTGATATATCcTTTTTtCTTCCAGCAACCCATCCAATATTCGGATGAACATGTCCGTG 1968

N46apo85 -------------------------TCAACCCATCCAATATTCGGATGAACATGTCCGTG 1892

N46apo84 ACCTGATATATCcTTTTTtCTTCCAGCAACCCATCCAATATTCGGATGAACATGTCCGTG 1943

N46apo86 -------------------------TCAACCCATCCAATATTCGGATGAACATGTCCGTG 1867

**********************************

N46sex47 CCTGTCTCTCCCTGCTCTAGCCCACTACGGCAGTATAGGCAGTCGAATCGAAGTTGCTTG 1954

N46apo83 CCTGTCTCTCCCTGCTCTAGCCCACTACGGCAGTATAGGCAGTCGAATCGAAGTTGCTTG 2028

N46apo85 CCTGTCTCTCCCTGCTCTAGCCCACTACGGCAGTATAGGCAGTCGAATCGAAGTTGCTTG 1952

N46apo84 CCTGTCTCTCCCTGCTCTAGCCCACTACGGCAGTATAGGCAGTCGAATCGAAGTTGCTTG 2003

N46apo86 CCTGTCTCTCCCTGCTCTAGCCCACTACGGCAGTATAGGCAGTCGAATCGAAGTTGCTTG 1927

************************************************************

N46sex47 CCATCGCCTCCTCATCCAGCCTATTCAGCTGGAGCAGCCAACTACAGTCCTATCAATAAC 2014

N46apo83 CCATCGCCTCCTCATCCAGCCTATTCAGCTGGAGCAGCCAACTACAGTCCTATCAATAAC 2088

N46apo85 CCATCGCCTCCTCATCCAGCCTATTCAGCTGGAGCAGCCAACTACAGTCCTATCAATAAC 2012

N46apo84 CCATCGCCTCCTCATCCAGCCTATTCAGCTGGAGCAGCCAACTACAGTCCTATCAATAAC 2063

N46apo86 CCATCGCCTCCTCATCCAGCCTATTCAGCTGGAGCAGCCAACTACAGTCCTATCAATAAC 1987

************************************************************

N46sex47 ACACTCTATCCGATGCGACCAAGCAGCGGTCTAACAGAGCCATGGCTCGAAAACTTTCAa 2074

N46apo83 ACACTCTATCCGATGCGACCAAGCAGCGGTCTAACAGAGCCATGGCTCGAAAACTTTCaa 2148

N46apo85 ACACTCTATCCGATGCGACCAAGCAGCGGTCTAACAGAGCCATGGCTCGAAAACTTTCaa 2072

N46apo84 ACACTCTATCCGATGCGACCAAGCAGCGGTCTAACAGAGCCATGGCTCGAAAACTTTCaa 2123

N46apo86 ACACTCTATCCGATGCGACCAAGCAGCGGTCTAACAGAGCCATGGCTCGAAAACTTTCaa 2047

************************************************************

N46sex47 CTGAAAACACAAACTTTTGATTCTCCAAGAAGATTATAGAGATTCCCAAAAAGAAGTAAT 2134

N46apo83 CTGAAAACACAAACTTTTGATTCTCCAAGAAGATTATAGAGATTCCCAAAAAGAAGTAAT 2208

N46apo85 CTGAAAACACAAACTTTTGATTCTCCAAGAAGATTATAGAGATTCCCAAAAAGAAGTAAT 2132

N46apo84 CTGAAAACACAAACTTTTGATTCTCCAAGAAGATTATAGAGATTCCCAAAAAGAAGTAAT 2183

N46apo86 CTGAAAACACAAACTTTTGATTCTCCAAGAAGATTATAGAGATTCCCAAAAAGAAGTAAT 2107

************************************************************

N46sex47 ACATTGTATAGAGGAAGTGTTCCATAATTTATGTTATTAGAAGAACAAAGGAACTGCCTC 2194

N46apo83 ACATTGTATAGAGGAAGTGTTCCATAATTTATGTTATTAGAAGAACAAAGGAACTGCCTC 2268

N46apo85 ACATTGTATAGAGGAAGTGTTCCATAATTTATGTTATTAGAAGAACAAAGGAACTGCCTC 2192

N46apo84 ACATTGTATAGAGGAAGTGTTCCATAATTTATGTTATTAGAAGAACAAAGGAACTGCCTC 2243

N46apo86 ACATTGTATAGAGGAAGTGTTCCATAATTTATGTTATTAGAAGAACAAAGGAACTGCCTC 2167

************************************************************

N46sex47 TTTTTtGTTTCACCCTTTCTGTATGTATTTTTCacAcGACGGGAGtGGTCGGCgttcAAG 2254

N46apo83 TTTTTtGTTTCACCCTTTCTGTATGTATTTTtCACCCAGGATGCAGACGCATCGCCTTTG 2328

N46apo85 TTTTTtGTTTCACCCTTTCTGTATGTATTTTtCACCCAGGATGCAGACGCATCGCCTTTG 2252

N46apo84 TTTTTtGTTTCACCCTTTCTGTATGTATTTTtCACCCAGGATGCAGACGCATCGCCTTTG 2303

N46apo86 TTTTTtGTTTCACCCTTTCTGTATGTATTTTtCACCCAGGATGCAGACGCATCGCCTTTG 2227

*********************************** * * * ** *

N46sex47 GCACAC------------------------------------------------------ 2260

N46apo83 TATAAATTCAGAGAAGAGCTAGTGAAAAAGAGTAGCATTTCACaTCgCCTTtGTCcATAT 2388

N46apo85 TATAAATTCAGAGAAGAGCTAGTGAAAAAGAGTAGCATTTCACaTCgCCTTtGTCcATAT 2312

N46apo84 TATAAATTCAGAGAAGAGCTAGTGAAAAAGAGTAGCATTTCACaTCgCCTTtGTCcATAT 2363

N46apo86 TATAAATTCAGAGAAGAGCTAGTGAAAAAGAGTAGCATTTCACaTCgCCTTtGTCcATAT 2287

*

N46sex47 ------------------------------------------------------------ 2260

N46apo83 TCTGGaTTTTTCTCAAGATGTGATTCtGAAAGGAGATTCATGGTAaTTGGATCTGatGcA 2448

N46apo85 TCTGGaTTTTTCTCAAGATGTGATTCtGAAAGGAGATTCATGGTAaTTGGATCTGatGcA 2372

N46apo84 TCTGGaTTTTTCTCAAGATGTGATTCtGAAAGGAGATTCATGGTAaTTGGATCTGatGcA 2423

N46apo86 TCTGGaTTTTTCTCAAGATGTGATTCtGAAAGGAGATTCATGGTAaTTGGATCTgatGcA 2347

N46sex47 ----- 2260

N46apo83 CATGG 2453

N46apo85 CATGG 2377

N46apo84 CATGG 2428

N46apo86 CATGG 2352

**Supplementary Fig. S1. Alignment of the N46 full nucleotide sequences retrieved from the floral transcriptome of apomictic and sexual *P. notatum* plants.** N46sex47: sexisotig 08547 (GFNR01008571.1). N46apo83: apoisotig 03083 (GFMI02003139.1). N46apo84: apoisotig 03084 (GFMI02003140.1). N46apo85: apoisotig 03085 (GFMI02003141.1). N46apo86: apoisotig 03086 (GFMI02003142.1). Isotigs 03083 and 03084 show a 76 nucleotide insertion between positions 1857-1934 and 1833-1908, respectively. The sequences of the primers used to amplify the intron region (FIP upper and FIP lower, see Materials and Methods, PCR amplification section) were marked in red.

**Supplementary Fig. S2**


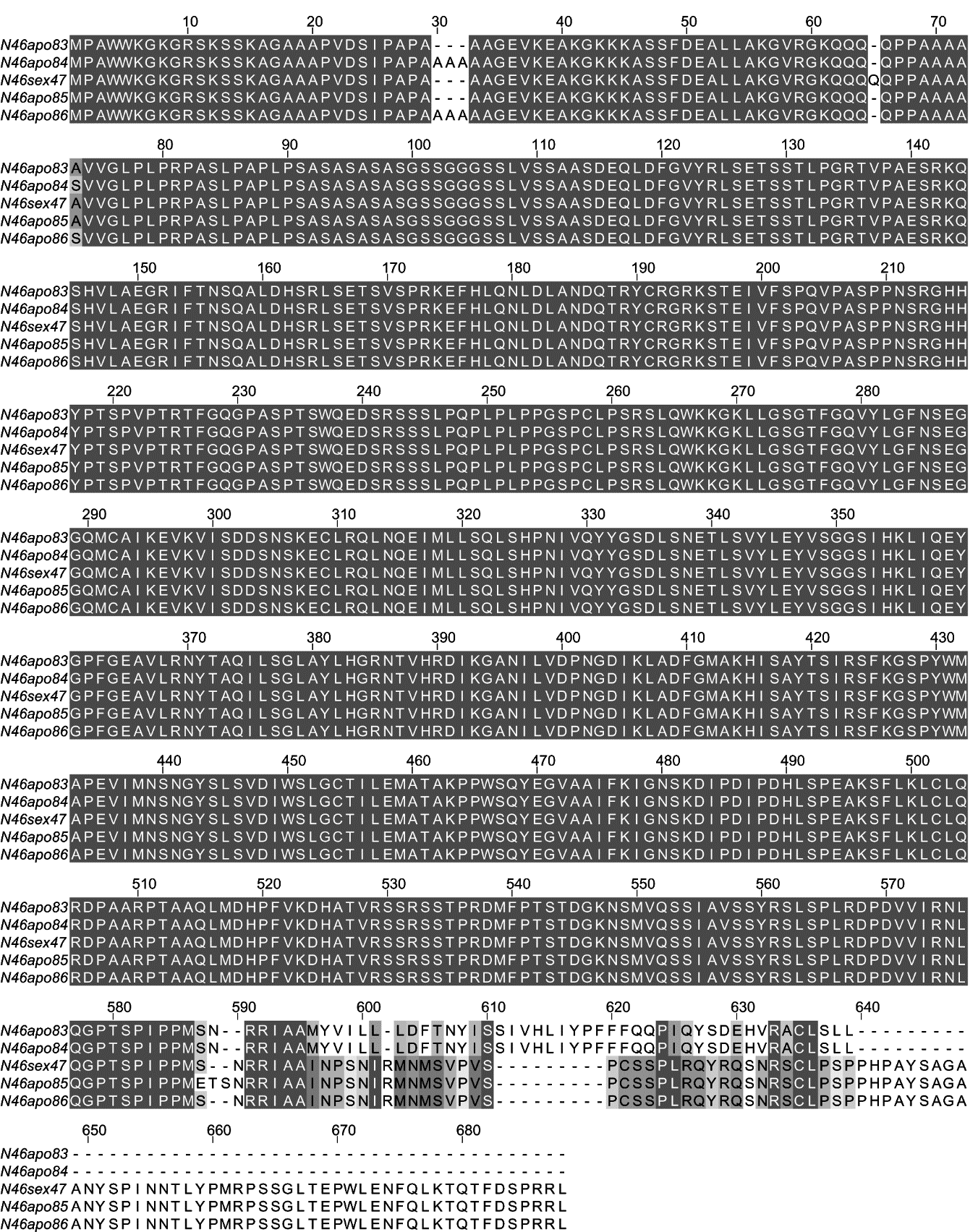


**Supplementary Fig. S2. Alignment of the N46 full protein sequences derived from the floral transcriptome of apomictic and sexual *P. notatum* plants.** N46sex47: sexisotig 08547 (GFNR01008571.1). N46apo83: apoisotig 03083 (GFMI02003139.1). N46apo84: apoisotig 03084 (GFMI02003140.1). N46apo85: apoisotig 03085 (GFMI02003141.1) N46apo86: apoisotig 03086 (GFMI02003142.1). Isotigs 03083 and 03084 generate proteins with a variable C-terminal end.

**
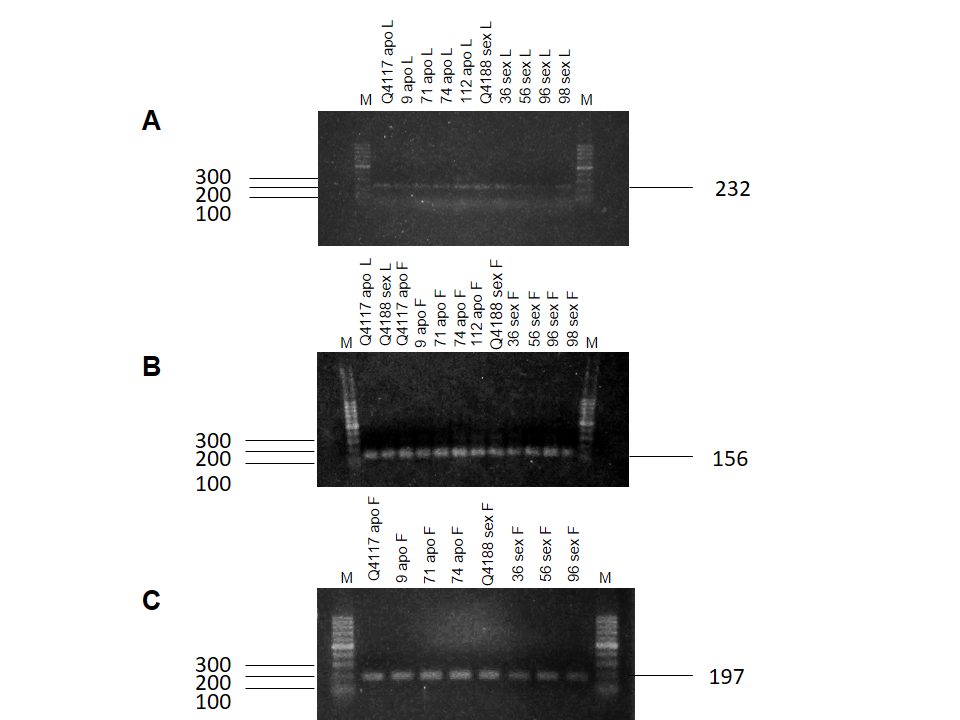
Supplementary Fig. S3**

**Supplementary Fig. S3.** **Genomic amplification of the *QGJ* 76-nt intron and cDNA amplification of different *QGJ* splice variants in apomictic and sexual plants.** A: amplifications from leaf (L) genomic samples using primers flanking the 76-nt intron produced bands of a size compatible with the presence of the intron from all apomictic and sexual plants. B: Detection of processed transcripts in all apomictic and sexual flower (F) and leaf (L) samples using intron-flanking primers. A faint upper band might correspond to the unprocessed form of the transcript (outcompeted during amplification by the shorter processed transcript). C: Detection of unprocessed transcript in flower samples using a primer pair with one of the oligonucleotides located inside the intron. Plants 9, 71, 74 and 112 are apomictic F_1_ hybrids derived from the cross Q4188 x Q4117. Plants 36, 56, 96 and 98 are sexual F_1_ hybrids derived from the cross Q4188 x Q4117. Fragment size is indicated in base pairs.

**Supplementary Fig. S4**


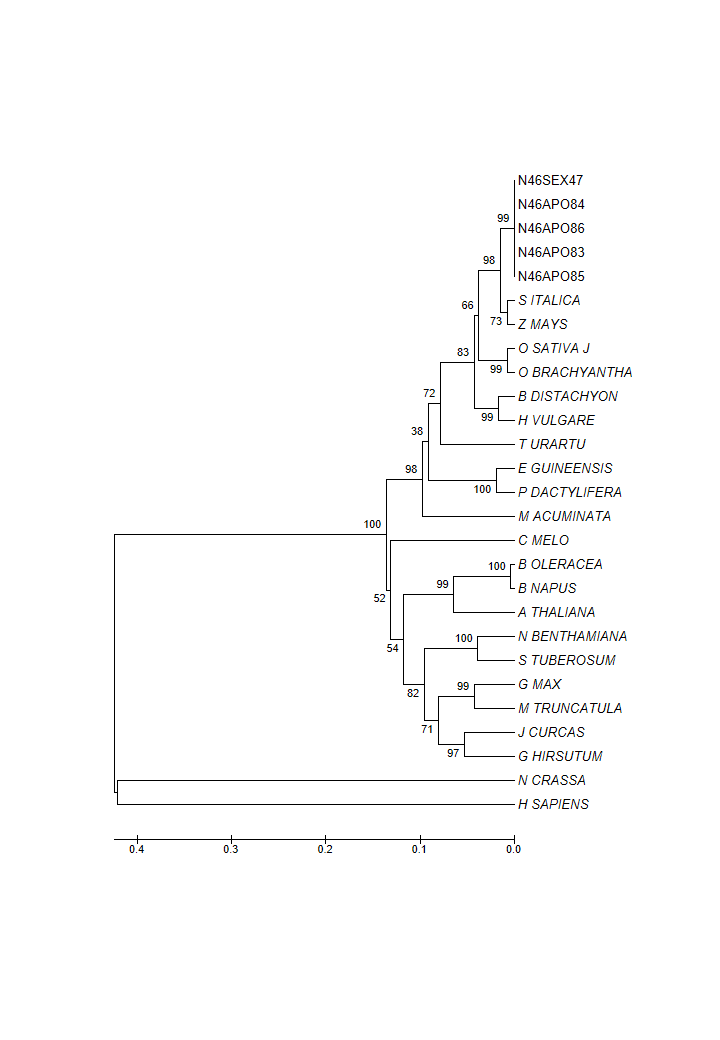


**Supplementary Fig. S4. Phylogeny tree constructed with the QGJ and QGJ-like protein sequences from *P. notatum* and 22 related/unrelated species**. The percentages of replicate trees in which the associated taxa clustered together in the bootstrap test (1000 replicates) are shown next to the branches. Accession used were: *Arabidopsis thaliana* [NP_564635]; *Brachypodium distachyon* [XP_003577728]*; Brassica napus* [NP_001302974.1]; *Brassica oleracea* [XP_013629876.1]; *Cucumis melo* [XP_008441055.1]; *Elaeis guineensis* [XP_010930645]; *Glycine max* [XP_003526863.1]*; Gossypium hirsutum* [ADI52619.1]; *Homo sapiens* [BC093674]; *Hordeum vulgare* [BAJ94063]; *Jatropha curcas* [XP_012066579.1]; *Medicago truncatula* [XP_003602611]; *Musa acuminata* [XP_009416942]; *Neurospora crassa* [NcAF034090]; *Nicotiana benthamiana* [AAS78639.1]; *Oryza brachyantha* [XP_006663285]*; Oryza sativa Japonica Group* [ABG22409.1]; *Phoenix dactylifera* [XP_008808477]*; Setaria italica* [XP_004979130]; *Solanum tuberosum* [XP_006350963.1]; *Triticum urartu* [EMS66799.1]; *Zea mays* [AGR03815.1].

**Supplementary Fig. S5**


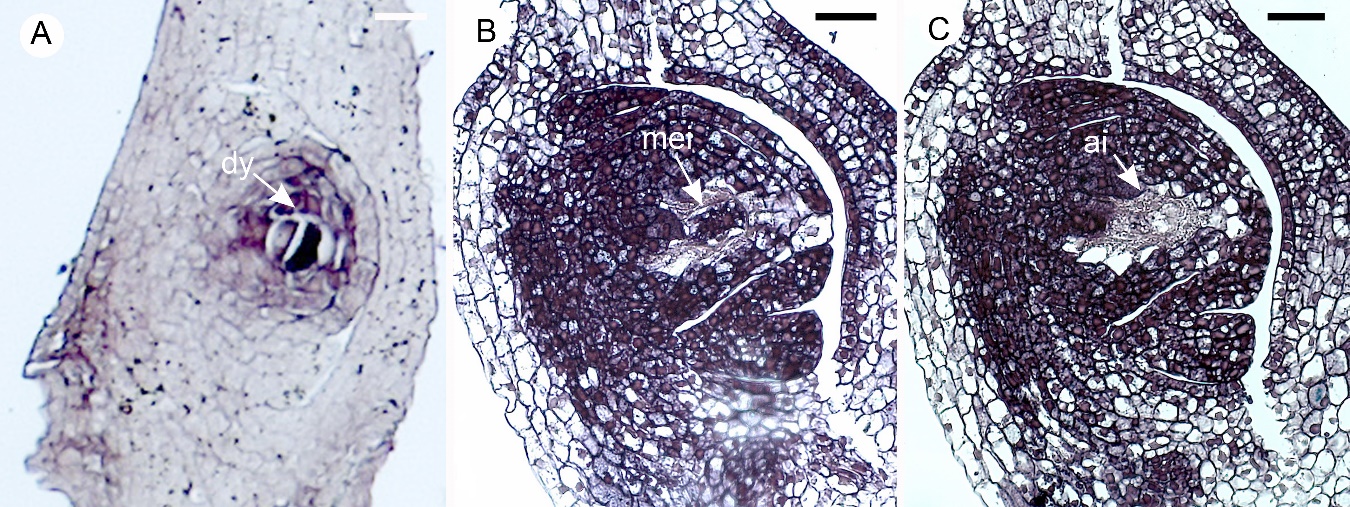


**Supplementary Fig. S5. Additional images of the *QGJ* *in situ* hybridization analyses on *Brachiaria brizantha* ovules.** Antisense probe reveals hybridization in the micropylar cells of dyads in the sexual genotype B105 (A) or in the nucellus and meiocytes of the apomictic genotype B30 (B-C). No hybridization is detected in the layer of proximal cells surrounding the meiocyte, from which the apospory initials originate (B-C). References: dy: dyads; mei: meiocyte; ai: apospory initials. Bars: 10 µm.

**Supplementary Fig. S6**

**
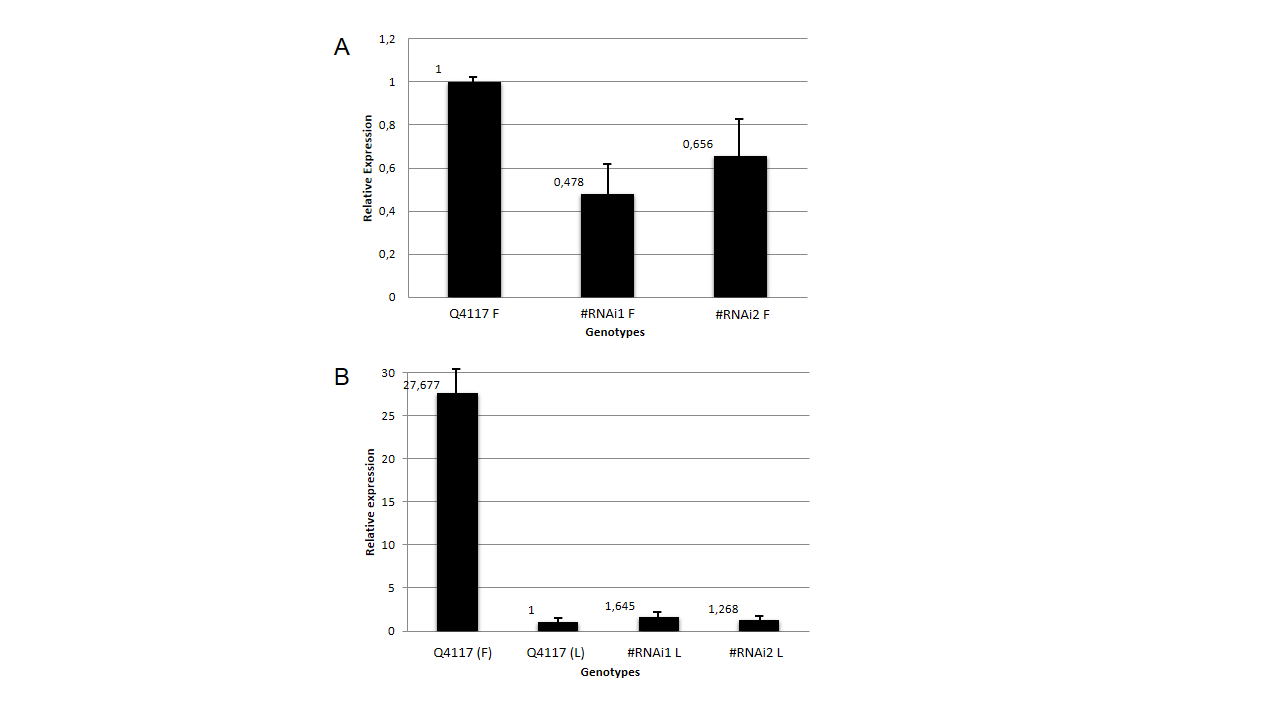
**

**Supplementary Fig. S6. Real-time PCR quantitation of *QUI-GON JINN* expression in wild type and transgenic *P. notatum* plants.** Relative expression values are indicated aside bars. Panel A: Expression in flowers (F, meiosis stage) from a wild-type control plant (apomictic genotype Q4117) and two RNAi lines (#RNAi1and #RNAi2). Panel B: Comparison of the expression in flowers (F, meiosis stage) of a control plant (apomictic genotype Q4117) and in leaves (L) of the same control plant and two RNAi lines (#RNAi1and #RNAi2).

**Supplementary Fig. S7**


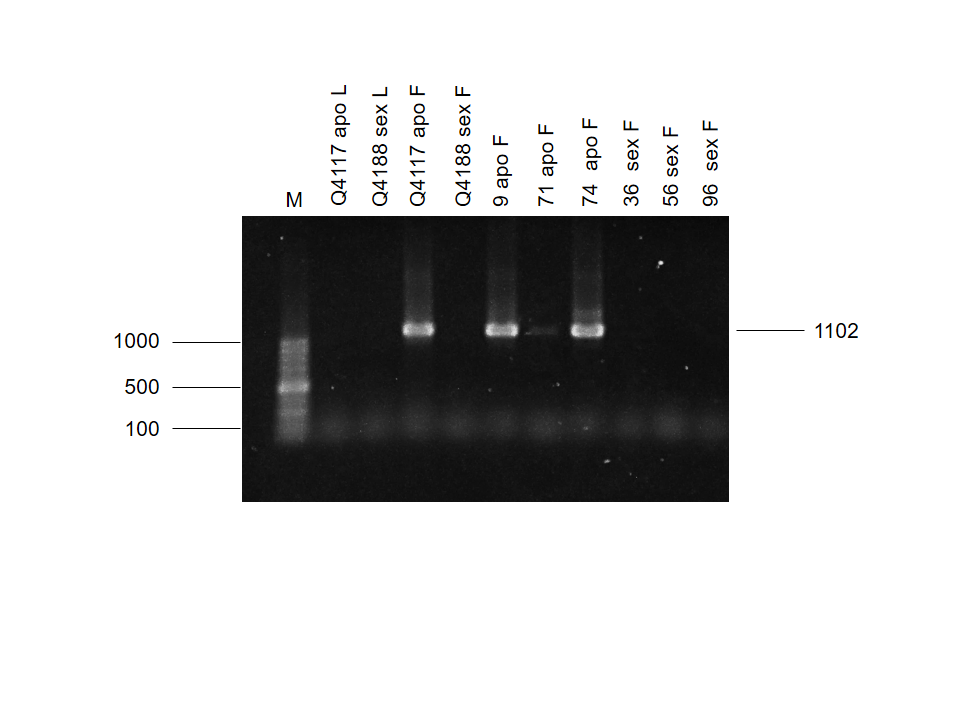


**Supplementary Fig. S7. Expression of *LNC_QGJ* in apomictic and sexual *P. notatum* plants.** Specific primers were used to amplify *PN_LNC-QGJ* from floral (F) or leaf (L) cDNA. Plants 9, 71 and 74 are apomictic F_1_ hybrids derived from the cross Q4188 x Q4117. Plants 36, 56 and 96 are sexual F_1_ hybrids derived from the cross Q4188 x Q4117. Fragment size is indicated in base pairs.


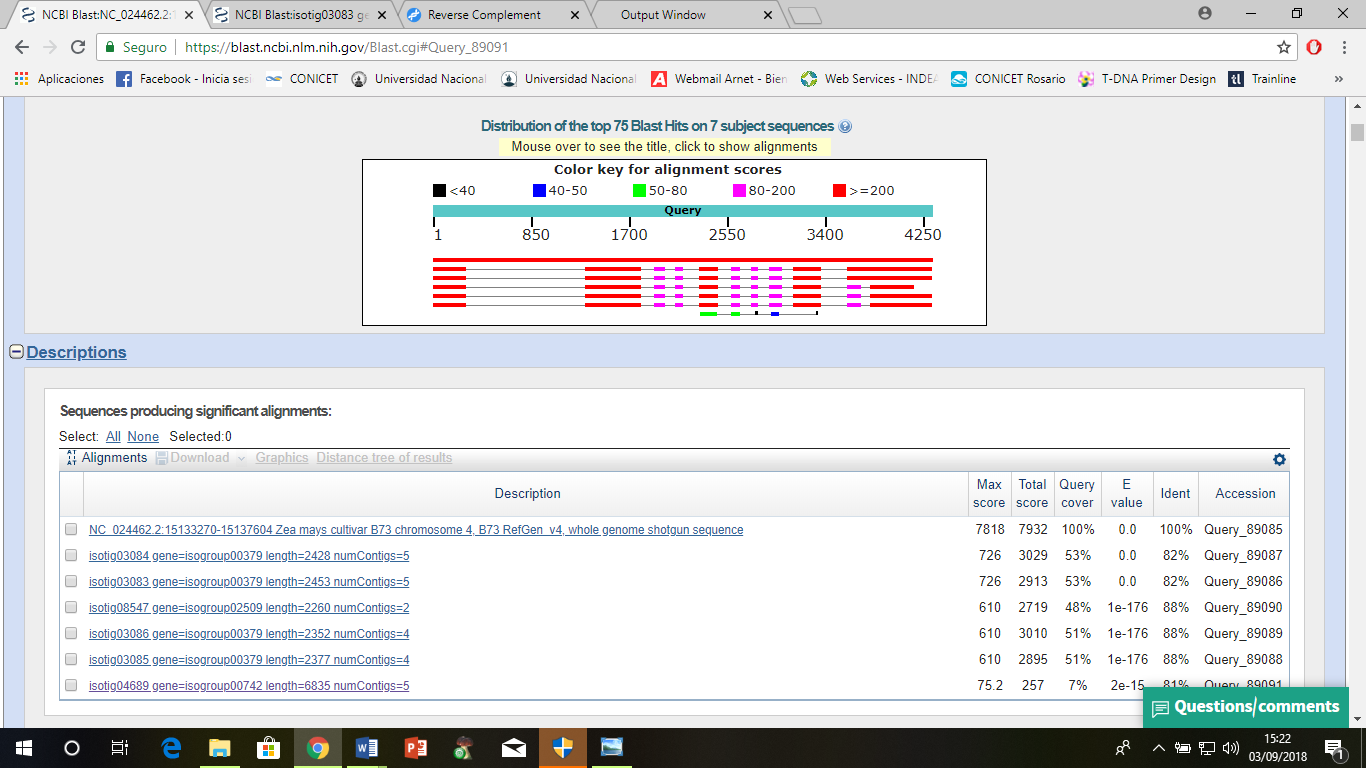
**Supplementary Fig. S8**

Query: GGRMZM6G513881 (NC_024462.2:15133270-15137604 Zea mays cultivar B73 chromosome 4, B73 RefGen_v4, whole genome shotgun sequence).

Subject: GGRMZM6G513881.

| lignment statistics for match #1 | | | | |
| --- | --- | --- | --- | --- |
| **Score** | **Expect** | **Identities** | **Gaps** | **Strand** |
| 7818 bits(8670) | 0.0 | 4335/4335(100%) | 0/4335(0%) | Plus/Plus |

Query 1 GCCGCTGCCATCGGCGATGTGAAGGAGGAGAAGGGGAAGAAGAAGGCGAGCAGCTTCGAC 60

||||||||||||||||||||||||||||||||||||||||||||||||||||||||||||

Sbjct 1 GCCGCTGCCATCGGCGATGTGAAGGAGGAGAAGGGGAAGAAGAAGGCGAGCAGCTTCGAC 60

Query 61 GAGGCGCTCCTCGCCAAGGGCGTCCGCGGGAAGCAGCAGCATGCGCCGGCGGCGGCGGGG 120

||||||||||||||||||||||||||||||||||||||||||||||||||||||||||||

Sbjct 61 GAGGCGCTCCTCGCCAAGGGCGTCCGCGGGAAGCAGCAGCATGCGCCGGCGGCGGCGGGG 120

Query 121 GAGGGTATGGGGCTCCCGCTCCCGCTCCCGCGCCCGGCGTCCTTGCCGACGCCGCTGCCG 180

||||||||||||||||||||||||||||||||||||||||||||||||||||||||||||

Sbjct 121 GAGGGTATGGGGCTCCCGCTCCCGCTCCCGCGCCCGGCGTCCTTGCCGACGCCGCTGCCG 180

Query 181 TCCGCGTCCGCGTCGGCCTCCGCCTCGGCGTCGGCGTCCAGCGGCGGCGACTCCTCGCTG 240

||||||||||||||||||||||||||||||||||||||||||||||||||||||||||||

Sbjct 181 TCCGCGTCCGCGTCGGCCTCCGCCTCGGCGTCGGCGTCCAGCGGCGGCGACTCCTCGCTG 240

Query 241 GGGTCCTCCACGTCCGACGACCAGCTGGATCTCGGGGTTTACAGGTGAGACATGGGGCAG 300

||||||||||||||||||||||||||||||||||||||||||||||||||||||||||||

Sbjct 241 GGGTCCTCCACGTCCGACGACCAGCTGGATCTCGGGGTTTACAGGTGAGACATGGGGCAG 300

Query 301 CGCACGGCGCGGCGGTTGGTCCACCACCGGTGGCTTCTTTGGGTGGGTGGGTGGTTGGTT 360

||||||||||||||||||||||||||||||||||||||||||||||||||||||||||||

Sbjct 301 CGCACGGCGCGGCGGTTGGTCCACCACCGGTGGCTTCTTTGGGTGGGTGGGTGGTTGGTT 360

Query 361 GGTTCACGGGGAAGCTAAAGTTGCGCCGTTTGGTGCTGTCCCCGTGCAGGATTTGGTGTA 420

||||||||||||||||||||||||||||||||||||||||||||||||||||||||||||

Sbjct 361 GGTTCACGGGGAAGCTAAAGTTGCGCCGTTTGGTGCTGTCCCCGTGCAGGATTTGGTGTA 420

Query 421 AAGCGCGCTCGttttttttGGGCCGCTAGTAATTAGCAGTTCCTGAACCTTATTATTATT 480

||||||||||||||||||||||||||||||||||||||||||||||||||||||||||||

Sbjct 421 AAGCGCGCTCGTTTTTTTTGGGCCGCTAGTAATTAGCAGTTCCTGAACCTTATTATTATT 480

Query 481 TTTGGCTGCTGAATCTCCTTGTGGATGGATTCGCTTGGTTGCCCGTCCGATTGAATTCAA 540

||||||||||||||||||||||||||||||||||||||||||||||||||||||||||||

Sbjct 481 TTTGGCTGCTGAATCTCCTTGTGGATGGATTCGCTTGGTTGCCCGTCCGATTGAATTCAA 540

Query 541 GGTGAACTGACGAGTGGGAGCCGTATGCTATATTCCCAAATGGGTTCGGGTGCCGAGATT 600

||||||||||||||||||||||||||||||||||||||||||||||||||||||||||||

Sbjct 541 GGTGAACTGACGAGTGGGAGCCGTATGCTATATTCCCAAATGGGTTCGGGTGCCGAGATT 600

Query 601 TGTTTAACTCTCTGTGTTTGAAATATTTGATGTTTGCTTGTTTGATTTTTATTGGGCGAC 660

||||||||||||||||||||||||||||||||||||||||||||||||||||||||||||

Sbjct 601 TGTTTAACTCTCTGTGTTTGAAATATTTGATGTTTGCTTGTTTGATTTTTATTGGGCGAC 660

Query 661 TTGGATTTTGGAGCTGCAATTTTCGATGTGTTATCTGCTAAATTTTGCATTCTCTGGGTA 720

||||||||||||||||||||||||||||||||||||||||||||||||||||||||||||

Sbjct 661 TTGGATTTTGGAGCTGCAATTTTCGATGTGTTATCTGCTAAATTTTGCATTCTCTGGGTA 720

Query 721 TGCTTTAGTGCCCAGTTTAGAGCGTTGACCTTTTGCCTTCTGAAATTTGTCCAACCTGTA 780

||||||||||||||||||||||||||||||||||||||||||||||||||||||||||||

Sbjct 721 TGCTTTAGTGCCCAGTTTAGAGCGTTGACCTTTTGCCTTCTGAAATTTGTCCAACCTGTA 780

Query 781 GACAAATAGTAACACGCTACTCTGAAATTCGGTCAGttttttttCTTCTAATGTACAAAT 840

||||||||||||||||||||||||||||||||||||||||||||||||||||||||||||

Sbjct 781 GACAAATAGTAACACGCTACTCTGAAATTCGGTCAGTTTTTTTTCTTCTAATGTACAAAT 840

Query 841 TCCATCAGTTCGGAGGATTTCTGTGGCTATTTGAATTTTGGGGGAATTGCTCCAATTGAT 900

||||||||||||||||||||||||||||||||||||||||||||||||||||||||||||

Sbjct 841 TCCATCAGTTCGGAGGATTTCTGTGGCTATTTGAATTTTGGGGGAATTGCTCCAATTGAT 900

Query 901 TTACGGATTCCATTATCCTATTACGACTTATACTATGCCTAAAACACTTCTGTTGATGAT 960

||||||||||||||||||||||||||||||||||||||||||||||||||||||||||||

Sbjct 901 TTACGGATTCCATTATCCTATTACGACTTATACTATGCCTAAAACACTTCTGTTGATGAT 960

Query 961 TGCTTCTGAAACGAGTTCATTCATCTTCTTACATTCGGGTGGACTTCTTTGATGGGCTCG 1020

||||||||||||||||||||||||||||||||||||||||||||||||||||||||||||

Sbjct 961 TGCTTCTGAAACGAGTTCATTCATCTTCTTACATTCGGGTGGACTTCTTTGATGGGCTCG 1020

Query 1021 GTTCTTCTAGTCTAATTACAGATATTAAAATATATATTTTGACACTTCGAAGCAATATTT 1080

||||||||||||||||||||||||||||||||||||||||||||||||||||||||||||

Sbjct 1021 GTTCTTCTAGTCTAATTACAGATATTAAAATATATATTTTGACACTTCGAAGCAATATTT 1080

Query 1081 AATCCAAAGATACCTCATTCATTGGAGACGTTTTTTGTTTGAGAAAAATATTCCCCCTTT 1140

||||||||||||||||||||||||||||||||||||||||||||||||||||||||||||

Sbjct 1081 AATCCAAAGATACCTCATTCATTGGAGACGTTTTTTGTTTGAGAAAAATATTCCCCCTTT 1140

Query 1141 TTCTGTATCCTTGCATGATTACAAAGTAGGAGGGAATAGTTGTCTAAGGAAATAATTGCG 1200

||||||||||||||||||||||||||||||||||||||||||||||||||||||||||||

Sbjct 1141 TTCTGTATCCTTGCATGATTACAAAGTAGGAGGGAATAGTTGTCTAAGGAAATAATTGCG 1200

Query 1201 CTTCGATAGTaaaaaaaaaTACTATTGATTGAGATACGACATCATGTAAAGGCAAAGTAT 1260

||||||||||||||||||||||||||||||||||||||||||||||||||||||||||||

Sbjct 1201 CTTCGATAGTAAAAAAAAATACTATTGATTGAGATACGACATCATGTAAAGGCAAAGTAT 1260

Query 1261 CACTATTTGTTTATCGAAGACATGCATTTCTGAGAGTTGTTTCCTATGTCCGATTTCATA 1320

||||||||||||||||||||||||||||||||||||||||||||||||||||||||||||

Sbjct 1261 CACTATTTGTTTATCGAAGACATGCATTTCTGAGAGTTGTTTCCTATGTCCGATTTCATA 1320

Query 1321 GGTTGTCAGAAACAAGCAGCACTCTTCTGGGCAGAACAGTAGCAATTGAATCCCGGAAAC 1380

||||||||||||||||||||||||||||||||||||||||||||||||||||||||||||

Sbjct 1321 GGTTGTCAGAAACAAGCAGCACTCTTCTGGGCAGAACAGTAGCAATTGAATCCCGGAAAC 1380

Query 1381 AAAGTCAGGTGCCAGCAGAGGGGACCATTTTCACCAATAATCAGGCTGTGGAGCATACCC 1440

||||||||||||||||||||||||||||||||||||||||||||||||||||||||||||

Sbjct 1381 AAAGTCAGGTGCCAGCAGAGGGGACCATTTTCACCAATAATCAGGCTGTGGAGCATACCC 1440

Query 1441 GGTTGTCTGAAACATCAGTTTCCCCAAGGAAAGAATTTCGCCCTCAAAATTTGGATCTTG 1500

||||||||||||||||||||||||||||||||||||||||||||||||||||||||||||

Sbjct 1441 GGTTGTCTGAAACATCAGTTTCCCCAAGGAAAGAATTTCGCCCTCAAAATTTGGATCTTG 1500

Query 1501 CAAATGATCGAACTACATACTGCCGTGGTCGGAGATCAACCGAAATCGTGTTCAGTACAC 1560

||||||||||||||||||||||||||||||||||||||||||||||||||||||||||||

Sbjct 1501 CAAATGATCGAACTACATACTGCCGTGGTCGGAGATCAACCGAAATCGTGTTCAGTACAC 1560

Query 1561 AAGTGCCCACTTCTCCTCCTAGTTCAAGAGGACATCACTATCAAAATTCGCCTGTGCCAT 1620

||||||||||||||||||||||||||||||||||||||||||||||||||||||||||||

Sbjct 1561 AAGTGCCCACTTCTCCTCCTAGTTCAAGAGGACATCACTATCAAAATTCGCCTGTGCCAT 1620

Query 1621 CAAGAACATTTGGGCAATGCCCTGCATCTCCTACTTCATGGCAGGATGATTCGCGAAGCT 1680

||||||||||||||||||||||||||||||||||||||||||||||||||||||||||||

Sbjct 1621 CAAGAACATTTGGGCAATGCCCTGCATCTCCTACTTCATGGCAGGATGATTCGCGAAGCT 1680

Query 1681 CAAGCTCACCCCAACCACTTCCTCTTCCTCCAGGTTCCCCATGCTTGCCTTCCTCTTCTC 1740

||||||||||||||||||||||||||||||||||||||||||||||||||||||||||||

Sbjct 1681 CAAGCTCACCCCAACCACTTCCTCTTCCTCCAGGTTCCCCATGCTTGCCTTCCTCTTCTC 1740

Query 1741 TACAGTGGAAGAAGGGGAAGTTGCTAGGTAGTGGGACGTTTGGGCAAGTATACATGGGAT 1800

||||||||||||||||||||||||||||||||||||||||||||||||||||||||||||

Sbjct 1741 TACAGTGGAAGAAGGGGAAGTTGCTAGGTAGTGGGACGTTTGGGCAAGTATACATGGGAT 1800

Query 1801 TCAACAGGTACTTGGCCTGATTTTGAAGCATTGTGTTTTTCTCACAtttttttGGATGAG 1860

||||||||||||||||||||||||||||||||||||||||||||||||||||||||||||

Sbjct 1801 TCAACAGGTACTTGGCCTGATTTTGAAGCATTGTGTTTTTCTCACATTTTTTTGGATGAG 1860

Query 1861 TTATTGTATTCGTTTCGTTTTAACACCATACAAACTCATATTAGAAATATATGCTATGCA 1920

||||||||||||||||||||||||||||||||||||||||||||||||||||||||||||

Sbjct 1861 TTATTGTATTCGTTTCGTTTTAACACCATACAAACTCATATTAGAAATATATGCTATGCA 1920

Query 1921 GTGAAGGTGGTCAAATGTGTGCAATTAAAGAGGTTAAGGTCATTTCAGATGATTCTAACT 1980

||||||||||||||||||||||||||||||||||||||||||||||||||||||||||||

Sbjct 1921 GTGAAGGTGGTCAAATGTGTGCAATTAAAGAGGTTAAGGTCATTTCAGATGATTCTAACT 1980

Query 1981 CAAAGGAGTCCCTCAGGCAGCTAAATCAGGTGATAATATACTACGGAAATATTGTAAATA 2040

||||||||||||||||||||||||||||||||||||||||||||||||||||||||||||

Sbjct 1981 CAAAGGAGTCCCTCAGGCAGCTAAATCAGGTGATAATATACTACGGAAATATTGTAAATA 2040

Query 2041 TTGGCATCAATGTTGTAGAAGAAACTGTATTGCTTTCTCGGACTGACACTAGTTGATATG 2100

||||||||||||||||||||||||||||||||||||||||||||||||||||||||||||

Sbjct 2041 TTGGCATCAATGTTGTAGAAGAAACTGTATTGCTTTCTCGGACTGACACTAGTTGATATG 2100

Query 2101 TACAGGAAATCGTGCTGCTGAGTCAGCTGTCACATCCAAACATTGTTCAGTACTATGGCA 2160

||||||||||||||||||||||||||||||||||||||||||||||||||||||||||||

Sbjct 2101 TACAGGAAATCGTGCTGCTGAGTCAGCTGTCACATCCAAACATTGTTCAGTACTATGGCA 2160

Query 2161 GTGATTTGGTAATTACTGAATTAACAGTGATCTTCTAGGATTACATAATAATCATTCATA 2220

||||||||||||||||||||||||||||||||||||||||||||||||||||||||||||

Sbjct 2161 GTGATTTGGTAATTACTGAATTAACAGTGATCTTCTAGGATTACATAATAATCATTCATA 2220

Query 2221 ACACTCATAAGCAGTGCAATTACAACCTTGAAATTAAGCATTGATACAATGGTCATATTA 2280

||||||||||||||||||||||||||||||||||||||||||||||||||||||||||||

Sbjct 2221 ACACTCATAAGCAGTGCAATTACAACCTTGAAATTAAGCATTGATACAATGGTCATATTA 2280

Query 2281 ATATGTGTGTTTTATGCTATAATGCAGTGCAATGAGACACTCTCGGTCTATCTCGAGTAT 2340

||||||||||||||||||||||||||||||||||||||||||||||||||||||||||||

Sbjct 2281 ATATGTGTGTTTTATGCTATAATGCAGTGCAATGAGACACTCTCGGTCTATCTCGAGTAT 2340

Query 2341 GTTTCTGGGGGCTCTATCCATAAGTTGCTTCAAGAATATGGTCCGTTTGGGGAGGCAGTG 2400

||||||||||||||||||||||||||||||||||||||||||||||||||||||||||||

Sbjct 2341 GTTTCTGGGGGCTCTATCCATAAGTTGCTTCAAGAATATGGTCCGTTTGGGGAGGCAGTG 2400

Query 2401 CTTCGGAATTACACAGCACAAATCCTTTCTGGCCTTGCATACTTGCATGGGCGGAATACA 2460

||||||||||||||||||||||||||||||||||||||||||||||||||||||||||||

Sbjct 2401 CTTCGGAATTACACAGCACAAATCCTTTCTGGCCTTGCATACTTGCATGGGCGGAATACA 2460

Query 2461 GTGCATAGGTATTTGGCAGTCAATACCATCATCAATATATAAGATACAATTGTCTTTTAT 2520

||||||||||||||||||||||||||||||||||||||||||||||||||||||||||||

Sbjct 2461 GTGCATAGGTATTTGGCAGTCAATACCATCATCAATATATAAGATACAATTGTCTTTTAT 2520

Query 2521 TCTTTCGTACCACATGCCAGTTTTAGCATCTTTACTTATTAACGGAAGTTTACTTATCTG 2580

||||||||||||||||||||||||||||||||||||||||||||||||||||||||||||

Sbjct 2521 TCTTTCGTACCACATGCCAGTTTTAGCATCTTTACTTATTAACGGAAGTTTACTTATCTG 2580

Query 2581 ACAGGGATATCAAAGGGGCAAACATACTTGTAGATCCTAATGGTGACATCAAGCTTGCTG 2640

||||||||||||||||||||||||||||||||||||||||||||||||||||||||||||

Sbjct 2581 ACAGGGATATCAAAGGGGCAAACATACTTGTAGATCCTAATGGTGACATCAAGCTTGCTG 2640

Query 2641 ATTTTGGTATGGCGAAACATGTAAGTGCTAAACTCTGTCAATCATCTCACAATTCAATAG 2700

||||||||||||||||||||||||||||||||||||||||||||||||||||||||||||

Sbjct 2641 ATTTTGGTATGGCGAAACATGTAAGTGCTAAACTCTGTCAATCATCTCACAATTCAATAG 2700

Query 2701 TTAGCTGCCTGGCTTTCTTATCTTATGATTTTAATCAATAATCTGTTATGTGTACACAGA 2760

||||||||||||||||||||||||||||||||||||||||||||||||||||||||||||

Sbjct 2701 TTAGCTGCCTGGCTTTCTTATCTTATGATTTTAATCAATAATCTGTTATGTGTACACAGA 2760

Query 2761 TATCAGCATATACATCTATCAAATCCTTCAAAGGGAGCCCTTACTGGATGGCACCAGAGG 2820

||||||||||||||||||||||||||||||||||||||||||||||||||||||||||||

Sbjct 2761 TATCAGCATATACATCTATCAAATCCTTCAAAGGGAGCCCTTACTGGATGGCACCAGAGG 2820

Query 2821 TGAGGCTACTGAAGTTGAAGTATCTTGTTGATTCCCCCTAGTGAACAGAAACTAAAAGGC 2880

||||||||||||||||||||||||||||||||||||||||||||||||||||||||||||

Sbjct 2821 TGAGGCTACTGAAGTTGAAGTATCTTGTTGATTCCCCCTAGTGAACAGAAACTAAAAGGC 2880

Query 2881 TTGAATTCTTTCTTTTCATGCCAAAATTCAGGTTATTATGAATAGCAATGGTTACAGCCT 2940

||||||||||||||||||||||||||||||||||||||||||||||||||||||||||||

Sbjct 2881 TTGAATTCTTTCTTTTCATGCCAAAATTCAGGTTATTATGAATAGCAATGGTTACAGCCT 2940

Query 2941 TTCAGTAGACATTTGGAGCCTTGGCTGCACCATTCTTGAGATGGCAACAGCAAAGCCTCC 3000

||||||||||||||||||||||||||||||||||||||||||||||||||||||||||||

Sbjct 2941 TTCAGTAGACATTTGGAGCCTTGGCTGCACCATTCTTGAGATGGCAACAGCAAAGCCTCC 3000

Query 3001 TTGGAGTCAGTATGAAGGGGTGAGTTATTGACTTAATGGATGCAATCATTTTTACATGCT 3060

||||||||||||||||||||||||||||||||||||||||||||||||||||||||||||

Sbjct 3001 TTGGAGTCAGTATGAAGGGGTGAGTTATTGACTTAATGGATGCAATCATTTTTACATGCT 3060

Query 3061 TATCAATTATGATGACTATTCTTCCATTGATCATCTGCTAATATTTGGATGGATTTCACA 3120

||||||||||||||||||||||||||||||||||||||||||||||||||||||||||||

Sbjct 3061 TATCAATTATGATGACTATTCTTCCATTGATCATCTGCTAATATTTGGATGGATTTCACA 3120

Query 3121 GGTGGCAGCAATATTCAAAATTGGCAACAGCAAAGACATACCAGATATCCCAAATAATCT 3180

||||||||||||||||||||||||||||||||||||||||||||||||||||||||||||

Sbjct 3121 GGTGGCAGCAATATTCAAAATTGGCAACAGCAAAGACATACCAGATATCCCAAATAATCT 3180

Query 3181 TTCTTCTGAGGCAAAAAGTTTCCTGAAACTCTGCTTGCAGCGTGATCCTGCTGCCCGCCC 3240

||||||||||||||||||||||||||||||||||||||||||||||||||||||||||||

Sbjct 3181 TTCTTCTGAGGCAAAAAGTTTCCTGAAACTCTGCTTGCAGCGTGATCCTGCTGCCCGCCC 3240

Query 3241 TACAGCTGCTCAGCTGATGGATCACCCTTTTGTCAAGGACCAGGCTACAGTCAGGAGTTC 3300

||||||||||||||||||||||||||||||||||||||||||||||||||||||||||||

Sbjct 3241 TACAGCTGCTCAGCTGATGGATCACCCTTTTGTCAAGGACCAGGCTACAGTCAGGAGTTC 3300

Query 3301 CAGGTCCAGTATCACAAGGGATATGTTTCCTAATTCAACTGACGGAAAAAACAGCAGGGT 3360

||||||||||||||||||||||||||||||||||||||||||||||||||||||||||||

Sbjct 3301 CAGGTCCAGTATCACAAGGGATATGTTTCCTAATTCAACTGACGGAAAAAACAGCAGGGT 3360

Query 3361 ACGTCATCCACATACTGCCTCCGATTTTGGGACTAATATTATCAAATAGATGCCCCTTTA 3420

||||||||||||||||||||||||||||||||||||||||||||||||||||||||||||

Sbjct 3361 ACGTCATCCACATACTGCCTCCGATTTTGGGACTAATATTATCAAATAGATGCCCCTTTA 3420

Query 3421 TGAAATCGGCTTGTGTTCTGTATTAGAGCATGATAGAAGATATGCATGTATATATATACA 3480

||||||||||||||||||||||||||||||||||||||||||||||||||||||||||||

Sbjct 3421 TGAAATCGGCTTGTGTTCTGTATTAGAGCATGATAGAAGATATGCATGTATATATATACA 3480

Query 3481 TGTAAAACTGTAAAGCTGATCACAATTTATTACGATTTGTCACACCGATCATATTCCAAG 3540

||||||||||||||||||||||||||||||||||||||||||||||||||||||||||||

Sbjct 3481 TGTAAAACTGTAAAGCTGATCACAATTTATTACGATTTGTCACACCGATCATATTCCAAG 3540

Query 3541 GTTCCATGTTCCTTCATGATTTCTTAATGGTGTGCTTTTTTCTCCAGGTGAAAATTGAAA 3600

||||||||||||||||||||||||||||||||||||||||||||||||||||||||||||

Sbjct 3541 GTTCCATGTTCCTTCATGATTTCTTAATGGTGTGCTTTTTTCTCCAGGTGAAAATTGAAA 3600

Query 3601 CTTCGTCATACAGAAGTTTATCTCCTTTAAGAGATCCTGATATCCTTGGAAGAAACTTGC 3660

||||||||||||||||||||||||||||||||||||||||||||||||||||||||||||

Sbjct 3601 CTTCGTCATACAGAAGTTTATCTCCTTTAAGAGATCCTGATATCCTTGGAAGAAACTTGC 3660

Query 3661 CAGGACCAACATCCCCTATTCCTTCGACATCAAGTCGCAGGATCGCAGCATTGTATGTTC 3720

||||||||||||||||||||||||||||||||||||||||||||||||||||||||||||

Sbjct 3661 CAGGACCAACATCCCCTATTCCTTCGACATCAAGTCGCAGGATCGCAGCATTGTATGTTC 3720

Query 3721 TCCTTCTGTCAGATTTATATCAATTTACATATTGTGAACTGTGTCTCAACCGATCTCTCT 3780

||||||||||||||||||||||||||||||||||||||||||||||||||||||||||||

Sbjct 3721 TCCTTCTGTCAGATTTATATCAATTTACATATTGTGAACTGTGTCTCAACCGATCTCTCT 3780

Query 3781 TTCGTCCAGGAACACATCCAATGTTCGGATGAACATGTCGCTGCCTGTCTCTCCCTGCTC 3840

||||||||||||||||||||||||||||||||||||||||||||||||||||||||||||

Sbjct 3781 TTCGTCCAGGAACACATCCAATGTTCGGATGAACATGTCGCTGCCTGTCTCTCCCTGCTC 3840

Query 3841 TAGCCCGCTACGGCAGTACAGGCAGTCCAACCGAAGTTGCTTGCGCTCCCCTCCCCACCC 3900

||||||||||||||||||||||||||||||||||||||||||||||||||||||||||||

Sbjct 3841 TAGCCCGCTACGGCAGTACAGGCAGTCCAACCGAAGTTGCTTGCGCTCCCCTCCCCACCC 3900

Query 3901 AGCCTATTCAGCTGGAGCAGCCAACTACAATCCTATCAACAATGCACTCTACCCAACGCG 3960

||||||||||||||||||||||||||||||||||||||||||||||||||||||||||||

Sbjct 3901 AGCCTATTCAGCTGGAGCAGCCAACTACAATCCTATCAACAATGCACTCTACCCAACGCG 3960

Query 3961 ACCAAGCAGCGGTCTCACAGATCCATGGCTCGAAATCTCTCAGGTGAAAACGCAAACTTT 4020

||||||||||||||||||||||||||||||||||||||||||||||||||||||||||||

Sbjct 3961 ACCAAGCAGCGGTCTCACAGATCCATGGCTCGAAATCTCTCAGGTGAAAACGCAAACTTT 4020

Query 4021 TGATTCTCCAAGAAGATTGTAGAGATTCCAAAAAGAAGCAATACTTTGTATACAGGCAGG 4080

||||||||||||||||||||||||||||||||||||||||||||||||||||||||||||

Sbjct 4021 TGATTCTCCAAGAAGATTGTAGAGATTCCAAAAAGAAGCAATACTTTGTATACAGGCAGG 4080

Query 4081 GAAAGAAGTGTTTCATTATTTATGTTGTTAGAGAAACAAAGGAACACCtttttttttttG 4140

||||||||||||||||||||||||||||||||||||||||||||||||||||||||||||

Sbjct 4081 GAAAGAAGTGTTTCATTATTTATGTTGTTAGAGAAACAAAGGAACACCTTTTTTTTTTTG 4140

Query 4141 TGTTTCGCCCTTTCTGTATGTATCTTTCACCCAGGATGCAGTTGCATCGCCTTTGTACAA 4200

||||||||||||||||||||||||||||||||||||||||||||||||||||||||||||

Sbjct 4141 TGTTTCGCCCTTTCTGTATGTATCTTTCACCCAGGATGCAGTTGCATCGCCTTTGTACAA 4200

Query 4201 ATTCAGAGAAGAGCTAGTCAAAAAGAGTAGCATTTCAGATCGCACATCTATATGTTTTGT 4260

||||||||||||||||||||||||||||||||||||||||||||||||||||||||||||

Sbjct 4201 ATTCAGAGAAGAGCTAGTCAAAAAGAGTAGCATTTCAGATCGCACATCTATATGTTTTGT 4260

Query 4261 CCATATGGCTAGATTGTGCACAAGGTGTTAGAGAAAGGAGATTCATGGTAATTGAATCTG 4320

||||||||||||||||||||||||||||||||||||||||||||||||||||||||||||

Sbjct 4261 CCATATGGCTAGATTGTGCACAAGGTGTTAGAGAAAGGAGATTCATGGTAATTGAATCTG 4320

Query 4321 ATGCATATGGAACAC 4335

|||||||||||||||

Sbjct 4321 ATGCATATGGAACAC 4335

Query: GGRMZM6G513881.

Subject: isotig03084 gene=isogroup00379.

| Alignment statistics for match #1 | | | | |
| --- | --- | --- | --- | --- |
| **Score** | **Expect** | **Identities** | **Gaps** | **Strand** |
| 726 bits(804) | 0.0 | 610/744(82%) | 37/744(4%) | Plus/Plus |

Query 3594 ATTGAAACTTCGTCATACAGAAGTTTATCTCCTTTAAGAGATCCTGATATCCTTGGAAGA 3653

|||| | ||| ||||||||||| |||||||| ||||||||||||||| || | ||||

Sbjct 1715 ATTGCAGTTTCATCATACAGAAGCTTATCTCCATTAAGAGATCCTGATGTCGTGATAAGA 1774

Query 3654 AACTTGCCAGGACCAACATCCCCTATTCCTTCGACATCAAGTCGCAGGATCGCAGCATTG 3713

|| |||| ||||||||||||||| |||||| ||| |||| ||||||||| || ||| ||

Sbjct 1775 AATTTGCAAGGACCAACATCCCCCATTCCTCCGATGTCAAATCGCAGGATTGCGGCAATG 1834

Query 3714 TATGTTCTCCTTCTGTCAGATTTATATCAATTTACATATTGTGAACTGTGTCTCAACCGA 3773

|||||| ||||| ||| ||| || || ||| |||||| | | || ||| || | ||

Sbjct 1835 TATGTTATCCTTTTGTTAGACTT-TA-CAAATTACATCTCATCAATTGTC---CACCTGA 1889

Query 3774 TCTCTC----TTTCGTCCAGGAACACATCCAATGTTCGGATGAACATGTCGCTGCCTGTC 3829

| | || |||| ||||| ||| |||||||| |||||||||||||||| ||||||||

Sbjct 1890 TATATCCTTTTTTCTTCCAGCAACCCATCCAATATTCGGATGAACATGTCCGTGCCTGTC 1949

Query 3830 TCTCCCTGCTCTAGCCCGCTACGGCAGTACAGGCAGTCCAACCGAAGTTGCTTGCGCTCC 3889

||||||||||||||||| ||||||||||| |||||||| || ||||||||||||| ||

Sbjct 1950 TCTCCCTGCTCTAGCCCACTACGGCAGTATAGGCAGTCGAATCGAAGTTGCTTGCCATCG 2009

Query 3890 CCTCCCCACCCAGCCTATTCAGCTGGAGCAGCCAACTACAATCCTATCAACAATGCACTC 3949

||||| || ||||||||||||||||||||||||||||||| ||||||||| || |||||

Sbjct 2010 CCTCCTCATCCAGCCTATTCAGCTGGAGCAGCCAACTACAGTCCTATCAATAACACACTC 2069

Query 3950 TACCCAACGCGACCAAGCAGCGGTCTCACAGATCCATGGCTCGAAATCTCTCAGGTGAAA 4009

|| || | |||||||||||||||||| ||||| ||||||||||||| || ||| |||||

Sbjct 2070 TATCCGATGCGACCAAGCAGCGGTCTAACAGAGCCATGGCTCGAAAACTTTCAACTGAAA 2129

Query 4010 ACGCAAACTTTTGATTCTCCAAGAAGATTGTAGAGATT-CCAAAAAGAAGCAATACTTTG 4068

|| |||||||||||||||||||||||||| |||||||| ||||||||||| ||||| |||

Sbjct 2130 ACACAAACTTTTGATTCTCCAAGAAGATTATAGAGATTCCCAAAAAGAAGTAATACATTG 2189

Query 4069 TATACAGGCAGGGAAAGAAGTGTTTCATTATTTATGTTGTTAGAGAAACAAAGGAACACC 4128

|||| || |||||||| ||| ||||||||| ||||| ||||||||||| |

Sbjct 2190 TATAGAG---------GAAGTGTTCCATAATTTATGTTATTAGAAGAACAAAGGAACTGC 2240

Query 4129 tttttttttttGTGTTTCGCCCTTTCTGTATGTATCTTTCACCCAGGATGCAGTTGCATC 4188

| ||||| |||||| |||||||||||||||| ||||||||||||||||| |||||

Sbjct 2241 CTCTTTTT----TGTTTCACCCTTTCTGTATGTATTTTTCACCCAGGATGCAGACGCATC 2296

Query 4189 GCCTTTGTACAAATTCAGAGAAGAGCTAGTCAAAAAGAGTAGCATTTCAGATCGCACATC 4248

||||||||| |||||||||||||||||||| |||||||||||||||||| |||||

Sbjct 2297 GCCTTTGTATAAATTCAGAGAAGAGCTAGTGAAAAAGAGTAGCATTTCACATCGC----- 2351

Query 4249 TATATGTTTTGTCCATATGGCTAGATTGTGCACAAG--GTGTTAGAGAAAGGAGATTCAT 4306

||||||||||| || |||| | | |||| ||| | ||||||||||||||

Sbjct 2352 ------CTTTGTCCATAT-TCTGGATTTTTCTCAAGATGTGATTCTGAAAGGAGATTCAT 2404

Query 4307 GGTAATTGAATCTGATGCATATGG 4330

|||||||| |||||||||| ||||

Sbjct 2405 GGTAATTGGATCTGATGCACATGG 2428

Range 2: 422 to 909[Graphics](https://www.ncbi.nlm.nih.gov/projects/sviewer/?RID=SUYPNXCX114&id=lcl|Query_89087&tracks=%5bkey:sequence_track,name:Sequence,display_name:Sequence,id:STD1,category:Sequence,annots:Sequence,ShowLabel:true%5d%5bkey:gene_model_track,CDSProductFeats:false%5d%5bkey:alignment_track,name:other%20alignments,annots:NG%20Alignments|Refseq%20Alignments|Gnomon%20Alignments|Unnamed,shown:false%5d&v=398:933&appname=ncbiblast&link_loc=fromHSP)Next MatchPrevious Match[First Match](https://blast.ncbi.nlm.nih.gov/Blast.cgi#hspQuery_89087_1)

| Alignment statistics for match #2 | | | | |
| --- | --- | --- | --- | --- |
| **Score** | **Expect** | **Identities** | **Gaps** | **Strand** |
| 610 bits(676) | 1e-176 | 428/488(88%) | 0/488(0%) | Plus/Plus |

Query 1320 AGGTTGTCAGAAACAAGCAGCACTCTTCTGGGCAGAACAGTAGCAATTGAATCCCGGAAA 1379

||||||||||||||||||||||| |||| |||||||||||| || |||||| ||||||

Sbjct 422 AGGTTGTCAGAAACAAGCAGCACACTTCCTGGCAGAACAGTACCAGCTGAATCTCGGAAA 481

Query 1380 CAAAGTCAGGTGCCAGCAGAGGGGACCATTTTCACCAATAATCAGGCTGTGGAGCATACC 1439

|||||||| |||| |||||| ||| |||||| || |||| ||||||| |||| ||| ||

Sbjct 482 CAAAGTCATGTGCTAGCAGAAGGGCGCATTTTTACAAATAGTCAGGCTTTGGACCATTCC 541

Query 1440 CGGTTGTCTGAAACATCAGTTTCCCCAAGGAAAGAATTTCGCCCTCAAAATTTGGATCTT 1499

|| ||||||||||| |||||||| |||||||||||||||| || ||||||| |||||||

Sbjct 542 CGATTGTCTGAAACCTCAGTTTCTCCAAGGAAAGAATTTCACCTTCAAAATCTGGATCTG 601

Query 1500 GCAAATGATCGAACTACATACTGCCGTGGTCGGAGATCAACCGAAATCGTGTTCAGTACA 1559

|||||||||| ||||| ||||| |||||||||| |||||| ||||| ||||||||| ||

Sbjct 602 GCAAATGATCAAACTAGGTACTGTCGTGGTCGGAAATCAACAGAAATTGTGTTCAGTCCA 661

Query 1560 CAAGTGCCCACTTCTCCTCCTAGTTCAAGAGGACATCACTATCAAAATTCGCCTGTGCCA 1619

||||||||| ||||||| |||| ||| |||||||||||||||| || || ||||||||

Sbjct 662 CAAGTGCCCGCTTCTCCACCTAATTCCAGAGGACATCACTATCCAACCTCCCCTGTGCCG 721

Query 1620 TCAAGAACATTTGGGCAATGCCCTGCATCTCCTACTTCATGGCAGGATGATTCGCGAAGC 1679

||||||||||||||||| |||||||||||||||||||||||||||| ||||| ||||||

Sbjct 722 ACAAGAACATTTGGGCAAGGCCCTGCATCTCCTACTTCATGGCAGGAGGATTCCCGAAGC 781

Query 1680 TCAAGCTCACCCCAACCACTTCCTCTTCCTCCAGGTTCCCCATGCTTGCCTTCCTCTTCT 1739

||||||| ||| || || ||||||||||||||||| ||||||||||| |||||| |||

Sbjct 782 TCAAGCTTACCTCAGCCTCTTCCTCTTCCTCCAGGCTCCCCATGCTTACCTTCCCGCTCT 841

Query 1740 CTACAGTGGAAGAAGGGGAAGTTGCTAGGTAGTGGGACGTTTGGGCAAGTATACATGGGA 1799

||||||||||| |||||||||||||| || ||||||||||||||||||||||| |||||

Sbjct 842 CTACAGTGGAAAAAGGGGAAGTTGCTTGGCAGTGGGACGTTTGGGCAAGTATATTTGGGA 901

Query 1800 TTCAACAG 1807

||||||||

Sbjct 902 TTCAACAG 909

Range 3: 1465 to 1704[Graphics](https://www.ncbi.nlm.nih.gov/projects/sviewer/?RID=SUYPNXCX114&id=lcl|Query_89087&tracks=%5bkey:sequence_track,name:Sequence,display_name:Sequence,id:STD1,category:Sequence,annots:Sequence,ShowLabel:true%5d%5bkey:gene_model_track,CDSProductFeats:false%5d%5bkey:alignment_track,name:other%20alignments,annots:NG%20Alignments|Refseq%20Alignments|Gnomon%20Alignments|Unnamed,shown:false%5d&v=1454:1715&appname=ncbiblast&link_loc=fromHSP)Next MatchPrevious Match[First Match](https://blast.ncbi.nlm.nih.gov/Blast.cgi#hspQuery_89087_1)

| Alignment statistics for match #3 | | | | |
| --- | --- | --- | --- | --- |
| **Score** | **Expect** | **Identities** | **Gaps** | **Strand** |
| 320 bits(354) | 3e-89 | 215/240(90%) | 0/240(0%) | Plus/Plus |

Query 3121 GGTGGCAGCAATATTCAAAATTGGCAACAGCAAAGACATACCAGATATCCCAAATAATCT 3180

|||||| |||||||| || ||||| ||||||||||||||||| ||||||||| || ||||

Sbjct 1465 GGTGGCTGCAATATTTAAGATTGGAAACAGCAAAGACATACCTGATATCCCAGATCATCT 1524

Query 3181 TTCTTCTGAGGCAAAAAGTTTCCTGAAACTCTGCTTGCAGCGTGATCCTGCTGCCCGCCC 3240

|||| ||||||| ||||| || || ||||| || ||||||||||||||||||||||| ||

Sbjct 1525 TTCTCCTGAGGCGAAAAGCTTTCTTAAACTATGTTTGCAGCGTGATCCTGCTGCCCGGCC 1584

Query 3241 TACAGCTGCTCAGCTGATGGATCACCCTTTTGTCAAGGACCAGGCTACAGTCAGGAGTTC 3300

||| ||||||||| |||||||||||||||||||||||||||| |||||||| ||||||||

Sbjct 1585 TACTGCTGCTCAGTTGATGGATCACCCTTTTGTCAAGGACCATGCTACAGTTAGGAGTTC 1644

Query 3301 CAGGTCCAGTATCACAAGGGATATGTTTCCTAATTCAACTGACGGAAAAAACAGCAGGGT 3360

||||||||| | | |||||||||||||||||| ||||||||| ||||||||||||| |||

Sbjct 1645 CAGGTCCAGCACCCCAAGGGATATGTTTCCTACTTCAACTGATGGAAAAAACAGCATGGT 1704

Range 4: 146 to 425[Graphics](https://www.ncbi.nlm.nih.gov/projects/sviewer/?RID=SUYPNXCX114&id=lcl|Query_89087&tracks=%5bkey:sequence_track,name:Sequence,display_name:Sequence,id:STD1,category:Sequence,annots:Sequence,ShowLabel:true%5d%5bkey:gene_model_track,CDSProductFeats:false%5d%5bkey:alignment_track,name:other%20alignments,annots:NG%20Alignments|Refseq%20Alignments|Gnomon%20Alignments|Unnamed,shown:false%5d&v=133:438&appname=ncbiblast&link_loc=fromHSP)Next MatchPrevious Match[First Match](https://blast.ncbi.nlm.nih.gov/Blast.cgi#hspQuery_89087_1)

| Alignment statistics for match #4 | | | | |
| --- | --- | --- | --- | --- |
| **Score** | **Expect** | **Identities** | **Gaps** | **Strand** |
| 297 bits(328) | 4e-82 | 242/291(83%) | 16/291(5%) | Plus/Plus |

Query 1 GCCGCTGCCATCGGCGATGTGAAGGAGGAGAAGGGGAAGAAGAAGGCGAGCAGCTTCGAC 60

||||| ||| ||| || || ||||||| |||||||||||||||||||||||||||||||

Sbjct 146 GCCGCCGCCGCCGGGGAGGTTAAGGAGGCGAAGGGGAAGAAGAAGGCGAGCAGCTTCGAC 205

Query 61 GAGGCGCTCCTCGCCAAGGGCGTCCGCGGGAAGCAGCAGCATGCGCCGGCGGCGGCGG-- 118

|||||||| ||||||||||| |||||||||||||||||||| |||| |||||||||

Sbjct 206 GAGGCGCTGCTCGCCAAGGGAGTCCGCGGGAAGCAGCAGCAGCAGCCGCCGGCGGCGGCT 265

Query 119 GGGAGGGTATGGGGCTCCCGCTCCCGCTCCCGCGCCCGGCGTCCTTGCCGACGCCGCTGC 178

| ||| || || ||||||||||||||| ||||||||||||||| |||||||||

Sbjct 266 GCCTCGGT-TG----TCGGGCTCCCGCTCCCGCGGCCGGCGTCCTTGCCGGCGCCGCTGC 320

Query 179 CGTCCGCGTCCGCGTCGGCCTCCGCCTCGGCGTCGGCGTCCAGCGGCGGCGACTCCTCGC 238

|||| |||||||| ||||| |||||| |||| |||||||||||||| |||||||

Sbjct 321 CGTCTGCGTCCGCCTCGGCATCCGCC------TCGGGGTCCAGCGGCGGCGGATCCTCGC 374

Query 239 TGGGGTCCT---CCACGTCCGACGACCAGCTGGATCTCGGGGTTTACAGGT 286

||| ||||| | |||||||||| ||||||||| |||| ||||||||||

Sbjct 375 TGGTGTCCTCGGCGGCGTCCGACGAGCAGCTGGATTTCGGTGTTTACAGGT 425

Range 5: 1064 to 1225[Graphics](https://www.ncbi.nlm.nih.gov/projects/sviewer/?RID=SUYPNXCX114&id=lcl|Query_89087&tracks=%5bkey:sequence_track,name:Sequence,display_name:Sequence,id:STD1,category:Sequence,annots:Sequence,ShowLabel:true%5d%5bkey:gene_model_track,CDSProductFeats:false%5d%5bkey:alignment_track,name:other%20alignments,annots:NG%20Alignments|Refseq%20Alignments|Gnomon%20Alignments|Unnamed,shown:false%5d&v=1056:1233&appname=ncbiblast&link_loc=fromHSP)Next MatchPrevious Match[First Match](https://blast.ncbi.nlm.nih.gov/Blast.cgi#hspQuery_89087_1)

| Alignment statistics for match #5 | | | | |
| --- | --- | --- | --- | --- |
| **Score** | **Expect** | **Identities** | **Gaps** | **Strand** |
| 257 bits(284) | 3e-70 | 154/162(95%) | 0/162(0%) | Plus/Plus |

Query 2311 AATGAGACACTCTCGGTCTATCTCGAGTATGTTTCTGGGGGCTCTATCCATAAGTTGCTT 2370

||||||||||||||||||||||||||||| |||||||||||||| |||||||||||| ||

Sbjct 1064 AATGAGACACTCTCGGTCTATCTCGAGTACGTTTCTGGGGGCTCCATCCATAAGTTGATT 1123

Query 2371 CAAGAATATGGTCCGTTTGGGGAGGCAGTGCTTCGGAATTACACAGCACAAATCCTTTCT 2430

||||||||||||||||||||||||||||| |||||||||||||| || ||||||||||||

Sbjct 1124 CAAGAATATGGTCCGTTTGGGGAGGCAGTTCTTCGGAATTACACTGCGCAAATCCTTTCT 1183

Query 2431 GGCCTTGCATACTTGCATGGGCGGAATACAGTGCATAGGTAT 2472

|| |||||||||||||||||||||||||||||||||||| ||

Sbjct 1184 GGTCTTGCATACTTGCATGGGCGGAATACAGTGCATAGGGAT 1225

Range 6: 1356 to 1468[Graphics](https://www.ncbi.nlm.nih.gov/projects/sviewer/?RID=SUYPNXCX114&id=lcl|Query_89087&tracks=%5bkey:sequence_track,name:Sequence,display_name:Sequence,id:STD1,category:Sequence,annots:Sequence,ShowLabel:true%5d%5bkey:gene_model_track,CDSProductFeats:false%5d%5bkey:alignment_track,name:other%20alignments,annots:NG%20Alignments|Refseq%20Alignments|Gnomon%20Alignments|Unnamed,shown:false%5d&v=1351:1473&appname=ncbiblast&link_loc=fromHSP)Next MatchPrevious Match[First Match](https://blast.ncbi.nlm.nih.gov/Blast.cgi#hspQuery_89087_1)

| Alignment statistics for match #6 | | | | |
| --- | --- | --- | --- | --- |
| **Score** | **Expect** | **Identities** | **Gaps** | **Strand** |
| 196 bits(216) | 1e-51 | 111/113(98%) | 0/113(0%) | Plus/Plus |

Query 2910 AGGTTATTATGAATAGCAATGGTTACAGCCTTTCAGTAGACATTTGGAGCCTTGGCTGCA 2969

||||||| ||||||||||||||||||||||||||||||||||||||||||||||||||||

Sbjct 1356 AGGTTATCATGAATAGCAATGGTTACAGCCTTTCAGTAGACATTTGGAGCCTTGGCTGCA 1415

Query 2970 CCATTCTTGAGATGGCAACAGCAAAGCCTCCTTGGAGTCAGTATGAAGGGGTG 3022

||||||||||||||||||||||||| |||||||||||||||||||||||||||

Sbjct 1416 CCATTCTTGAGATGGCAACAGCAAAACCTCCTTGGAGTCAGTATGAAGGGGTG 1468

Range 7: 907 to 998[Graphics](https://www.ncbi.nlm.nih.gov/projects/sviewer/?RID=SUYPNXCX114&id=lcl|Query_89087&tracks=%5bkey:sequence_track,name:Sequence,display_name:Sequence,id:STD1,category:Sequence,annots:Sequence,ShowLabel:true%5d%5bkey:gene_model_track,CDSProductFeats:false%5d%5bkey:alignment_track,name:other%20alignments,annots:NG%20Alignments|Refseq%20Alignments|Gnomon%20Alignments|Unnamed,shown:false%5d&v=903:1002&appname=ncbiblast&link_loc=fromHSP)Next MatchPrevious Match[First Match](https://blast.ncbi.nlm.nih.gov/Blast.cgi#hspQuery_89087_1)

| Alignment statistics for match #7 | | | | |
| --- | --- | --- | --- | --- |
| **Score** | **Expect** | **Identities** | **Gaps** | **Strand** |
| 152 bits(168) | 1e-38 | 89/92(97%) | 0/92(0%) | Plus/Plus |

Query 1919 CAGTGAAGGTGGTCAAATGTGTGCAATTAAAGAGGTTAAGGTCATTTCAGATGATTCTAA 1978

|||||||||||||||||||||||||||||||||||||||||||||||| |||||||||||

Sbjct 907 CAGTGAAGGTGGTCAAATGTGTGCAATTAAAGAGGTTAAGGTCATTTCTGATGATTCTAA 966

Query 1979 CTCAAAGGAGTCCCTCAGGCAGCTAAATCAGG 2010

|||||| |||| ||||||||||||||||||||

Sbjct 967 CTCAAAAGAGTGCCTCAGGCAGCTAAATCAGG 998

Range 8: 1220 to 1300[Graphics](https://www.ncbi.nlm.nih.gov/projects/sviewer/?RID=SUYPNXCX114&id=lcl|Query_89087&tracks=%5bkey:sequence_track,name:Sequence,display_name:Sequence,id:STD1,category:Sequence,annots:Sequence,ShowLabel:true%5d%5bkey:gene_model_track,CDSProductFeats:false%5d%5bkey:alignment_track,name:other%20alignments,annots:NG%20Alignments|Refseq%20Alignments|Gnomon%20Alignments|Unnamed,shown:false%5d&v=1216:1304&appname=ncbiblast&link_loc=fromHSP)Next MatchPrevious Match[First Match](https://blast.ncbi.nlm.nih.gov/Blast.cgi#hspQuery_89087_1)

| Alignment statistics for match #8 | | | | |
| --- | --- | --- | --- | --- |
| **Score** | **Expect** | **Identities** | **Gaps** | **Strand** |
| 123 bits(136) | 5e-30 | 76/81(94%) | 0/81(0%) | Plus/Plus |

Query 2583 AGGGATATCAAAGGGGCAAACATACTTGTAGATCCTAATGGTGACATCAAGCTTGCTGAT 2642

||||||||||||||||||||||||||||| ||||||||||||||||||||||||||||||

Sbjct 1220 AGGGATATCAAAGGGGCAAACATACTTGTCGATCCTAATGGTGACATCAAGCTTGCTGAT 1279

Query 2643 TTTGGTATGGCGAAACATGTA 2663

||||| ||||| || ||| ||

Sbjct 1280 TTTGGCATGGCCAAGCATATA 1300

Range 9: 995 to 1060[Graphics](https://www.ncbi.nlm.nih.gov/projects/sviewer/?RID=SUYPNXCX114&id=lcl|Query_89087&tracks=%5bkey:sequence_track,name:Sequence,display_name:Sequence,id:STD1,category:Sequence,annots:Sequence,ShowLabel:true%5d%5bkey:gene_model_track,CDSProductFeats:false%5d%5bkey:alignment_track,name:other%20alignments,annots:NG%20Alignments|Refseq%20Alignments|Gnomon%20Alignments|Unnamed,shown:false%5d&v=992:1063&appname=ncbiblast&link_loc=fromHSP)Next MatchPrevious Match[First Match](https://blast.ncbi.nlm.nih.gov/Blast.cgi#hspQuery_89087_1)

| Alignment statistics for match #9 | | | | |
| --- | --- | --- | --- | --- |
| **Score** | **Expect** | **Identities** | **Gaps** | **Strand** |
| 105 bits(116) | 1e-24 | 63/66(95%) | 0/66(0%) | Plus/Plus |

Query 2103 CAGGAAATCGTGCTGCTGAGTCAGCTGTCACATCCAAACATTGTTCAGTACTATGGCAGT 2162

||||||||| |||||||||||||||||||||||||||||||||| |||||||||||||||

Sbjct 995 CAGGAAATCATGCTGCTGAGTCAGCTGTCACATCCAAACATTGTACAGTACTATGGCAGT 1054

Query 2163 GATTTG 2168

||| ||

Sbjct 1055 GATCTG 1060

Range 10: 1295 to 1359[Graphics](https://www.ncbi.nlm.nih.gov/projects/sviewer/?RID=SUYPNXCX114&id=lcl|Query_89087&tracks=%5bkey:sequence_track,name:Sequence,display_name:Sequence,id:STD1,category:Sequence,annots:Sequence,ShowLabel:true%5d%5bkey:gene_model_track,CDSProductFeats:false%5d%5bkey:alignment_track,name:other%20alignments,annots:NG%20Alignments|Refseq%20Alignments|Gnomon%20Alignments|Unnamed,shown:false%5d&v=1292:1362&appname=ncbiblast&link_loc=fromHSP)Next MatchPrevious Match[First Match](https://blast.ncbi.nlm.nih.gov/Blast.cgi#hspQuery_89087_1)

| Alignment statistics for match #10 | | | | |
| --- | --- | --- | --- | --- |
| **Score** | **Expect** | **Identities** | **Gaps** | **Strand** |
| 104 bits(114) | 5e-24 | 62/65(95%) | 0/65(0%) | Plus/Plus |

Query 2757 CAGATATCAGCATATACATCTATCAAATCCTTCAAAGGGAGCCCTTACTGGATGGCACCA 2816

|| ||||||||||| |||||||||| ||||||||||||||||||||||||||||||||||

Sbjct 1295 CATATATCAGCATACACATCTATCAGATCCTTCAAAGGGAGCCCTTACTGGATGGCACCA 1354

Query 2817 GAGGT 2821

|||||

Sbjct 1355 GAGGT 1359

Query: GGRMZM6G513881.

Subject: isotig03083 gene=isogroup00379

| Alignment statistics for match #1 | | | | |
| --- | --- | --- | --- | --- |
| **Score** | **Expect** | **Identities** | **Gaps** | **Strand** |
| 726 bits(804) | 0.0 | 610/744(82%) | 37/744(4%) | Plus/Plus |

Query 3594 ATTGAAACTTCGTCATACAGAAGTTTATCTCCTTTAAGAGATCCTGATATCCTTGGAAGA 3653

|||| | ||| ||||||||||| |||||||| ||||||||||||||| || | ||||

Sbjct 1740 ATTGCAGTTTCATCATACAGAAGCTTATCTCCATTAAGAGATCCTGATGTCGTGATAAGA 1799

Query 3654 AACTTGCCAGGACCAACATCCCCTATTCCTTCGACATCAAGTCGCAGGATCGCAGCATTG 3713

|| |||| ||||||||||||||| |||||| ||| |||| ||||||||| || ||| ||

Sbjct 1800 AATTTGCAAGGACCAACATCCCCCATTCCTCCGATGTCAAATCGCAGGATTGCGGCAATG 1859

Query 3714 TATGTTCTCCTTCTGTCAGATTTATATCAATTTACATATTGTGAACTGTGTCTCAACCGA 3773

|||||| ||||| ||| ||| || || ||| |||||| | | || ||| || | ||

Sbjct 1860 TATGTTATCCTTTTGTTAGACTT-TA-CAAATTACATCTCATCAATTGTC---CACCTGA 1914

Query 3774 TCTCTC----TTTCGTCCAGGAACACATCCAATGTTCGGATGAACATGTCGCTGCCTGTC 3829

| | || |||| ||||| ||| |||||||| |||||||||||||||| ||||||||

Sbjct 1915 TATATCCTTTTTTCTTCCAGCAACCCATCCAATATTCGGATGAACATGTCCGTGCCTGTC 1974

Query 3830 TCTCCCTGCTCTAGCCCGCTACGGCAGTACAGGCAGTCCAACCGAAGTTGCTTGCGCTCC 3889

||||||||||||||||| ||||||||||| |||||||| || ||||||||||||| ||

Sbjct 1975 TCTCCCTGCTCTAGCCCACTACGGCAGTATAGGCAGTCGAATCGAAGTTGCTTGCCATCG 2034

Query 3890 CCTCCCCACCCAGCCTATTCAGCTGGAGCAGCCAACTACAATCCTATCAACAATGCACTC 3949

||||| || ||||||||||||||||||||||||||||||| ||||||||| || |||||

Sbjct 2035 CCTCCTCATCCAGCCTATTCAGCTGGAGCAGCCAACTACAGTCCTATCAATAACACACTC 2094

Query 3950 TACCCAACGCGACCAAGCAGCGGTCTCACAGATCCATGGCTCGAAATCTCTCAGGTGAAA 4009

|| || | |||||||||||||||||| ||||| ||||||||||||| || ||| |||||

Sbjct 2095 TATCCGATGCGACCAAGCAGCGGTCTAACAGAGCCATGGCTCGAAAACTTTCAACTGAAA 2154

Query 4010 ACGCAAACTTTTGATTCTCCAAGAAGATTGTAGAGATT-CCAAAAAGAAGCAATACTTTG 4068

|| |||||||||||||||||||||||||| |||||||| ||||||||||| ||||| |||

Sbjct 2155 ACACAAACTTTTGATTCTCCAAGAAGATTATAGAGATTCCCAAAAAGAAGTAATACATTG 2214

Query 4069 TATACAGGCAGGGAAAGAAGTGTTTCATTATTTATGTTGTTAGAGAAACAAAGGAACACC 4128

|||| || |||||||| ||| ||||||||| ||||| ||||||||||| |

Sbjct 2215 TATAGAG---------GAAGTGTTCCATAATTTATGTTATTAGAAGAACAAAGGAACTGC 2265

Query 4129 tttttttttttGTGTTTCGCCCTTTCTGTATGTATCTTTCACCCAGGATGCAGTTGCATC 4188

| ||||| |||||| |||||||||||||||| ||||||||||||||||| |||||

Sbjct 2266 CTCTTTTT----TGTTTCACCCTTTCTGTATGTATTTTTCACCCAGGATGCAGACGCATC 2321

Query 4189 GCCTTTGTACAAATTCAGAGAAGAGCTAGTCAAAAAGAGTAGCATTTCAGATCGCACATC 4248

||||||||| |||||||||||||||||||| |||||||||||||||||| |||||

Sbjct 2322 GCCTTTGTATAAATTCAGAGAAGAGCTAGTGAAAAAGAGTAGCATTTCACATCGC----- 2376

Query 4249 TATATGTTTTGTCCATATGGCTAGATTGTGCACAAG--GTGTTAGAGAAAGGAGATTCAT 4306

||||||||||| || |||| | | |||| ||| | ||||||||||||||

Sbjct 2377 ------CTTTGTCCATAT-TCTGGATTTTTCTCAAGATGTGATTCTGAAAGGAGATTCAT 2429

Query 4307 GGTAATTGAATCTGATGCATATGG 4330

|||||||| |||||||||| ||||

Sbjct 2430 GGTAATTGGATCTGATGCACATGG 2453

Range 2: 447 to 934[Graphics](https://www.ncbi.nlm.nih.gov/projects/sviewer/?RID=SUYPNXCX114&id=lcl|Query_89086&tracks=%5bkey:sequence_track,name:Sequence,display_name:Sequence,id:STD1,category:Sequence,annots:Sequence,ShowLabel:true%5d%5bkey:gene_model_track,CDSProductFeats:false%5d%5bkey:alignment_track,name:other%20alignments,annots:NG%20Alignments|Refseq%20Alignments|Gnomon%20Alignments|Unnamed,shown:false%5d&v=423:958&appname=ncbiblast&link_loc=fromHSP)Next MatchPrevious Match[First Match](https://blast.ncbi.nlm.nih.gov/Blast.cgi#hspQuery_89086_1)

| Alignment statistics for match #2 | | | | |
| --- | --- | --- | --- | --- |
| **Score** | **Expect** | **Identities** | **Gaps** | **Strand** |
| 610 bits(676) | 1e-176 | 428/488(88%) | 0/488(0%) | Plus/Plus |

Query 1320 AGGTTGTCAGAAACAAGCAGCACTCTTCTGGGCAGAACAGTAGCAATTGAATCCCGGAAA 1379

||||||||||||||||||||||| |||| |||||||||||| || |||||| ||||||

Sbjct 447 AGGTTGTCAGAAACAAGCAGCACACTTCCTGGCAGAACAGTACCAGCTGAATCTCGGAAA 506

Query 1380 CAAAGTCAGGTGCCAGCAGAGGGGACCATTTTCACCAATAATCAGGCTGTGGAGCATACC 1439

|||||||| |||| |||||| ||| |||||| || |||| ||||||| |||| ||| ||

Sbjct 507 CAAAGTCATGTGCTAGCAGAAGGGCGCATTTTTACAAATAGTCAGGCTTTGGACCATTCC 566

Query 1440 CGGTTGTCTGAAACATCAGTTTCCCCAAGGAAAGAATTTCGCCCTCAAAATTTGGATCTT 1499

|| ||||||||||| |||||||| |||||||||||||||| || ||||||| |||||||

Sbjct 567 CGATTGTCTGAAACCTCAGTTTCTCCAAGGAAAGAATTTCACCTTCAAAATCTGGATCTG 626

Query 1500 GCAAATGATCGAACTACATACTGCCGTGGTCGGAGATCAACCGAAATCGTGTTCAGTACA 1559

|||||||||| ||||| ||||| |||||||||| |||||| ||||| ||||||||| ||

Sbjct 627 GCAAATGATCAAACTAGGTACTGTCGTGGTCGGAAATCAACAGAAATTGTGTTCAGTCCA 686

Query 1560 CAAGTGCCCACTTCTCCTCCTAGTTCAAGAGGACATCACTATCAAAATTCGCCTGTGCCA 1619

||||||||| ||||||| |||| ||| |||||||||||||||| || || ||||||||

Sbjct 687 CAAGTGCCCGCTTCTCCACCTAATTCCAGAGGACATCACTATCCAACCTCCCCTGTGCCG 746

Query 1620 TCAAGAACATTTGGGCAATGCCCTGCATCTCCTACTTCATGGCAGGATGATTCGCGAAGC 1679

||||||||||||||||| |||||||||||||||||||||||||||| ||||| ||||||

Sbjct 747 ACAAGAACATTTGGGCAAGGCCCTGCATCTCCTACTTCATGGCAGGAGGATTCCCGAAGC 806

Query 1680 TCAAGCTCACCCCAACCACTTCCTCTTCCTCCAGGTTCCCCATGCTTGCCTTCCTCTTCT 1739

||||||| ||| || || ||||||||||||||||| ||||||||||| |||||| |||

Sbjct 807 TCAAGCTTACCTCAGCCTCTTCCTCTTCCTCCAGGCTCCCCATGCTTACCTTCCCGCTCT 866

Query 1740 CTACAGTGGAAGAAGGGGAAGTTGCTAGGTAGTGGGACGTTTGGGCAAGTATACATGGGA 1799

||||||||||| |||||||||||||| || ||||||||||||||||||||||| |||||

Sbjct 867 CTACAGTGGAAAAAGGGGAAGTTGCTTGGCAGTGGGACGTTTGGGCAAGTATATTTGGGA 926

Query 1800 TTCAACAG 1807

||||||||

Sbjct 927 TTCAACAG 934

Range 3: 1490 to 1729[Graphics](https://www.ncbi.nlm.nih.gov/projects/sviewer/?RID=SUYPNXCX114&id=lcl|Query_89086&tracks=%5bkey:sequence_track,name:Sequence,display_name:Sequence,id:STD1,category:Sequence,annots:Sequence,ShowLabel:true%5d%5bkey:gene_model_track,CDSProductFeats:false%5d%5bkey:alignment_track,name:other%20alignments,annots:NG%20Alignments|Refseq%20Alignments|Gnomon%20Alignments|Unnamed,shown:false%5d&v=1479:1740&appname=ncbiblast&link_loc=fromHSP)Next MatchPrevious Match[First Match](https://blast.ncbi.nlm.nih.gov/Blast.cgi#hspQuery_89086_1)

| Alignment statistics for match #3 | | | | |
| --- | --- | --- | --- | --- |
| **Score** | **Expect** | **Identities** | **Gaps** | **Strand** |
| 320 bits(354) | 3e-89 | 215/240(90%) | 0/240(0%) | Plus/Plus |

Query 3121 GGTGGCAGCAATATTCAAAATTGGCAACAGCAAAGACATACCAGATATCCCAAATAATCT 3180

|||||| |||||||| || ||||| ||||||||||||||||| ||||||||| || ||||

Sbjct 1490 GGTGGCTGCAATATTTAAGATTGGAAACAGCAAAGACATACCTGATATCCCAGATCATCT 1549

Query 3181 TTCTTCTGAGGCAAAAAGTTTCCTGAAACTCTGCTTGCAGCGTGATCCTGCTGCCCGCCC 3240

|||| ||||||| ||||| || || ||||| || ||||||||||||||||||||||| ||

Sbjct 1550 TTCTCCTGAGGCGAAAAGCTTTCTTAAACTATGTTTGCAGCGTGATCCTGCTGCCCGGCC 1609

Query 3241 TACAGCTGCTCAGCTGATGGATCACCCTTTTGTCAAGGACCAGGCTACAGTCAGGAGTTC 3300

||| ||||||||| |||||||||||||||||||||||||||| |||||||| ||||||||

Sbjct 1610 TACTGCTGCTCAGTTGATGGATCACCCTTTTGTCAAGGACCATGCTACAGTTAGGAGTTC 1669

Query 3301 CAGGTCCAGTATCACAAGGGATATGTTTCCTAATTCAACTGACGGAAAAAACAGCAGGGT 3360

||||||||| | | |||||||||||||||||| ||||||||| ||||||||||||| |||

Sbjct 1670 CAGGTCCAGCACCCCAAGGGATATGTTTCCTACTTCAACTGATGGAAAAAACAGCATGGT 1729

Range 4: 172 to 450[Graphics](https://www.ncbi.nlm.nih.gov/projects/sviewer/?RID=SUYPNXCX114&id=lcl|Query_89086&tracks=%5bkey:sequence_track,name:Sequence,display_name:Sequence,id:STD1,category:Sequence,annots:Sequence,ShowLabel:true%5d%5bkey:gene_model_track,CDSProductFeats:false%5d%5bkey:alignment_track,name:other%20alignments,annots:NG%20Alignments|Refseq%20Alignments|Gnomon%20Alignments|Unnamed,shown:false%5d&v=159:463&appname=ncbiblast&link_loc=fromHSP)Next MatchPrevious Match[First Match](https://blast.ncbi.nlm.nih.gov/Blast.cgi#hspQuery_89086_1)

| Alignment statistics for match #4 | | | | |
| --- | --- | --- | --- | --- |
| **Score** | **Expect** | **Identities** | **Gaps** | **Strand** |
| 291 bits(322) | 2e-80 | 238/288(83%) | 12/288(4%) | Plus/Plus |

Query 2 CCGCTGCCATCGGCGATGTGAAGGAGGAGAAGGGGAAGAAGAAGGCGAGCAGCTTCGACG 61

|||| ||| ||| || || ||||||| ||||||||||||||||||||||||||||||||

Sbjct 172 CCGCAGCCGCCGGGGAGGTTAAGGAGGCGAAGGGGAAGAAGAAGGCGAGCAGCTTCGACG 231

Query 62 AGGCGCTCCTCGCCAAGGGCGTCCGCGGGAAGCAGCAGCATGCGCCGGCGGCGGCGGGGG 121

||||||| ||||||||||| |||||||||||||||||||| |||| |||| |||| |

Sbjct 232 AGGCGCTGCTCGCCAAGGGAGTCCGCGGGAAGCAGCAGCAGCAGCCGCCGGCAGCGGCCG 291

Query 122 AGGGTATGGGGCTCCCGCTCCCGCTCCCGCGCCCGGCGTCCTTGCCGACGCCGCTGCCGT 181

| | | | || |||||||||||||| ||||||||||||||| ||||||||||||

Sbjct 292 CCGCTGTTG---TCGGCCTCCCGCTCCCGCGGCCGGCGTCCTTGCCGGCGCCGCTGCCGT 348

Query 182 CCGCGTCCGCGTCGGCCTCCGCCTCGGCGTCGGCGTCCAGCGGCGGCGACTCCTCGCTGG 241

| |||||||| ||||| |||||| |||| |||||||||||||| ||||||||||

Sbjct 349 CTGCGTCCGCCTCGGCATCCGCC------TCGGGGTCCAGCGGCGGCGGATCCTCGCTGG 402

Query 242 GGTCCT---CCACGTCCGACGACCAGCTGGATCTCGGGGTTTACAGGT 286

||||| | |||||||||| ||||||||| |||| ||||||||||

Sbjct 403 TGTCCTCGGCGGCGTCCGACGAGCAGCTGGATTTCGGTGTTTACAGGT 450

Range 5: 1089 to 1250[Graphics](https://www.ncbi.nlm.nih.gov/projects/sviewer/?RID=SUYPNXCX114&id=lcl|Query_89086&tracks=%5bkey:sequence_track,name:Sequence,display_name:Sequence,id:STD1,category:Sequence,annots:Sequence,ShowLabel:true%5d%5bkey:gene_model_track,CDSProductFeats:false%5d%5bkey:alignment_track,name:other%20alignments,annots:NG%20Alignments|Refseq%20Alignments|Gnomon%20Alignments|Unnamed,shown:false%5d&v=1081:1258&appname=ncbiblast&link_loc=fromHSP)Next MatchPrevious Match[First Match](https://blast.ncbi.nlm.nih.gov/Blast.cgi#hspQuery_89086_1)

| Alignment statistics for match #5 | | | | |
| --- | --- | --- | --- | --- |
| **Score** | **Expect** | **Identities** | **Gaps** | **Strand** |
| 257 bits(284) | 3e-70 | 154/162(95%) | 0/162(0%) | Plus/Plus |

Query 2311 AATGAGACACTCTCGGTCTATCTCGAGTATGTTTCTGGGGGCTCTATCCATAAGTTGCTT 2370

||||||||||||||||||||||||||||| |||||||||||||| |||||||||||| ||

Sbjct 1089 AATGAGACACTCTCGGTCTATCTCGAGTACGTTTCTGGGGGCTCCATCCATAAGTTGATT 1148

Query 2371 CAAGAATATGGTCCGTTTGGGGAGGCAGTGCTTCGGAATTACACAGCACAAATCCTTTCT 2430

||||||||||||||||||||||||||||| |||||||||||||| || ||||||||||||

Sbjct 1149 CAAGAATATGGTCCGTTTGGGGAGGCAGTTCTTCGGAATTACACTGCGCAAATCCTTTCT 1208

Query 2431 GGCCTTGCATACTTGCATGGGCGGAATACAGTGCATAGGTAT 2472

|| |||||||||||||||||||||||||||||||||||| ||

Sbjct 1209 GGTCTTGCATACTTGCATGGGCGGAATACAGTGCATAGGGAT 1250

Range 6: 1381 to 1493[Graphics](https://www.ncbi.nlm.nih.gov/projects/sviewer/?RID=SUYPNXCX114&id=lcl|Query_89086&tracks=%5bkey:sequence_track,name:Sequence,display_name:Sequence,id:STD1,category:Sequence,annots:Sequence,ShowLabel:true%5d%5bkey:gene_model_track,CDSProductFeats:false%5d%5bkey:alignment_track,name:other%20alignments,annots:NG%20Alignments|Refseq%20Alignments|Gnomon%20Alignments|Unnamed,shown:false%5d&v=1376:1498&appname=ncbiblast&link_loc=fromHSP)Next MatchPrevious Match[First Match](https://blast.ncbi.nlm.nih.gov/Blast.cgi#hspQuery_89086_1)

| Alignment statistics for match #6 | | | | |
| --- | --- | --- | --- | --- |
| **Score** | **Expect** | **Identities** | **Gaps** | **Strand** |
| 196 bits(216) | 1e-51 | 111/113(98%) | 0/113(0%) | Plus/Plus |

Query 2910 AGGTTATTATGAATAGCAATGGTTACAGCCTTTCAGTAGACATTTGGAGCCTTGGCTGCA 2969

||||||| ||||||||||||||||||||||||||||||||||||||||||||||||||||

Sbjct 1381 AGGTTATCATGAATAGCAATGGTTACAGCCTTTCAGTAGACATTTGGAGCCTTGGCTGCA 1440

Query 2970 CCATTCTTGAGATGGCAACAGCAAAGCCTCCTTGGAGTCAGTATGAAGGGGTG 3022

||||||||||||||||||||||||| |||||||||||||||||||||||||||

Sbjct 1441 CCATTCTTGAGATGGCAACAGCAAAACCTCCTTGGAGTCAGTATGAAGGGGTG 1493

Range 7: 932 to 1023[Graphics](https://www.ncbi.nlm.nih.gov/projects/sviewer/?RID=SUYPNXCX114&id=lcl|Query_89086&tracks=%5bkey:sequence_track,name:Sequence,display_name:Sequence,id:STD1,category:Sequence,annots:Sequence,ShowLabel:true%5d%5bkey:gene_model_track,CDSProductFeats:false%5d%5bkey:alignment_track,name:other%20alignments,annots:NG%20Alignments|Refseq%20Alignments|Gnomon%20Alignments|Unnamed,shown:false%5d&v=928:1027&appname=ncbiblast&link_loc=fromHSP)Next MatchPrevious Match[First Match](https://blast.ncbi.nlm.nih.gov/Blast.cgi#hspQuery_89086_1)

| Alignment statistics for match #7 | | | | |
| --- | --- | --- | --- | --- |
| **Score** | **Expect** | **Identities** | **Gaps** | **Strand** |
| 152 bits(168) | 1e-38 | 89/92(97%) | 0/92(0%) | Plus/Plus |

Query 1919 CAGTGAAGGTGGTCAAATGTGTGCAATTAAAGAGGTTAAGGTCATTTCAGATGATTCTAA 1978

|||||||||||||||||||||||||||||||||||||||||||||||| |||||||||||

Sbjct 932 CAGTGAAGGTGGTCAAATGTGTGCAATTAAAGAGGTTAAGGTCATTTCTGATGATTCTAA 991

Query 1979 CTCAAAGGAGTCCCTCAGGCAGCTAAATCAGG 2010

|||||| |||| ||||||||||||||||||||

Sbjct 992 CTCAAAAGAGTGCCTCAGGCAGCTAAATCAGG 1023

Range 8: 1245 to 1325[Graphics](https://www.ncbi.nlm.nih.gov/projects/sviewer/?RID=SUYPNXCX114&id=lcl|Query_89086&tracks=%5bkey:sequence_track,name:Sequence,display_name:Sequence,id:STD1,category:Sequence,annots:Sequence,ShowLabel:true%5d%5bkey:gene_model_track,CDSProductFeats:false%5d%5bkey:alignment_track,name:other%20alignments,annots:NG%20Alignments|Refseq%20Alignments|Gnomon%20Alignments|Unnamed,shown:false%5d&v=1241:1329&appname=ncbiblast&link_loc=fromHSP)Next MatchPrevious Match[First Match](https://blast.ncbi.nlm.nih.gov/Blast.cgi#hspQuery_89086_1)

| Alignment statistics for match #8 | | | | |
| --- | --- | --- | --- | --- |
| **Score** | **Expect** | **Identities** | **Gaps** | **Strand** |
| 123 bits(136) | 5e-30 | 76/81(94%) | 0/81(0%) | Plus/Plus |

Query 2583 AGGGATATCAAAGGGGCAAACATACTTGTAGATCCTAATGGTGACATCAAGCTTGCTGAT 2642

||||||||||||||||||||||||||||| ||||||||||||||||||||||||||||||

Sbjct 1245 AGGGATATCAAAGGGGCAAACATACTTGTCGATCCTAATGGTGACATCAAGCTTGCTGAT 1304

Query 2643 TTTGGTATGGCGAAACATGTA 2663

||||| ||||| || ||| ||

Sbjct 1305 TTTGGCATGGCCAAGCATATA 1325

Range 9: 1020 to 1085[Graphics](https://www.ncbi.nlm.nih.gov/projects/sviewer/?RID=SUYPNXCX114&id=lcl|Query_89086&tracks=%5bkey:sequence_track,name:Sequence,display_name:Sequence,id:STD1,category:Sequence,annots:Sequence,ShowLabel:true%5d%5bkey:gene_model_track,CDSProductFeats:false%5d%5bkey:alignment_track,name:other%20alignments,annots:NG%20Alignments|Refseq%20Alignments|Gnomon%20Alignments|Unnamed,shown:false%5d&v=1017:1088&appname=ncbiblast&link_loc=fromHSP)Next MatchPrevious Match[First Match](https://blast.ncbi.nlm.nih.gov/Blast.cgi#hspQuery_89086_1)

| Alignment statistics for match #9 | | | | |
| --- | --- | --- | --- | --- |
| **Score** | **Expect** | **Identities** | **Gaps** | **Strand** |
| 105 bits(116) | 1e-24 | 63/66(95%) | 0/66(0%) | Plus/Plus |

Query 2103 CAGGAAATCGTGCTGCTGAGTCAGCTGTCACATCCAAACATTGTTCAGTACTATGGCAGT 2162

||||||||| |||||||||||||||||||||||||||||||||| |||||||||||||||

Sbjct 1020 CAGGAAATCATGCTGCTGAGTCAGCTGTCACATCCAAACATTGTACAGTACTATGGCAGT 1079

Query 2163 GATTTG 2168

||| ||

Sbjct 1080 GATCTG 1085

Range 10: 1320 to 1384[Graphics](https://www.ncbi.nlm.nih.gov/projects/sviewer/?RID=SUYPNXCX114&id=lcl|Query_89086&tracks=%5bkey:sequence_track,name:Sequence,display_name:Sequence,id:STD1,category:Sequence,annots:Sequence,ShowLabel:true%5d%5bkey:gene_model_track,CDSProductFeats:false%5d%5bkey:alignment_track,name:other%20alignments,annots:NG%20Alignments|Refseq%20Alignments|Gnomon%20Alignments|Unnamed,shown:false%5d&v=1317:1387&appname=ncbiblast&link_loc=fromHSP)Next MatchPrevious Match[First Match](https://blast.ncbi.nlm.nih.gov/Blast.cgi#hspQuery_89086_1)

| Alignment statistics for match #10 | | | | |
| --- | --- | --- | --- | --- |
| **Score** | **Expect** | **Identities** | **Gaps** | **Strand** |
| 104 bits(114) | 5e-24 | 62/65(95%) | 0/65(0%) | Plus/Plus |

Query 2757 CAGATATCAGCATATACATCTATCAAATCCTTCAAAGGGAGCCCTTACTGGATGGCACCA 2816

|| ||||||||||| |||||||||| ||||||||||||||||||||||||||||||||||

Sbjct 1320 CATATATCAGCATACACATCTATCAGATCCTTCAAAGGGAGCCCTTACTGGATGGCACCA 1379

Query 2817 GAGGT 2821

|||||

Sbjct 1380 GAGGT 1384

Query: GGRMZM6G513881.

Subject: isotig08547 gene=isogroup02509

| Alignment statistics for match #1 | | | | |
| --- | --- | --- | --- | --- |
| **Score** | **Expect** | **Identities** | **Gaps** | **Strand** |
| 610 bits(676) | 1e-176 | 428/488(88%) | 0/488(0%) | Plus/Plus |

Query 1320 AGGTTGTCAGAAACAAGCAGCACTCTTCTGGGCAGAACAGTAGCAATTGAATCCCGGAAA 1379

||||||||||||||||||||||| |||| |||||||||||| || |||||| ||||||

Sbjct 449 AGGTTGTCAGAAACAAGCAGCACACTTCCTGGCAGAACAGTACCAGCTGAATCTCGGAAA 508

Query 1380 CAAAGTCAGGTGCCAGCAGAGGGGACCATTTTCACCAATAATCAGGCTGTGGAGCATACC 1439

|||||||| |||| |||||| ||| |||||| || |||| ||||||| |||| ||| ||

Sbjct 509 CAAAGTCATGTGCTAGCAGAAGGGCGCATTTTTACAAATAGTCAGGCTTTGGACCATTCC 568

Query 1440 CGGTTGTCTGAAACATCAGTTTCCCCAAGGAAAGAATTTCGCCCTCAAAATTTGGATCTT 1499

|| ||||||||||| |||||||| |||||||||||||||| || ||||||| |||||||

Sbjct 569 CGATTGTCTGAAACCTCAGTTTCTCCAAGGAAAGAATTTCACCTTCAAAATCTGGATCTG 628

Query 1500 GCAAATGATCGAACTACATACTGCCGTGGTCGGAGATCAACCGAAATCGTGTTCAGTACA 1559

|||||||||| ||||| ||||| |||||||||| |||||| ||||| ||||||||| ||

Sbjct 629 GCAAATGATCAAACTAGGTACTGTCGTGGTCGGAAATCAACAGAAATTGTGTTCAGTCCA 688

Query 1560 CAAGTGCCCACTTCTCCTCCTAGTTCAAGAGGACATCACTATCAAAATTCGCCTGTGCCA 1619

||||||||| ||||||| |||| ||| |||||||||||||||| || || ||||||||

Sbjct 689 CAAGTGCCCGCTTCTCCACCTAATTCCAGAGGACATCACTATCCAACCTCCCCTGTGCCG 748

Query 1620 TCAAGAACATTTGGGCAATGCCCTGCATCTCCTACTTCATGGCAGGATGATTCGCGAAGC 1679

||||||||||||||||| |||||||||||||||||||||||||||| ||||| ||||||

Sbjct 749 ACAAGAACATTTGGGCAAGGCCCTGCATCTCCTACTTCATGGCAGGAGGATTCCCGAAGC 808

Query 1680 TCAAGCTCACCCCAACCACTTCCTCTTCCTCCAGGTTCCCCATGCTTGCCTTCCTCTTCT 1739

||||||| ||| || || ||||||||||||||||| ||||||||||| |||||| |||

Sbjct 809 TCAAGCTTACCTCAGCCTCTTCCTCTTCCTCCAGGCTCCCCATGCTTACCTTCCCGCTCT 868

Query 1740 CTACAGTGGAAGAAGGGGAAGTTGCTAGGTAGTGGGACGTTTGGGCAAGTATACATGGGA 1799

||||||||||| |||||||||||||| || ||||||||||||||||||||||| |||||

Sbjct 869 CTACAGTGGAAAAAGGGGAAGTTGCTTGGCAGTGGGACGTTTGGGCAAGTATATTTGGGA 928

Query 1800 TTCAACAG 1807

||||||||

Sbjct 929 TTCAACAG 936

Range 2: 1862 to 2229[Graphics](https://www.ncbi.nlm.nih.gov/projects/sviewer/?RID=SUYPNXCX114&id=lcl|Query_89090&tracks=%5bkey:sequence_track,name:Sequence,display_name:Sequence,id:STD1,category:Sequence,annots:Sequence,ShowLabel:true%5d%5bkey:gene_model_track,CDSProductFeats:false%5d%5bkey:alignment_track,name:other%20alignments,annots:NG%20Alignments|Refseq%20Alignments|Gnomon%20Alignments|Unnamed,shown:false%5d&v=1844:2247&appname=ncbiblast&link_loc=fromHSP)Next MatchPrevious Match[First Match](https://blast.ncbi.nlm.nih.gov/Blast.cgi#hspQuery_89090_1)

| Alignment statistics for match #2 | | | | |
| --- | --- | --- | --- | --- |
| **Score** | **Expect** | **Identities** | **Gaps** | **Strand** |
| 434 bits(480) | 2e-123 | 325/381(85%) | 14/381(3%) | Plus/Plus |

Query 3791 AACACATCCAATGTTCGGATGAACATGTCGCTGCCTGTCTCTCCCTGCTCTAGCCCGCTA 3850

||| |||||||| |||||||||||||||| ||||||||||||||||||||||||| |||

Sbjct 1862 AACCCATCCAATATTCGGATGAACATGTCCGTGCCTGTCTCTCCCTGCTCTAGCCCACTA 1921

Query 3851 CGGCAGTACAGGCAGTCCAACCGAAGTTGCTTGCGCTCCCCTCCCCACCCAGCCTATTCA 3910

|||||||| |||||||| || ||||||||||||| || ||||| || ||||||||||||

Sbjct 1922 CGGCAGTATAGGCAGTCGAATCGAAGTTGCTTGCCATCGCCTCCTCATCCAGCCTATTCA 1981

Query 3911 GCTGGAGCAGCCAACTACAATCCTATCAACAATGCACTCTACCCAACGCGACCAAGCAGC 3970

||||||||||||||||||| ||||||||| || ||||||| || | |||||||||||||

Sbjct 1982 GCTGGAGCAGCCAACTACAGTCCTATCAATAACACACTCTATCCGATGCGACCAAGCAGC 2041

Query 3971 GGTCTCACAGATCCATGGCTCGAAATCTCTCAGGTGAAAACGCAAACTTTTGATTCTCCA 4030

||||| ||||| ||||||||||||| || ||| ||||||| ||||||||||||||||||

Sbjct 2042 GGTCTAACAGAGCCATGGCTCGAAAACTTTCAACTGAAAACACAAACTTTTGATTCTCCA 2101

Query 4031 AGAAGATTGTAGAGATT-CCAAAAAGAAGCAATACTTTGTATACAGGCAGGGAAAGAAGT 4089

|||||||| |||||||| ||||||||||| ||||| ||||||| || |||||

Sbjct 2102 AGAAGATTATAGAGATTCCCAAAAAGAAGTAATACATTGTATAGAG---------GAAGT 2152

Query 4090 GTTTCATTATTTATGTTGTTAGAGAAACAAAGGAACACCtttttttttttGTGTTTCGCC 4149

||| ||| ||||||||| ||||| ||||||||||| | | ||||| |||||| ||

Sbjct 2153 GTTCCATAATTTATGTTATTAGAAGAACAAAGGAACTGCCTCTTTTT----TGTTTCACC 2208

Query 4150 CTTTCTGTATGTATCTTTCAC 4170

|||||||||||||| ||||||

Sbjct 2209 CTTTCTGTATGTATTTTTCAC 2229

Range 3: 1492 to 1731[Graphics](https://www.ncbi.nlm.nih.gov/projects/sviewer/?RID=SUYPNXCX114&id=lcl|Query_89090&tracks=%5bkey:sequence_track,name:Sequence,display_name:Sequence,id:STD1,category:Sequence,annots:Sequence,ShowLabel:true%5d%5bkey:gene_model_track,CDSProductFeats:false%5d%5bkey:alignment_track,name:other%20alignments,annots:NG%20Alignments|Refseq%20Alignments|Gnomon%20Alignments|Unnamed,shown:false%5d&v=1481:1742&appname=ncbiblast&link_loc=fromHSP)Next MatchPrevious Match[First Match](https://blast.ncbi.nlm.nih.gov/Blast.cgi#hspQuery_89090_1)

| Alignment statistics for match #3 | | | | |
| --- | --- | --- | --- | --- |
| **Score** | **Expect** | **Identities** | **Gaps** | **Strand** |
| 320 bits(354) | 3e-89 | 215/240(90%) | 0/240(0%) | Plus/Plus |

Query 3121 GGTGGCAGCAATATTCAAAATTGGCAACAGCAAAGACATACCAGATATCCCAAATAATCT 3180

|||||| |||||||| || ||||| ||||||||||||||||| ||||||||| || ||||

Sbjct 1492 GGTGGCTGCAATATTTAAGATTGGAAACAGCAAAGACATACCTGATATCCCAGATCATCT 1551

Query 3181 TTCTTCTGAGGCAAAAAGTTTCCTGAAACTCTGCTTGCAGCGTGATCCTGCTGCCCGCCC 3240

|||| ||||||| ||||| || || ||||| || ||||||||||||||||||||||| ||

Sbjct 1552 TTCTCCTGAGGCGAAAAGCTTTCTTAAACTATGTTTGCAGCGTGATCCTGCTGCCCGGCC 1611

Query 3241 TACAGCTGCTCAGCTGATGGATCACCCTTTTGTCAAGGACCAGGCTACAGTCAGGAGTTC 3300

||| ||||||||| |||||||||||||||||||||||||||| |||||||| ||||||||

Sbjct 1612 TACTGCTGCTCAGTTGATGGATCACCCTTTTGTCAAGGACCATGCTACAGTTAGGAGTTC 1671

Query 3301 CAGGTCCAGTATCACAAGGGATATGTTTCCTAATTCAACTGACGGAAAAAACAGCAGGGT 3360

||||||||| | | |||||||||||||||||| ||||||||| ||||||||||||| |||

Sbjct 1672 CAGGTCCAGCACCCCAAGGGATATGTTTCCTACTTCAACTGATGGAAAAAACAGCATGGT 1731

Range 4: 171 to 452[Graphics](https://www.ncbi.nlm.nih.gov/projects/sviewer/?RID=SUYPNXCX114&id=lcl|Query_89090&tracks=%5bkey:sequence_track,name:Sequence,display_name:Sequence,id:STD1,category:Sequence,annots:Sequence,ShowLabel:true%5d%5bkey:gene_model_track,CDSProductFeats:false%5d%5bkey:alignment_track,name:other%20alignments,annots:NG%20Alignments|Refseq%20Alignments|Gnomon%20Alignments|Unnamed,shown:false%5d&v=157:466&appname=ncbiblast&link_loc=fromHSP)Next MatchPrevious Match[First Match](https://blast.ncbi.nlm.nih.gov/Blast.cgi#hspQuery_89090_1)

| Alignment statistics for match #4 | | | | |
| --- | --- | --- | --- | --- |
| **Score** | **Expect** | **Identities** | **Gaps** | **Strand** |
| 291 bits(322) | 2e-80 | 241/291(83%) | 15/291(5%) | Plus/Plus |

Query 2 CCGCTGCCATCGGCGATGTGAAGGAGGAGAAGGGGAAGAAGAAGGCGAGCAGCTTCGACG 61

|||| ||| ||| || || ||||||| ||||||||||||||||||||||||||||||||

Sbjct 171 CCGCAGCCGCCGGGGAGGTTAAGGAGGCGAAGGGGAAGAAGAAGGCGAGCAGCTTCGACG 230

Query 62 AGGCGCTCCTCGCCAAGGGCGTCCGCGGGAAGCAGCAGCA--TGC-GCCGGCGGCGGCGG 118

||||||| ||||||||||| |||||||||||||||||||| || |||| |||||||||

Sbjct 231 AGGCGCTGCTCGCCAAGGGAGTCCGCGGGAAGCAGCAGCAGCAGCAGCCGCCGGCGGCGG 290

Query 119 GGGAGGGTATGGGGCTCCCGCTCCCGCTCCCGCGCCCGGCGTCCTTGCCGACGCCGCTGC 178

| | | | | || |||||||||||||| ||||||||||||||| |||||||||

Sbjct 291 CCGCCGCTGTTG---TCGGCCTCCCGCTCCCGCGGCCGGCGTCCTTGCCGGCGCCGCTGC 347

Query 179 CGTCCGCGTCCGCGTCGGCCTCCGCCTCGGCGTCGGCGTCCAGCGGCGGCGACTCCTCGC 238

|||| |||||||| ||||| |||||| |||| |||||||||||||| |||||||

Sbjct 348 CGTCTGCGTCCGCCTCGGCATCCGCC------TCGGGGTCCAGCGGCGGCGGATCCTCGC 401

Query 239 TGGGGTCCT---CCACGTCCGACGACCAGCTGGATCTCGGGGTTTACAGGT 286

||| ||||| | |||||||||| ||||||||| |||| ||||||||||

Sbjct 402 TGGTGTCCTCGGCGGCGTCCGACGAGCAGCTGGATTTCGGTGTTTACAGGT 452

Range 5: 1091 to 1252[Graphics](https://www.ncbi.nlm.nih.gov/projects/sviewer/?RID=SUYPNXCX114&id=lcl|Query_89090&tracks=%5bkey:sequence_track,name:Sequence,display_name:Sequence,id:STD1,category:Sequence,annots:Sequence,ShowLabel:true%5d%5bkey:gene_model_track,CDSProductFeats:false%5d%5bkey:alignment_track,name:other%20alignments,annots:NG%20Alignments|Refseq%20Alignments|Gnomon%20Alignments|Unnamed,shown:false%5d&v=1083:1260&appname=ncbiblast&link_loc=fromHSP)Next MatchPrevious Match[First Match](https://blast.ncbi.nlm.nih.gov/Blast.cgi#hspQuery_89090_1)

| Alignment statistics for match #5 | | | | |
| --- | --- | --- | --- | --- |
| **Score** | **Expect** | **Identities** | **Gaps** | **Strand** |
| 257 bits(284) | 3e-70 | 154/162(95%) | 0/162(0%) | Plus/Plus |

Query 2311 AATGAGACACTCTCGGTCTATCTCGAGTATGTTTCTGGGGGCTCTATCCATAAGTTGCTT 2370

||||||||||||||||||||||||||||| |||||||||||||| |||||||||||| ||

Sbjct 1091 AATGAGACACTCTCGGTCTATCTCGAGTACGTTTCTGGGGGCTCCATCCATAAGTTGATT 1150

Query 2371 CAAGAATATGGTCCGTTTGGGGAGGCAGTGCTTCGGAATTACACAGCACAAATCCTTTCT 2430

||||||||||||||||||||||||||||| |||||||||||||| || ||||||||||||

Sbjct 1151 CAAGAATATGGTCCGTTTGGGGAGGCAGTTCTTCGGAATTACACTGCGCAAATCCTTTCT 1210

Query 2431 GGCCTTGCATACTTGCATGGGCGGAATACAGTGCATAGGTAT 2472

|| |||||||||||||||||||||||||||||||||||| ||

Sbjct 1211 GGTCTTGCATACTTGCATGGGCGGAATACAGTGCATAGGGAT 1252

Range 6: 1383 to 1495[Graphics](https://www.ncbi.nlm.nih.gov/projects/sviewer/?RID=SUYPNXCX114&id=lcl|Query_89090&tracks=%5bkey:sequence_track,name:Sequence,display_name:Sequence,id:STD1,category:Sequence,annots:Sequence,ShowLabel:true%5d%5bkey:gene_model_track,CDSProductFeats:false%5d%5bkey:alignment_track,name:other%20alignments,annots:NG%20Alignments|Refseq%20Alignments|Gnomon%20Alignments|Unnamed,shown:false%5d&v=1378:1500&appname=ncbiblast&link_loc=fromHSP)Next MatchPrevious Match[First Match](https://blast.ncbi.nlm.nih.gov/Blast.cgi#hspQuery_89090_1)

| Alignment statistics for match #6 | | | | |
| --- | --- | --- | --- | --- |
| **Score** | **Expect** | **Identities** | **Gaps** | **Strand** |
| 196 bits(216) | 1e-51 | 111/113(98%) | 0/113(0%) | Plus/Plus |

Query 2910 AGGTTATTATGAATAGCAATGGTTACAGCCTTTCAGTAGACATTTGGAGCCTTGGCTGCA 2969

||||||| ||||||||||||||||||||||||||||||||||||||||||||||||||||

Sbjct 1383 AGGTTATCATGAATAGCAATGGTTACAGCCTTTCAGTAGACATTTGGAGCCTTGGCTGCA 1442

Query 2970 CCATTCTTGAGATGGCAACAGCAAAGCCTCCTTGGAGTCAGTATGAAGGGGTG 3022

||||||||||||||||||||||||| |||||||||||||||||||||||||||

Sbjct 1443 CCATTCTTGAGATGGCAACAGCAAAACCTCCTTGGAGTCAGTATGAAGGGGTG 1495

Range 7: 934 to 1025[Graphics](https://www.ncbi.nlm.nih.gov/projects/sviewer/?RID=SUYPNXCX114&id=lcl|Query_89090&tracks=%5bkey:sequence_track,name:Sequence,display_name:Sequence,id:STD1,category:Sequence,annots:Sequence,ShowLabel:true%5d%5bkey:gene_model_track,CDSProductFeats:false%5d%5bkey:alignment_track,name:other%20alignments,annots:NG%20Alignments|Refseq%20Alignments|Gnomon%20Alignments|Unnamed,shown:false%5d&v=930:1029&appname=ncbiblast&link_loc=fromHSP)Next MatchPrevious Match[First Match](https://blast.ncbi.nlm.nih.gov/Blast.cgi#hspQuery_89090_1)

| Alignment statistics for match #7 | | | | |
| --- | --- | --- | --- | --- |
| **Score** | **Expect** | **Identities** | **Gaps** | **Strand** |
| 152 bits(168) | 1e-38 | 89/92(97%) | 0/92(0%) | Plus/Plus |

Query 1919 CAGTGAAGGTGGTCAAATGTGTGCAATTAAAGAGGTTAAGGTCATTTCAGATGATTCTAA 1978

|||||||||||||||||||||||||||||||||||||||||||||||| |||||||||||

Sbjct 934 CAGTGAAGGTGGTCAAATGTGTGCAATTAAAGAGGTTAAGGTCATTTCTGATGATTCTAA 993

Query 1979 CTCAAAGGAGTCCCTCAGGCAGCTAAATCAGG 2010

|||||| |||| ||||||||||||||||||||

Sbjct 994 CTCAAAAGAGTGCCTCAGGCAGCTAAATCAGG 1025

Range 8: 1247 to 1327[Graphics](https://www.ncbi.nlm.nih.gov/projects/sviewer/?RID=SUYPNXCX114&id=lcl|Query_89090&tracks=%5bkey:sequence_track,name:Sequence,display_name:Sequence,id:STD1,category:Sequence,annots:Sequence,ShowLabel:true%5d%5bkey:gene_model_track,CDSProductFeats:false%5d%5bkey:alignment_track,name:other%20alignments,annots:NG%20Alignments|Refseq%20Alignments|Gnomon%20Alignments|Unnamed,shown:false%5d&v=1243:1331&appname=ncbiblast&link_loc=fromHSP)Next MatchPrevious Match[First Match](https://blast.ncbi.nlm.nih.gov/Blast.cgi#hspQuery_89090_1)

| Alignment statistics for match #8 | | | | |
| --- | --- | --- | --- | --- |
| **Score** | **Expect** | **Identities** | **Gaps** | **Strand** |
| 123 bits(136) | 5e-30 | 76/81(94%) | 0/81(0%) | Plus/Plus |

Query 2583 AGGGATATCAAAGGGGCAAACATACTTGTAGATCCTAATGGTGACATCAAGCTTGCTGAT 2642

||||||||||||||||||||||||||||| ||||||||||||||||||||||||||||||

Sbjct 1247 AGGGATATCAAAGGGGCAAACATACTTGTCGATCCTAATGGTGACATCAAGCTTGCTGAT 1306

Query 2643 TTTGGTATGGCGAAACATGTA 2663

||||| ||||| || ||| ||

Sbjct 1307 TTTGGCATGGCCAAGCATATA 1327

Range 9: 1742 to 1858[Graphics](https://www.ncbi.nlm.nih.gov/projects/sviewer/?RID=SUYPNXCX114&id=lcl|Query_89090&tracks=%5bkey:sequence_track,name:Sequence,display_name:Sequence,id:STD1,category:Sequence,annots:Sequence,ShowLabel:true%5d%5bkey:gene_model_track,CDSProductFeats:false%5d%5bkey:alignment_track,name:other%20alignments,annots:NG%20Alignments|Refseq%20Alignments|Gnomon%20Alignments|Unnamed,shown:false%5d&v=1737:1863&appname=ncbiblast&link_loc=fromHSP)Next MatchPrevious Match[First Match](https://blast.ncbi.nlm.nih.gov/Blast.cgi#hspQuery_89090_1)

| Alignment statistics for match #9 | | | | |
| --- | --- | --- | --- | --- |
| **Score** | **Expect** | **Identities** | **Gaps** | **Strand** |
| 122 bits(134) | 2e-29 | 97/117(83%) | 0/117(0%) | Plus/Plus |

Query 3594 ATTGAAACTTCGTCATACAGAAGTTTATCTCCTTTAAGAGATCCTGATATCCTTGGAAGA 3653

|||| | ||| ||||||||||| |||||||| ||||||||||||||| || | ||||

Sbjct 1742 ATTGCAGTTTCATCATACAGAAGCTTATCTCCATTAAGAGATCCTGATGTCGTGATAAGA 1801

Query 3654 AACTTGCCAGGACCAACATCCCCTATTCCTTCGACATCAAGTCGCAGGATCGCAGCA 3710

|| |||| ||||||||||||||| |||||| ||| |||| ||||||||| || |||

Sbjct 1802 AATTTGCAAGGACCAACATCCCCCATTCCTCCGATGTCAAATCGCAGGATTGCGGCA 1858

Range 10: 1022 to 1087[Graphics](https://www.ncbi.nlm.nih.gov/projects/sviewer/?RID=SUYPNXCX114&id=lcl|Query_89090&tracks=%5bkey:sequence_track,name:Sequence,display_name:Sequence,id:STD1,category:Sequence,annots:Sequence,ShowLabel:true%5d%5bkey:gene_model_track,CDSProductFeats:false%5d%5bkey:alignment_track,name:other%20alignments,annots:NG%20Alignments|Refseq%20Alignments|Gnomon%20Alignments|Unnamed,shown:false%5d&v=1019:1090&appname=ncbiblast&link_loc=fromHSP)Next MatchPrevious Match[First Match](https://blast.ncbi.nlm.nih.gov/Blast.cgi#hspQuery_89090_1)

| Alignment statistics for match #10 | | | | |
| --- | --- | --- | --- | --- |
| **Score** | **Expect** | **Identities** | **Gaps** | **Strand** |
| 105 bits(116) | 1e-24 | 63/66(95%) | 0/66(0%) | Plus/Plus |

Query 2103 CAGGAAATCGTGCTGCTGAGTCAGCTGTCACATCCAAACATTGTTCAGTACTATGGCAGT 2162

||||||||| |||||||||||||||||||||||||||||||||| |||||||||||||||

Sbjct 1022 CAGGAAATCATGCTGCTGAGTCAGCTGTCACATCCAAACATTGTACAGTACTATGGCAGT 1081

Query 2163 GATTTG 2168

||| ||

Sbjct 1082 GATCTG 1087

Range 11: 1322 to 1386[Graphics](https://www.ncbi.nlm.nih.gov/projects/sviewer/?RID=SUYPNXCX114&id=lcl|Query_89090&tracks=%5bkey:sequence_track,name:Sequence,display_name:Sequence,id:STD1,category:Sequence,annots:Sequence,ShowLabel:true%5d%5bkey:gene_model_track,CDSProductFeats:false%5d%5bkey:alignment_track,name:other%20alignments,annots:NG%20Alignments|Refseq%20Alignments|Gnomon%20Alignments|Unnamed,shown:false%5d&v=1319:1389&appname=ncbiblast&link_loc=fromHSP)Next MatchPrevious Match[First Match](https://blast.ncbi.nlm.nih.gov/Blast.cgi#hspQuery_89090_1)

| Alignment statistics for match #11 | | | | |
| --- | --- | --- | --- | --- |
| **Score** | **Expect** | **Identities** | **Gaps** | **Strand** |
| 104 bits(114) | 5e-24 | 62/65(95%) | 0/65(0%) | Plus/Plus |

Query 2757 CAGATATCAGCATATACATCTATCAAATCCTTCAAAGGGAGCCCTTACTGGATGGCACCA 2816

|| ||||||||||| |||||||||| ||||||||||||||||||||||||||||||||||

Sbjct 1322 CATATATCAGCATACACATCTATCAGATCCTTCAAAGGGAGCCCTTACTGGATGGCACCA 1381

Query 2817 GAGGT 2821

|||||

Sbjct 1382 GAGGT 1386

Query**:** GGRMZM6G513881.

Subject: isotig03086 gene=isogroup00379

| Alignment statistics for match #1 | | | | |
| --- | --- | --- | --- | --- |
| **Score** | **Expect** | **Identities** | **Gaps** | **Strand** |
| 610 bits(676) | 1e-176 | 428/488(88%) | 0/488(0%) | Plus/Plus |

Query 1320 AGGTTGTCAGAAACAAGCAGCACTCTTCTGGGCAGAACAGTAGCAATTGAATCCCGGAAA 1379

||||||||||||||||||||||| |||| |||||||||||| || |||||| ||||||

Sbjct 422 AGGTTGTCAGAAACAAGCAGCACACTTCCTGGCAGAACAGTACCAGCTGAATCTCGGAAA 481

Query 1380 CAAAGTCAGGTGCCAGCAGAGGGGACCATTTTCACCAATAATCAGGCTGTGGAGCATACC 1439

|||||||| |||| |||||| ||| |||||| || |||| ||||||| |||| ||| ||

Sbjct 482 CAAAGTCATGTGCTAGCAGAAGGGCGCATTTTTACAAATAGTCAGGCTTTGGACCATTCC 541

Query 1440 CGGTTGTCTGAAACATCAGTTTCCCCAAGGAAAGAATTTCGCCCTCAAAATTTGGATCTT 1499

|| ||||||||||| |||||||| |||||||||||||||| || ||||||| |||||||

Sbjct 542 CGATTGTCTGAAACCTCAGTTTCTCCAAGGAAAGAATTTCACCTTCAAAATCTGGATCTG 601

Query 1500 GCAAATGATCGAACTACATACTGCCGTGGTCGGAGATCAACCGAAATCGTGTTCAGTACA 1559

|||||||||| ||||| ||||| |||||||||| |||||| ||||| ||||||||| ||

Sbjct 602 GCAAATGATCAAACTAGGTACTGTCGTGGTCGGAAATCAACAGAAATTGTGTTCAGTCCA 661

Query 1560 CAAGTGCCCACTTCTCCTCCTAGTTCAAGAGGACATCACTATCAAAATTCGCCTGTGCCA 1619

||||||||| ||||||| |||| ||| |||||||||||||||| || || ||||||||

Sbjct 662 CAAGTGCCCGCTTCTCCACCTAATTCCAGAGGACATCACTATCCAACCTCCCCTGTGCCG 721

Query 1620 TCAAGAACATTTGGGCAATGCCCTGCATCTCCTACTTCATGGCAGGATGATTCGCGAAGC 1679

||||||||||||||||| |||||||||||||||||||||||||||| ||||| ||||||

Sbjct 722 ACAAGAACATTTGGGCAAGGCCCTGCATCTCCTACTTCATGGCAGGAGGATTCCCGAAGC 781

Query 1680 TCAAGCTCACCCCAACCACTTCCTCTTCCTCCAGGTTCCCCATGCTTGCCTTCCTCTTCT 1739

||||||| ||| || || ||||||||||||||||| ||||||||||| |||||| |||

Sbjct 782 TCAAGCTTACCTCAGCCTCTTCCTCTTCCTCCAGGCTCCCCATGCTTACCTTCCCGCTCT 841

Query 1740 CTACAGTGGAAGAAGGGGAAGTTGCTAGGTAGTGGGACGTTTGGGCAAGTATACATGGGA 1799

||||||||||| |||||||||||||| || ||||||||||||||||||||||| |||||

Sbjct 842 CTACAGTGGAAAAAGGGGAAGTTGCTTGGCAGTGGGACGTTTGGGCAAGTATATTTGGGA 901

Query 1800 TTCAACAG 1807

||||||||

Sbjct 902 TTCAACAG 909

Range 2: 1835 to 2352[Graphics](https://www.ncbi.nlm.nih.gov/projects/sviewer/?RID=SUYPNXCX114&id=lcl|Query_89089&tracks=%5bkey:sequence_track,name:Sequence,display_name:Sequence,id:STD1,category:Sequence,annots:Sequence,ShowLabel:true%5d%5bkey:gene_model_track,CDSProductFeats:false%5d%5bkey:alignment_track,name:other%20alignments,annots:NG%20Alignments|Refseq%20Alignments|Gnomon%20Alignments|Unnamed,shown:false%5d&v=1810:2377&appname=ncbiblast&link_loc=fromHSP)Next MatchPrevious Match[First Match](https://blast.ncbi.nlm.nih.gov/Blast.cgi#hspQuery_89089_1)

| Alignment statistics for match #2 | | | | |
| --- | --- | --- | --- | --- |
| **Score** | **Expect** | **Identities** | **Gaps** | **Strand** |
| 585 bits(648) | 5e-169 | 456/543(84%) | 28/543(5%) | Plus/Plus |

Query 3791 AACACATCCAATGTTCGGATGAACATGTCGCTGCCTGTCTCTCCCTGCTCTAGCCCGCTA 3850

||| |||||||| |||||||||||||||| ||||||||||||||||||||||||| |||

Sbjct 1835 AACCCATCCAATATTCGGATGAACATGTCCGTGCCTGTCTCTCCCTGCTCTAGCCCACTA 1894

Query 3851 CGGCAGTACAGGCAGTCCAACCGAAGTTGCTTGCGCTCCCCTCCCCACCCAGCCTATTCA 3910

|||||||| |||||||| || ||||||||||||| || ||||| || ||||||||||||

Sbjct 1895 CGGCAGTATAGGCAGTCGAATCGAAGTTGCTTGCCATCGCCTCCTCATCCAGCCTATTCA 1954

Query 3911 GCTGGAGCAGCCAACTACAATCCTATCAACAATGCACTCTACCCAACGCGACCAAGCAGC 3970

||||||||||||||||||| ||||||||| || ||||||| || | |||||||||||||

Sbjct 1955 GCTGGAGCAGCCAACTACAGTCCTATCAATAACACACTCTATCCGATGCGACCAAGCAGC 2014

Query 3971 GGTCTCACAGATCCATGGCTCGAAATCTCTCAGGTGAAAACGCAAACTTTTGATTCTCCA 4030

||||| ||||| ||||||||||||| || ||| ||||||| ||||||||||||||||||

Sbjct 2015 GGTCTAACAGAGCCATGGCTCGAAAACTTTCAACTGAAAACACAAACTTTTGATTCTCCA 2074

Query 4031 AGAAGATTGTAGAGATT-CCAAAAAGAAGCAATACTTTGTATACAGGCAGGGAAAGAAGT 4089

|||||||| |||||||| ||||||||||| ||||| ||||||| || |||||

Sbjct 2075 AGAAGATTATAGAGATTCCCAAAAAGAAGTAATACATTGTATAGAG---------GAAGT 2125

Query 4090 GTTTCATTATTTATGTTGTTAGAGAAACAAAGGAACACCtttttttttttGTGTTTCGCC 4149

||| ||| ||||||||| ||||| ||||||||||| | | ||||| |||||| ||

Sbjct 2126 GTTCCATAATTTATGTTATTAGAAGAACAAAGGAACTGCCTCTTTTT----TGTTTCACC 2181

Query 4150 CTTTCTGTATGTATCTTTCACCCAGGATGCAGTTGCATCGCCTTTGTACAAATTCAGAGA 4209

|||||||||||||| ||||||||||||||||| |||||||||||||| |||||||||||

Sbjct 2182 CTTTCTGTATGTATTTTTCACCCAGGATGCAGACGCATCGCCTTTGTATAAATTCAGAGA 2241

Query 4210 AGAGCTAGTCAAAAAGAGTAGCATTTCAGATCGCACATCTATATGTTTTGTCCATATGGC 4269

||||||||| |||||||||||||||||| ||||| ||||||||||| |

Sbjct 2242 AGAGCTAGTGAAAAAGAGTAGCATTTCACATCGC-----------CTTTGTCCATAT-TC 2289

Query 4270 TAGATTGTGCACAAG--GTGTTAGAGAAAGGAGATTCATGGTAATTGAATCTGATGCATA 4327

| |||| | | |||| ||| | |||||||||||||||||||||| |||||||||| |

Sbjct 2290 TGGATTTTTCTCAAGATGTGATTCTGAAAGGAGATTCATGGTAATTGGATCTGATGCACA 2349

Query 4328 TGG 4330

|||

Sbjct 2350 TGG 2352

Range 3: 1465 to 1704[Graphics](https://www.ncbi.nlm.nih.gov/projects/sviewer/?RID=SUYPNXCX114&id=lcl|Query_89089&tracks=%5bkey:sequence_track,name:Sequence,display_name:Sequence,id:STD1,category:Sequence,annots:Sequence,ShowLabel:true%5d%5bkey:gene_model_track,CDSProductFeats:false%5d%5bkey:alignment_track,name:other%20alignments,annots:NG%20Alignments|Refseq%20Alignments|Gnomon%20Alignments|Unnamed,shown:false%5d&v=1454:1715&appname=ncbiblast&link_loc=fromHSP)Next MatchPrevious Match[First Match](https://blast.ncbi.nlm.nih.gov/Blast.cgi#hspQuery_89089_1)

| Alignment statistics for match #3 | | | | |
| --- | --- | --- | --- | --- |
| **Score** | **Expect** | **Identities** | **Gaps** | **Strand** |
| 320 bits(354) | 3e-89 | 215/240(90%) | 0/240(0%) | Plus/Plus |

Query 3121 GGTGGCAGCAATATTCAAAATTGGCAACAGCAAAGACATACCAGATATCCCAAATAATCT 3180

|||||| |||||||| || ||||| ||||||||||||||||| ||||||||| || ||||

Sbjct 1465 GGTGGCTGCAATATTTAAGATTGGAAACAGCAAAGACATACCTGATATCCCAGATCATCT 1524

Query 3181 TTCTTCTGAGGCAAAAAGTTTCCTGAAACTCTGCTTGCAGCGTGATCCTGCTGCCCGCCC 3240

|||| ||||||| ||||| || || ||||| || ||||||||||||||||||||||| ||

Sbjct 1525 TTCTCCTGAGGCGAAAAGCTTTCTTAAACTATGTTTGCAGCGTGATCCTGCTGCCCGGCC 1584

Query 3241 TACAGCTGCTCAGCTGATGGATCACCCTTTTGTCAAGGACCAGGCTACAGTCAGGAGTTC 3300

||| ||||||||| |||||||||||||||||||||||||||| |||||||| ||||||||

Sbjct 1585 TACTGCTGCTCAGTTGATGGATCACCCTTTTGTCAAGGACCATGCTACAGTTAGGAGTTC 1644

Query 3301 CAGGTCCAGTATCACAAGGGATATGTTTCCTAATTCAACTGACGGAAAAAACAGCAGGGT 3360

||||||||| | | |||||||||||||||||| ||||||||| ||||||||||||| |||

Sbjct 1645 CAGGTCCAGCACCCCAAGGGATATGTTTCCTACTTCAACTGATGGAAAAAACAGCATGGT 1704

Range 4: 146 to 425[Graphics](https://www.ncbi.nlm.nih.gov/projects/sviewer/?RID=SUYPNXCX114&id=lcl|Query_89089&tracks=%5bkey:sequence_track,name:Sequence,display_name:Sequence,id:STD1,category:Sequence,annots:Sequence,ShowLabel:true%5d%5bkey:gene_model_track,CDSProductFeats:false%5d%5bkey:alignment_track,name:other%20alignments,annots:NG%20Alignments|Refseq%20Alignments|Gnomon%20Alignments|Unnamed,shown:false%5d&v=133:438&appname=ncbiblast&link_loc=fromHSP)Next MatchPrevious Match[First Match](https://blast.ncbi.nlm.nih.gov/Blast.cgi#hspQuery_89089_1)

| Alignment statistics for match #4 | | | | |
| --- | --- | --- | --- | --- |
| **Score** | **Expect** | **Identities** | **Gaps** | **Strand** |
| 297 bits(328) | 4e-82 | 242/291(83%) | 16/291(5%) | Plus/Plus |

Query 1 GCCGCTGCCATCGGCGATGTGAAGGAGGAGAAGGGGAAGAAGAAGGCGAGCAGCTTCGAC 60

||||| ||| ||| || || ||||||| |||||||||||||||||||||||||||||||

Sbjct 146 GCCGCCGCCGCCGGGGAGGTTAAGGAGGCGAAGGGGAAGAAGAAGGCGAGCAGCTTCGAC 205

Query 61 GAGGCGCTCCTCGCCAAGGGCGTCCGCGGGAAGCAGCAGCATGCGCCGGCGGCGGCGG-- 118

|||||||| ||||||||||| |||||||||||||||||||| |||| |||||||||

Sbjct 206 GAGGCGCTGCTCGCCAAGGGAGTCCGCGGGAAGCAGCAGCAGCAGCCGCCGGCGGCGGCT 265

Query 119 GGGAGGGTATGGGGCTCCCGCTCCCGCTCCCGCGCCCGGCGTCCTTGCCGACGCCGCTGC 178

| ||| || || ||||||||||||||| ||||||||||||||| |||||||||

Sbjct 266 GCCTCGGT-TG----TCGGGCTCCCGCTCCCGCGGCCGGCGTCCTTGCCGGCGCCGCTGC 320

Query 179 CGTCCGCGTCCGCGTCGGCCTCCGCCTCGGCGTCGGCGTCCAGCGGCGGCGACTCCTCGC 238

|||| |||||||| ||||| |||||| |||| |||||||||||||| |||||||

Sbjct 321 CGTCTGCGTCCGCCTCGGCATCCGCC------TCGGGGTCCAGCGGCGGCGGATCCTCGC 374

Query 239 TGGGGTCCT---CCACGTCCGACGACCAGCTGGATCTCGGGGTTTACAGGT 286

||| ||||| | |||||||||| ||||||||| |||| ||||||||||

Sbjct 375 TGGTGTCCTCGGCGGCGTCCGACGAGCAGCTGGATTTCGGTGTTTACAGGT 425

Range 5: 1064 to 1225[Graphics](https://www.ncbi.nlm.nih.gov/projects/sviewer/?RID=SUYPNXCX114&id=lcl|Query_89089&tracks=%5bkey:sequence_track,name:Sequence,display_name:Sequence,id:STD1,category:Sequence,annots:Sequence,ShowLabel:true%5d%5bkey:gene_model_track,CDSProductFeats:false%5d%5bkey:alignment_track,name:other%20alignments,annots:NG%20Alignments|Refseq%20Alignments|Gnomon%20Alignments|Unnamed,shown:false%5d&v=1056:1233&appname=ncbiblast&link_loc=fromHSP)Next MatchPrevious Match[First Match](https://blast.ncbi.nlm.nih.gov/Blast.cgi#hspQuery_89089_1)

| Alignment statistics for match #5 | | | | |
| --- | --- | --- | --- | --- |
| **Score** | **Expect** | **Identities** | **Gaps** | **Strand** |
| 257 bits(284) | 3e-70 | 154/162(95%) | 0/162(0%) | Plus/Plus |

Query 2311 AATGAGACACTCTCGGTCTATCTCGAGTATGTTTCTGGGGGCTCTATCCATAAGTTGCTT 2370

||||||||||||||||||||||||||||| |||||||||||||| |||||||||||| ||

Sbjct 1064 AATGAGACACTCTCGGTCTATCTCGAGTACGTTTCTGGGGGCTCCATCCATAAGTTGATT 1123

Query 2371 CAAGAATATGGTCCGTTTGGGGAGGCAGTGCTTCGGAATTACACAGCACAAATCCTTTCT 2430

||||||||||||||||||||||||||||| |||||||||||||| || ||||||||||||

Sbjct 1124 CAAGAATATGGTCCGTTTGGGGAGGCAGTTCTTCGGAATTACACTGCGCAAATCCTTTCT 1183

Query 2431 GGCCTTGCATACTTGCATGGGCGGAATACAGTGCATAGGTAT 2472

|| |||||||||||||||||||||||||||||||||||| ||

Sbjct 1184 GGTCTTGCATACTTGCATGGGCGGAATACAGTGCATAGGGAT 1225

Range 6: 1356 to 1468[Graphics](https://www.ncbi.nlm.nih.gov/projects/sviewer/?RID=SUYPNXCX114&id=lcl|Query_89089&tracks=%5bkey:sequence_track,name:Sequence,display_name:Sequence,id:STD1,category:Sequence,annots:Sequence,ShowLabel:true%5d%5bkey:gene_model_track,CDSProductFeats:false%5d%5bkey:alignment_track,name:other%20alignments,annots:NG%20Alignments|Refseq%20Alignments|Gnomon%20Alignments|Unnamed,shown:false%5d&v=1351:1473&appname=ncbiblast&link_loc=fromHSP)Next MatchPrevious Match[First Match](https://blast.ncbi.nlm.nih.gov/Blast.cgi#hspQuery_89089_1)

| Alignment statistics for match #6 | | | | |
| --- | --- | --- | --- | --- |
| **Score** | **Expect** | **Identities** | **Gaps** | **Strand** |
| 196 bits(216) | 1e-51 | 111/113(98%) | 0/113(0%) | Plus/Plus |

Query 2910 AGGTTATTATGAATAGCAATGGTTACAGCCTTTCAGTAGACATTTGGAGCCTTGGCTGCA 2969

||||||| ||||||||||||||||||||||||||||||||||||||||||||||||||||

Sbjct 1356 AGGTTATCATGAATAGCAATGGTTACAGCCTTTCAGTAGACATTTGGAGCCTTGGCTGCA 1415

Query 2970 CCATTCTTGAGATGGCAACAGCAAAGCCTCCTTGGAGTCAGTATGAAGGGGTG 3022

||||||||||||||||||||||||| |||||||||||||||||||||||||||

Sbjct 1416 CCATTCTTGAGATGGCAACAGCAAAACCTCCTTGGAGTCAGTATGAAGGGGTG 1468

Range 7: 907 to 998[Graphics](https://www.ncbi.nlm.nih.gov/projects/sviewer/?RID=SUYPNXCX114&id=lcl|Query_89089&tracks=%5bkey:sequence_track,name:Sequence,display_name:Sequence,id:STD1,category:Sequence,annots:Sequence,ShowLabel:true%5d%5bkey:gene_model_track,CDSProductFeats:false%5d%5bkey:alignment_track,name:other%20alignments,annots:NG%20Alignments|Refseq%20Alignments|Gnomon%20Alignments|Unnamed,shown:false%5d&v=903:1002&appname=ncbiblast&link_loc=fromHSP)Next MatchPrevious Match[First Match](https://blast.ncbi.nlm.nih.gov/Blast.cgi#hspQuery_89089_1)

| Alignment statistics for match #7 | | | | |
| --- | --- | --- | --- | --- |
| **Score** | **Expect** | **Identities** | **Gaps** | **Strand** |
| 152 bits(168) | 1e-38 | 89/92(97%) | 0/92(0%) | Plus/Plus |

Query 1919 CAGTGAAGGTGGTCAAATGTGTGCAATTAAAGAGGTTAAGGTCATTTCAGATGATTCTAA 1978

|||||||||||||||||||||||||||||||||||||||||||||||| |||||||||||

Sbjct 907 CAGTGAAGGTGGTCAAATGTGTGCAATTAAAGAGGTTAAGGTCATTTCTGATGATTCTAA 966

Query 1979 CTCAAAGGAGTCCCTCAGGCAGCTAAATCAGG 2010

|||||| |||| ||||||||||||||||||||

Sbjct 967 CTCAAAAGAGTGCCTCAGGCAGCTAAATCAGG 998

Range 8: 1220 to 1300[Graphics](https://www.ncbi.nlm.nih.gov/projects/sviewer/?RID=SUYPNXCX114&id=lcl|Query_89089&tracks=%5bkey:sequence_track,name:Sequence,display_name:Sequence,id:STD1,category:Sequence,annots:Sequence,ShowLabel:true%5d%5bkey:gene_model_track,CDSProductFeats:false%5d%5bkey:alignment_track,name:other%20alignments,annots:NG%20Alignments|Refseq%20Alignments|Gnomon%20Alignments|Unnamed,shown:false%5d&v=1216:1304&appname=ncbiblast&link_loc=fromHSP)Next MatchPrevious Match[First Match](https://blast.ncbi.nlm.nih.gov/Blast.cgi#hspQuery_89089_1)

| Alignment statistics for match #8 | | | | |
| --- | --- | --- | --- | --- |
| **Score** | **Expect** | **Identities** | **Gaps** | **Strand** |
| 123 bits(136) | 5e-30 | 76/81(94%) | 0/81(0%) | Plus/Plus |

Query 2583 AGGGATATCAAAGGGGCAAACATACTTGTAGATCCTAATGGTGACATCAAGCTTGCTGAT 2642

||||||||||||||||||||||||||||| ||||||||||||||||||||||||||||||

Sbjct 1220 AGGGATATCAAAGGGGCAAACATACTTGTCGATCCTAATGGTGACATCAAGCTTGCTGAT 1279

Query 2643 TTTGGTATGGCGAAACATGTA 2663

||||| ||||| || ||| ||

Sbjct 1280 TTTGGCATGGCCAAGCATATA 1300

Range 9: 1715 to 1831[Graphics](https://www.ncbi.nlm.nih.gov/projects/sviewer/?RID=SUYPNXCX114&id=lcl|Query_89089&tracks=%5bkey:sequence_track,name:Sequence,display_name:Sequence,id:STD1,category:Sequence,annots:Sequence,ShowLabel:true%5d%5bkey:gene_model_track,CDSProductFeats:false%5d%5bkey:alignment_track,name:other%20alignments,annots:NG%20Alignments|Refseq%20Alignments|Gnomon%20Alignments|Unnamed,shown:false%5d&v=1710:1836&appname=ncbiblast&link_loc=fromHSP)Next MatchPrevious Match[First Match](https://blast.ncbi.nlm.nih.gov/Blast.cgi#hspQuery_89089_1)

| Alignment statistics for match #9 | | | | |
| --- | --- | --- | --- | --- |
| **Score** | **Expect** | **Identities** | **Gaps** | **Strand** |
| 122 bits(134) | 2e-29 | 97/117(83%) | 0/117(0%) | Plus/Plus |

Query 3594 ATTGAAACTTCGTCATACAGAAGTTTATCTCCTTTAAGAGATCCTGATATCCTTGGAAGA 3653

|||| | ||| ||||||||||| |||||||| ||||||||||||||| || | ||||

Sbjct 1715 ATTGCAGTTTCATCATACAGAAGCTTATCTCCATTAAGAGATCCTGATGTCGTGATAAGA 1774

Query 3654 AACTTGCCAGGACCAACATCCCCTATTCCTTCGACATCAAGTCGCAGGATCGCAGCA 3710

|| |||| ||||||||||||||| |||||| ||| |||| ||||||||| || |||

Sbjct 1775 AATTTGCAAGGACCAACATCCCCCATTCCTCCGATGTCAAATCGCAGGATTGCGGCA 1831

Range 10: 995 to 1060[Graphics](https://www.ncbi.nlm.nih.gov/projects/sviewer/?RID=SUYPNXCX114&id=lcl|Query_89089&tracks=%5bkey:sequence_track,name:Sequence,display_name:Sequence,id:STD1,category:Sequence,annots:Sequence,ShowLabel:true%5d%5bkey:gene_model_track,CDSProductFeats:false%5d%5bkey:alignment_track,name:other%20alignments,annots:NG%20Alignments|Refseq%20Alignments|Gnomon%20Alignments|Unnamed,shown:false%5d&v=992:1063&appname=ncbiblast&link_loc=fromHSP)Next MatchPrevious Match[First Match](https://blast.ncbi.nlm.nih.gov/Blast.cgi#hspQuery_89089_1)

| Alignment statistics for match #10 | | | | |
| --- | --- | --- | --- | --- |
| **Score** | **Expect** | **Identities** | **Gaps** | **Strand** |
| 105 bits(116) | 1e-24 | 63/66(95%) | 0/66(0%) | Plus/Plus |

Query 2103 CAGGAAATCGTGCTGCTGAGTCAGCTGTCACATCCAAACATTGTTCAGTACTATGGCAGT 2162

||||||||| |||||||||||||||||||||||||||||||||| |||||||||||||||

Sbjct 995 CAGGAAATCATGCTGCTGAGTCAGCTGTCACATCCAAACATTGTACAGTACTATGGCAGT 1054

Query 2163 GATTTG 2168

||| ||

Sbjct 1055 GATCTG 1060

Range 11: 1295 to 1359[Graphics](https://www.ncbi.nlm.nih.gov/projects/sviewer/?RID=SUYPNXCX114&id=lcl|Query_89089&tracks=%5bkey:sequence_track,name:Sequence,display_name:Sequence,id:STD1,category:Sequence,annots:Sequence,ShowLabel:true%5d%5bkey:gene_model_track,CDSProductFeats:false%5d%5bkey:alignment_track,name:other%20alignments,annots:NG%20Alignments|Refseq%20Alignments|Gnomon%20Alignments|Unnamed,shown:false%5d&v=1292:1362&appname=ncbiblast&link_loc=fromHSP)Next MatchPrevious Match[First Match](https://blast.ncbi.nlm.nih.gov/Blast.cgi#hspQuery_89089_1)

| Alignment statistics for match #11 | | | | |
| --- | --- | --- | --- | --- |
| **Score** | **Expect** | **Identities** | **Gaps** | **Strand** |
| 104 bits(114) | 5e-24 | 62/65(95%) | 0/65(0%) | Plus/Plus |

Query 2757 CAGATATCAGCATATACATCTATCAAATCCTTCAAAGGGAGCCCTTACTGGATGGCACCA 2816

|| ||||||||||| |||||||||| ||||||||||||||||||||||||||||||||||

Sbjct 1295 CATATATCAGCATACACATCTATCAGATCCTTCAAAGGGAGCCCTTACTGGATGGCACCA 1354

Query 2817 GAGGT 2821

|||||

Sbjct 1355 GAGGT 1359

Query: GGRMZM6G513881.

Subject: isotig03085 gene=isogroup00379

| Alignment statistics for match #1 | | | | |
| --- | --- | --- | --- | --- |
| **Score** | **Expect** | **Identities** | **Gaps** | **Strand** |
| 610 bits(676) | 1e-176 | 428/488(88%) | 0/488(0%) | Plus/Plus |

Query 1320 AGGTTGTCAGAAACAAGCAGCACTCTTCTGGGCAGAACAGTAGCAATTGAATCCCGGAAA 1379

||||||||||||||||||||||| |||| |||||||||||| || |||||| ||||||

Sbjct 447 AGGTTGTCAGAAACAAGCAGCACACTTCCTGGCAGAACAGTACCAGCTGAATCTCGGAAA 506

Query 1380 CAAAGTCAGGTGCCAGCAGAGGGGACCATTTTCACCAATAATCAGGCTGTGGAGCATACC 1439

|||||||| |||| |||||| ||| |||||| || |||| ||||||| |||| ||| ||

Sbjct 507 CAAAGTCATGTGCTAGCAGAAGGGCGCATTTTTACAAATAGTCAGGCTTTGGACCATTCC 566

Query 1440 CGGTTGTCTGAAACATCAGTTTCCCCAAGGAAAGAATTTCGCCCTCAAAATTTGGATCTT 1499

|| ||||||||||| |||||||| |||||||||||||||| || ||||||| |||||||

Sbjct 567 CGATTGTCTGAAACCTCAGTTTCTCCAAGGAAAGAATTTCACCTTCAAAATCTGGATCTG 626

Query 1500 GCAAATGATCGAACTACATACTGCCGTGGTCGGAGATCAACCGAAATCGTGTTCAGTACA 1559

|||||||||| ||||| ||||| |||||||||| |||||| ||||| ||||||||| ||

Sbjct 627 GCAAATGATCAAACTAGGTACTGTCGTGGTCGGAAATCAACAGAAATTGTGTTCAGTCCA 686

Query 1560 CAAGTGCCCACTTCTCCTCCTAGTTCAAGAGGACATCACTATCAAAATTCGCCTGTGCCA 1619

||||||||| ||||||| |||| ||| |||||||||||||||| || || ||||||||

Sbjct 687 CAAGTGCCCGCTTCTCCACCTAATTCCAGAGGACATCACTATCCAACCTCCCCTGTGCCG 746

Query 1620 TCAAGAACATTTGGGCAATGCCCTGCATCTCCTACTTCATGGCAGGATGATTCGCGAAGC 1679

||||||||||||||||| |||||||||||||||||||||||||||| ||||| ||||||

Sbjct 747 ACAAGAACATTTGGGCAAGGCCCTGCATCTCCTACTTCATGGCAGGAGGATTCCCGAAGC 806

Query 1680 TCAAGCTCACCCCAACCACTTCCTCTTCCTCCAGGTTCCCCATGCTTGCCTTCCTCTTCT 1739

||||||| ||| || || ||||||||||||||||| ||||||||||| |||||| |||

Sbjct 807 TCAAGCTTACCTCAGCCTCTTCCTCTTCCTCCAGGCTCCCCATGCTTACCTTCCCGCTCT 866

Query 1740 CTACAGTGGAAGAAGGGGAAGTTGCTAGGTAGTGGGACGTTTGGGCAAGTATACATGGGA 1799

||||||||||| |||||||||||||| || ||||||||||||||||||||||| |||||

Sbjct 867 CTACAGTGGAAAAAGGGGAAGTTGCTTGGCAGTGGGACGTTTGGGCAAGTATATTTGGGA 926

Query 1800 TTCAACAG 1807

||||||||

Sbjct 927 TTCAACAG 934

Range 2: 1860 to 2377[Graphics](https://www.ncbi.nlm.nih.gov/projects/sviewer/?RID=SUYPNXCX114&id=lcl|Query_89088&tracks=%5bkey:sequence_track,name:Sequence,display_name:Sequence,id:STD1,category:Sequence,annots:Sequence,ShowLabel:true%5d%5bkey:gene_model_track,CDSProductFeats:false%5d%5bkey:alignment_track,name:other%20alignments,annots:NG%20Alignments|Refseq%20Alignments|Gnomon%20Alignments|Unnamed,shown:false%5d&v=1835:2402&appname=ncbiblast&link_loc=fromHSP)Next MatchPrevious Match[First Match](https://blast.ncbi.nlm.nih.gov/Blast.cgi#hspQuery_89088_1)

| Alignment statistics for match #2 | | | | |
| --- | --- | --- | --- | --- |
| **Score** | **Expect** | **Identities** | **Gaps** | **Strand** |
| 585 bits(648) | 5e-169 | 456/543(84%) | 28/543(5%) | Plus/Plus |

Query 3791 AACACATCCAATGTTCGGATGAACATGTCGCTGCCTGTCTCTCCCTGCTCTAGCCCGCTA 3850

||| |||||||| |||||||||||||||| ||||||||||||||||||||||||| |||

Sbjct 1860 AACCCATCCAATATTCGGATGAACATGTCCGTGCCTGTCTCTCCCTGCTCTAGCCCACTA 1919

Query 3851 CGGCAGTACAGGCAGTCCAACCGAAGTTGCTTGCGCTCCCCTCCCCACCCAGCCTATTCA 3910

|||||||| |||||||| || ||||||||||||| || ||||| || ||||||||||||

Sbjct 1920 CGGCAGTATAGGCAGTCGAATCGAAGTTGCTTGCCATCGCCTCCTCATCCAGCCTATTCA 1979

Query 3911 GCTGGAGCAGCCAACTACAATCCTATCAACAATGCACTCTACCCAACGCGACCAAGCAGC 3970

||||||||||||||||||| ||||||||| || ||||||| || | |||||||||||||

Sbjct 1980 GCTGGAGCAGCCAACTACAGTCCTATCAATAACACACTCTATCCGATGCGACCAAGCAGC 2039

Query 3971 GGTCTCACAGATCCATGGCTCGAAATCTCTCAGGTGAAAACGCAAACTTTTGATTCTCCA 4030

||||| ||||| ||||||||||||| || ||| ||||||| ||||||||||||||||||

Sbjct 2040 GGTCTAACAGAGCCATGGCTCGAAAACTTTCAACTGAAAACACAAACTTTTGATTCTCCA 2099

Query 4031 AGAAGATTGTAGAGATT-CCAAAAAGAAGCAATACTTTGTATACAGGCAGGGAAAGAAGT 4089

|||||||| |||||||| ||||||||||| ||||| ||||||| || |||||

Sbjct 2100 AGAAGATTATAGAGATTCCCAAAAAGAAGTAATACATTGTATAGAG---------GAAGT 2150

Query 4090 GTTTCATTATTTATGTTGTTAGAGAAACAAAGGAACACCtttttttttttGTGTTTCGCC 4149

||| ||| ||||||||| ||||| ||||||||||| | | ||||| |||||| ||

Sbjct 2151 GTTCCATAATTTATGTTATTAGAAGAACAAAGGAACTGCCTCTTTTT----TGTTTCACC 2206

Query 4150 CTTTCTGTATGTATCTTTCACCCAGGATGCAGTTGCATCGCCTTTGTACAAATTCAGAGA 4209

|||||||||||||| ||||||||||||||||| |||||||||||||| |||||||||||

Sbjct 2207 CTTTCTGTATGTATTTTTCACCCAGGATGCAGACGCATCGCCTTTGTATAAATTCAGAGA 2266

Query 4210 AGAGCTAGTCAAAAAGAGTAGCATTTCAGATCGCACATCTATATGTTTTGTCCATATGGC 4269

||||||||| |||||||||||||||||| ||||| ||||||||||| |

Sbjct 2267 AGAGCTAGTGAAAAAGAGTAGCATTTCACATCGC-----------CTTTGTCCATAT-TC 2314

Query 4270 TAGATTGTGCACAAG--GTGTTAGAGAAAGGAGATTCATGGTAATTGAATCTGATGCATA 4327

| |||| | | |||| ||| | |||||||||||||||||||||| |||||||||| |

Sbjct 2315 TGGATTTTTCTCAAGATGTGATTCTGAAAGGAGATTCATGGTAATTGGATCTGATGCACA 2374

Query 4328 TGG 4330

|||

Sbjct 2375 TGG 2377

Range 3: 1490 to 1729[Graphics](https://www.ncbi.nlm.nih.gov/projects/sviewer/?RID=SUYPNXCX114&id=lcl|Query_89088&tracks=%5bkey:sequence_track,name:Sequence,display_name:Sequence,id:STD1,category:Sequence,annots:Sequence,ShowLabel:true%5d%5bkey:gene_model_track,CDSProductFeats:false%5d%5bkey:alignment_track,name:other%20alignments,annots:NG%20Alignments|Refseq%20Alignments|Gnomon%20Alignments|Unnamed,shown:false%5d&v=1479:1740&appname=ncbiblast&link_loc=fromHSP)Next MatchPrevious Match[First Match](https://blast.ncbi.nlm.nih.gov/Blast.cgi#hspQuery_89088_1)

| Alignment statistics for match #3 | | | | |
| --- | --- | --- | --- | --- |
| **Score** | **Expect** | **Identities** | **Gaps** | **Strand** |
| 320 bits(354) | 3e-89 | 215/240(90%) | 0/240(0%) | Plus/Plus |

Query 3121 GGTGGCAGCAATATTCAAAATTGGCAACAGCAAAGACATACCAGATATCCCAAATAATCT 3180

|||||| |||||||| || ||||| ||||||||||||||||| ||||||||| || ||||

Sbjct 1490 GGTGGCTGCAATATTTAAGATTGGAAACAGCAAAGACATACCTGATATCCCAGATCATCT 1549

Query 3181 TTCTTCTGAGGCAAAAAGTTTCCTGAAACTCTGCTTGCAGCGTGATCCTGCTGCCCGCCC 3240

|||| ||||||| ||||| || || ||||| || ||||||||||||||||||||||| ||

Sbjct 1550 TTCTCCTGAGGCGAAAAGCTTTCTTAAACTATGTTTGCAGCGTGATCCTGCTGCCCGGCC 1609

Query 3241 TACAGCTGCTCAGCTGATGGATCACCCTTTTGTCAAGGACCAGGCTACAGTCAGGAGTTC 3300

||| ||||||||| |||||||||||||||||||||||||||| |||||||| ||||||||

Sbjct 1610 TACTGCTGCTCAGTTGATGGATCACCCTTTTGTCAAGGACCATGCTACAGTTAGGAGTTC 1669

Query 3301 CAGGTCCAGTATCACAAGGGATATGTTTCCTAATTCAACTGACGGAAAAAACAGCAGGGT 3360

||||||||| | | |||||||||||||||||| ||||||||| ||||||||||||| |||

Sbjct 1670 CAGGTCCAGCACCCCAAGGGATATGTTTCCTACTTCAACTGATGGAAAAAACAGCATGGT 1729

Range 4: 172 to 450[Graphics](https://www.ncbi.nlm.nih.gov/projects/sviewer/?RID=SUYPNXCX114&id=lcl|Query_89088&tracks=%5bkey:sequence_track,name:Sequence,display_name:Sequence,id:STD1,category:Sequence,annots:Sequence,ShowLabel:true%5d%5bkey:gene_model_track,CDSProductFeats:false%5d%5bkey:alignment_track,name:other%20alignments,annots:NG%20Alignments|Refseq%20Alignments|Gnomon%20Alignments|Unnamed,shown:false%5d&v=159:463&appname=ncbiblast&link_loc=fromHSP)Next MatchPrevious Match[First Match](https://blast.ncbi.nlm.nih.gov/Blast.cgi#hspQuery_89088_1)

| Alignment statistics for match #4 | | | | |
| --- | --- | --- | --- | --- |
| **Score** | **Expect** | **Identities** | **Gaps** | **Strand** |
| 291 bits(322) | 2e-80 | 238/288(83%) | 12/288(4%) | Plus/Plus |

Query 2 CCGCTGCCATCGGCGATGTGAAGGAGGAGAAGGGGAAGAAGAAGGCGAGCAGCTTCGACG 61

|||| ||| ||| || || ||||||| ||||||||||||||||||||||||||||||||

Sbjct 172 CCGCAGCCGCCGGGGAGGTTAAGGAGGCGAAGGGGAAGAAGAAGGCGAGCAGCTTCGACG 231

Query 62 AGGCGCTCCTCGCCAAGGGCGTCCGCGGGAAGCAGCAGCATGCGCCGGCGGCGGCGGGGG 121

||||||| ||||||||||| |||||||||||||||||||| |||| |||| |||| |

Sbjct 232 AGGCGCTGCTCGCCAAGGGAGTCCGCGGGAAGCAGCAGCAGCAGCCGCCGGCAGCGGCCG 291

Query 122 AGGGTATGGGGCTCCCGCTCCCGCTCCCGCGCCCGGCGTCCTTGCCGACGCCGCTGCCGT 181

| | | | || |||||||||||||| ||||||||||||||| ||||||||||||

Sbjct 292 CCGCTGTTG---TCGGCCTCCCGCTCCCGCGGCCGGCGTCCTTGCCGGCGCCGCTGCCGT 348

Query 182 CCGCGTCCGCGTCGGCCTCCGCCTCGGCGTCGGCGTCCAGCGGCGGCGACTCCTCGCTGG 241

| |||||||| ||||| |||||| |||| |||||||||||||| ||||||||||

Sbjct 349 CTGCGTCCGCCTCGGCATCCGCC------TCGGGGTCCAGCGGCGGCGGATCCTCGCTGG 402

Query 242 GGTCCT---CCACGTCCGACGACCAGCTGGATCTCGGGGTTTACAGGT 286

||||| | |||||||||| ||||||||| |||| ||||||||||

Sbjct 403 TGTCCTCGGCGGCGTCCGACGAGCAGCTGGATTTCGGTGTTTACAGGT 450

Range 5: 1089 to 1250[Graphics](https://www.ncbi.nlm.nih.gov/projects/sviewer/?RID=SUYPNXCX114&id=lcl|Query_89088&tracks=%5bkey:sequence_track,name:Sequence,display_name:Sequence,id:STD1,category:Sequence,annots:Sequence,ShowLabel:true%5d%5bkey:gene_model_track,CDSProductFeats:false%5d%5bkey:alignment_track,name:other%20alignments,annots:NG%20Alignments|Refseq%20Alignments|Gnomon%20Alignments|Unnamed,shown:false%5d&v=1081:1258&appname=ncbiblast&link_loc=fromHSP)Next MatchPrevious Match[First Match](https://blast.ncbi.nlm.nih.gov/Blast.cgi#hspQuery_89088_1)

| Alignment statistics for match #5 | | | | |
| --- | --- | --- | --- | --- |
| **Score** | **Expect** | **Identities** | **Gaps** | **Strand** |
| 257 bits(284) | 3e-70 | 154/162(95%) | 0/162(0%) | Plus/Plus |

Query 2311 AATGAGACACTCTCGGTCTATCTCGAGTATGTTTCTGGGGGCTCTATCCATAAGTTGCTT 2370

||||||||||||||||||||||||||||| |||||||||||||| |||||||||||| ||

Sbjct 1089 AATGAGACACTCTCGGTCTATCTCGAGTACGTTTCTGGGGGCTCCATCCATAAGTTGATT 1148

Query 2371 CAAGAATATGGTCCGTTTGGGGAGGCAGTGCTTCGGAATTACACAGCACAAATCCTTTCT 2430

||||||||||||||||||||||||||||| |||||||||||||| || ||||||||||||

Sbjct 1149 CAAGAATATGGTCCGTTTGGGGAGGCAGTTCTTCGGAATTACACTGCGCAAATCCTTTCT 1208

Query 2431 GGCCTTGCATACTTGCATGGGCGGAATACAGTGCATAGGTAT 2472

|| |||||||||||||||||||||||||||||||||||| ||

Sbjct 1209 GGTCTTGCATACTTGCATGGGCGGAATACAGTGCATAGGGAT 1250

Range 6: 1381 to 1493[Graphics](https://www.ncbi.nlm.nih.gov/projects/sviewer/?RID=SUYPNXCX114&id=lcl|Query_89088&tracks=%5bkey:sequence_track,name:Sequence,display_name:Sequence,id:STD1,category:Sequence,annots:Sequence,ShowLabel:true%5d%5bkey:gene_model_track,CDSProductFeats:false%5d%5bkey:alignment_track,name:other%20alignments,annots:NG%20Alignments|Refseq%20Alignments|Gnomon%20Alignments|Unnamed,shown:false%5d&v=1376:1498&appname=ncbiblast&link_loc=fromHSP)Next MatchPrevious Match[First Match](https://blast.ncbi.nlm.nih.gov/Blast.cgi#hspQuery_89088_1)

| Alignment statistics for match #6 | | | | |
| --- | --- | --- | --- | --- |
| **Score** | **Expect** | **Identities** | **Gaps** | **Strand** |
| 196 bits(216) | 1e-51 | 111/113(98%) | 0/113(0%) | Plus/Plus |

Query 2910 AGGTTATTATGAATAGCAATGGTTACAGCCTTTCAGTAGACATTTGGAGCCTTGGCTGCA 2969

||||||| ||||||||||||||||||||||||||||||||||||||||||||||||||||

Sbjct 1381 AGGTTATCATGAATAGCAATGGTTACAGCCTTTCAGTAGACATTTGGAGCCTTGGCTGCA 1440

Query 2970 CCATTCTTGAGATGGCAACAGCAAAGCCTCCTTGGAGTCAGTATGAAGGGGTG 3022

||||||||||||||||||||||||| |||||||||||||||||||||||||||

Sbjct 1441 CCATTCTTGAGATGGCAACAGCAAAACCTCCTTGGAGTCAGTATGAAGGGGTG 1493

Range 7: 932 to 1023[Graphics](https://www.ncbi.nlm.nih.gov/projects/sviewer/?RID=SUYPNXCX114&id=lcl|Query_89088&tracks=%5bkey:sequence_track,name:Sequence,display_name:Sequence,id:STD1,category:Sequence,annots:Sequence,ShowLabel:true%5d%5bkey:gene_model_track,CDSProductFeats:false%5d%5bkey:alignment_track,name:other%20alignments,annots:NG%20Alignments|Refseq%20Alignments|Gnomon%20Alignments|Unnamed,shown:false%5d&v=928:1027&appname=ncbiblast&link_loc=fromHSP)Next MatchPrevious Match[First Match](https://blast.ncbi.nlm.nih.gov/Blast.cgi#hspQuery_89088_1)

| Alignment statistics for match #7 | | | | |
| --- | --- | --- | --- | --- |
| **Score** | **Expect** | **Identities** | **Gaps** | **Strand** |
| 152 bits(168) | 1e-38 | 89/92(97%) | 0/92(0%) | Plus/Plus |

Query 1919 CAGTGAAGGTGGTCAAATGTGTGCAATTAAAGAGGTTAAGGTCATTTCAGATGATTCTAA 1978

|||||||||||||||||||||||||||||||||||||||||||||||| |||||||||||

Sbjct 932 CAGTGAAGGTGGTCAAATGTGTGCAATTAAAGAGGTTAAGGTCATTTCTGATGATTCTAA 991

Query 1979 CTCAAAGGAGTCCCTCAGGCAGCTAAATCAGG 2010

|||||| |||| ||||||||||||||||||||

Sbjct 992 CTCAAAAGAGTGCCTCAGGCAGCTAAATCAGG 1023

Range 8: 1245 to 1325[Graphics](https://www.ncbi.nlm.nih.gov/projects/sviewer/?RID=SUYPNXCX114&id=lcl|Query_89088&tracks=%5bkey:sequence_track,name:Sequence,display_name:Sequence,id:STD1,category:Sequence,annots:Sequence,ShowLabel:true%5d%5bkey:gene_model_track,CDSProductFeats:false%5d%5bkey:alignment_track,name:other%20alignments,annots:NG%20Alignments|Refseq%20Alignments|Gnomon%20Alignments|Unnamed,shown:false%5d&v=1241:1329&appname=ncbiblast&link_loc=fromHSP)Next MatchPrevious Match[First Match](https://blast.ncbi.nlm.nih.gov/Blast.cgi#hspQuery_89088_1)

| Alignment statistics for match #8 | | | | |
| --- | --- | --- | --- | --- |
| **Score** | **Expect** | **Identities** | **Gaps** | **Strand** |
| 123 bits(136) | 5e-30 | 76/81(94%) | 0/81(0%) | Plus/Plus |

Query 2583 AGGGATATCAAAGGGGCAAACATACTTGTAGATCCTAATGGTGACATCAAGCTTGCTGAT 2642

||||||||||||||||||||||||||||| ||||||||||||||||||||||||||||||

Sbjct 1245 AGGGATATCAAAGGGGCAAACATACTTGTCGATCCTAATGGTGACATCAAGCTTGCTGAT 1304

Query 2643 TTTGGTATGGCGAAACATGTA 2663

||||| ||||| || ||| ||

Sbjct 1305 TTTGGCATGGCCAAGCATATA 1325

Range 9: 1740 to 1856[Graphics](https://www.ncbi.nlm.nih.gov/projects/sviewer/?RID=SUYPNXCX114&id=lcl|Query_89088&tracks=%5bkey:sequence_track,name:Sequence,display_name:Sequence,id:STD1,category:Sequence,annots:Sequence,ShowLabel:true%5d%5bkey:gene_model_track,CDSProductFeats:false%5d%5bkey:alignment_track,name:other%20alignments,annots:NG%20Alignments|Refseq%20Alignments|Gnomon%20Alignments|Unnamed,shown:false%5d&v=1735:1861&appname=ncbiblast&link_loc=fromHSP)Next MatchPrevious Match[First Match](https://blast.ncbi.nlm.nih.gov/Blast.cgi#hspQuery_89088_1)

| Alignment statistics for match #9 | | | | |
| --- | --- | --- | --- | --- |
| **Score** | **Expect** | **Identities** | **Gaps** | **Strand** |
| 122 bits(134) | 2e-29 | 97/117(83%) | 0/117(0%) | Plus/Plus |

Query 3594 ATTGAAACTTCGTCATACAGAAGTTTATCTCCTTTAAGAGATCCTGATATCCTTGGAAGA 3653

|||| | ||| ||||||||||| |||||||| ||||||||||||||| || | ||||

Sbjct 1740 ATTGCAGTTTCATCATACAGAAGCTTATCTCCATTAAGAGATCCTGATGTCGTGATAAGA 1799

Query 3654 AACTTGCCAGGACCAACATCCCCTATTCCTTCGACATCAAGTCGCAGGATCGCAGCA 3710

|| |||| ||||||||||||||| |||||| ||| |||| ||||||||| || |||

Sbjct 1800 AATTTGCAAGGACCAACATCCCCCATTCCTCCGATGTCAAATCGCAGGATTGCGGCA 1856

Range 10: 1020 to 1085[Graphics](https://www.ncbi.nlm.nih.gov/projects/sviewer/?RID=SUYPNXCX114&id=lcl|Query_89088&tracks=%5bkey:sequence_track,name:Sequence,display_name:Sequence,id:STD1,category:Sequence,annots:Sequence,ShowLabel:true%5d%5bkey:gene_model_track,CDSProductFeats:false%5d%5bkey:alignment_track,name:other%20alignments,annots:NG%20Alignments|Refseq%20Alignments|Gnomon%20Alignments|Unnamed,shown:false%5d&v=1017:1088&appname=ncbiblast&link_loc=fromHSP)Next MatchPrevious Match[First Match](https://blast.ncbi.nlm.nih.gov/Blast.cgi#hspQuery_89088_1)

| Alignment statistics for match #10 | | | | |
| --- | --- | --- | --- | --- |
| **Score** | **Expect** | **Identities** | **Gaps** | **Strand** |
| 105 bits(116) | 1e-24 | 63/66(95%) | 0/66(0%) | Plus/Plus |

Query 2103 CAGGAAATCGTGCTGCTGAGTCAGCTGTCACATCCAAACATTGTTCAGTACTATGGCAGT 2162

||||||||| |||||||||||||||||||||||||||||||||| |||||||||||||||

Sbjct 1020 CAGGAAATCATGCTGCTGAGTCAGCTGTCACATCCAAACATTGTACAGTACTATGGCAGT 1079

Query 2163 GATTTG 2168

||| ||

Sbjct 1080 GATCTG 1085

Range 11: 1320 to 1384[Graphics](https://www.ncbi.nlm.nih.gov/projects/sviewer/?RID=SUYPNXCX114&id=lcl|Query_89088&tracks=%5bkey:sequence_track,name:Sequence,display_name:Sequence,id:STD1,category:Sequence,annots:Sequence,ShowLabel:true%5d%5bkey:gene_model_track,CDSProductFeats:false%5d%5bkey:alignment_track,name:other%20alignments,annots:NG%20Alignments|Refseq%20Alignments|Gnomon%20Alignments|Unnamed,shown:false%5d&v=1317:1387&appname=ncbiblast&link_loc=fromHSP)Next MatchPrevious Match[First Match](https://blast.ncbi.nlm.nih.gov/Blast.cgi#hspQuery_89088_1)

| Alignment statistics for match #11 | | | | |
| --- | --- | --- | --- | --- |
| **Score** | **Expect** | **Identities** | **Gaps** | **Strand** |
| 104 bits(114) | 5e-24 | 62/65(95%) | 0/65(0%) | Plus/Plus |

Query 2757 CAGATATCAGCATATACATCTATCAAATCCTTCAAAGGGAGCCCTTACTGGATGGCACCA 2816

|| ||||||||||| |||||||||| ||||||||||||||||||||||||||||||||||

Sbjct 1320 CATATATCAGCATACACATCTATCAGATCCTTCAAAGGGAGCCCTTACTGGATGGCACCA 1379

Query 2817 GAGGT 2821

|||||

Sbjct 1380 GAGGT 1384

Query: GGRMZM6G513881.

Subject: isotig04689 gene=isogroup00742

| Alignment statistics for match #1 | | | | |
| --- | --- | --- | --- | --- |
| **Score** | **Expect** | **Identities** | **Gaps** | **Strand** |
| 75.2 bits(82) | 2e-15 | 64/79(81%) | 0/79(0%) | Plus/Plus |

Query 2585 GGATATCAAAGGGGCAAACATACTTGTAGATCCTAATGGTGACATCAAGCTTGCTGATTT 2644

|||||| | ||| |||||||||||||| | ||||||||| ||||| |||||||| ||

Sbjct 5721 GGATATAATAGGAGCAAACATACTTGTGGGCAGTAATGGTGAAATCAAACTTGCTGACTT 5780

Query 2645 TGGTATGGCGAAACATGTA 2663

||| ||||| || | ||||

Sbjct 5781 TGGCATGGCTAAGCTTGTA 5799

Range 2: 5557 to 5696[Graphics](https://www.ncbi.nlm.nih.gov/projects/sviewer/?RID=SUYPNXCX114&id=lcl|Query_89091&tracks=%5bkey:sequence_track,name:Sequence,display_name:Sequence,id:STD1,category:Sequence,annots:Sequence,ShowLabel:true%5d%5bkey:gene_model_track,CDSProductFeats:false%5d%5bkey:alignment_track,name:other%20alignments,annots:NG%20Alignments|Refseq%20Alignments|Gnomon%20Alignments|Unnamed,shown:false%5d&v=5551:5702&appname=ncbiblast&link_loc=fromHSP)Next MatchPrevious Match[First Match](https://blast.ncbi.nlm.nih.gov/Blast.cgi#hspQuery_89091_1)

| Alignment statistics for match #2 | | | | |
| --- | --- | --- | --- | --- |
| **Score** | **Expect** | **Identities** | **Gaps** | **Strand** |
| 71.6 bits(78) | 3e-14 | 102/141(72%) | 2/141(1%) | Plus/Plus |

Query 2319 ACTCTCGGTCTATCTCGAGTATGTTTCTGGGGGCTCTATCCATAAGTTGCTTCAAGAA-T 2377

||| || |||| | || |||||||||||| || ||||||| ||| ||| | ||| |

Sbjct 5557 ACTTTCAATCTACATGGAATATGTTTCTGGGAATTCAATCCATAGGTTACTT-ATGAAGT 5615

Query 2378 ATGGTCCGTTTGGGGAGGCAGTGCTTCGGAATTACACAGCACAAATCCTTTCTGGCCTTG 2437

|||| || | ||| | | |||| |||||||| |||||| || ||||||||

Sbjct 5616 ATGGCCCTCGAGAGGAATCTACGATTCGCAATTACACTAAACAAATTCTCCTTGGCCTTG 5675

Query 2438 CATACTTGCATGGGCGGAATA 2458

| |||||||||||| |||||

Sbjct 5676 CTTACTTGCATGGGGAGAATA 5696

Range 3: 6311 to 6387[Graphics](https://www.ncbi.nlm.nih.gov/projects/sviewer/?RID=SUYPNXCX114&id=lcl|Query_89091&tracks=%5bkey:sequence_track,name:Sequence,display_name:Sequence,id:STD1,category:Sequence,annots:Sequence,ShowLabel:true%5d%5bkey:gene_model_track,CDSProductFeats:false%5d%5bkey:alignment_track,name:other%20alignments,annots:NG%20Alignments|Refseq%20Alignments|Gnomon%20Alignments|Unnamed,shown:false%5d&v=6308:6390&appname=ncbiblast&link_loc=fromHSP)Next MatchPrevious Match[First Match](https://blast.ncbi.nlm.nih.gov/Blast.cgi#hspQuery_89091_1)

| Alignment statistics for match #3 | | | | |
| --- | --- | --- | --- | --- |
| **Score** | **Expect** | **Identities** | **Gaps** | **Strand** |
| 50.0 bits(54) | 9e-08 | 57/77(74%) | 0/77(0%) | Plus/Plus |

Query 2928 ATGGTTACAGCCTTTCAGTAGACATTTGGAGCCTTGGCTGCACCATTCTTGAGATGGCAA 2987

||||||||| || || |||||||||||| | || || || || |||||| ||| |

Sbjct 6311 ATGGTTACAATCTACAGGTGGACATTTGGAGCTTAGGATGTACTGTTATTGAGACGGCGA 6370

Query 2988 CAGCAAAGCCTCCTTGG 3004

| | ||| | |||||||

Sbjct 6371 CGGGAAAACATCCTTGG 6387

Range 4: 6255 to 6287[Graphics](https://www.ncbi.nlm.nih.gov/projects/sviewer/?RID=SUYPNXCX114&id=lcl|Query_89091&tracks=%5bkey:sequence_track,name:Sequence,display_name:Sequence,id:STD1,category:Sequence,annots:Sequence,ShowLabel:true%5d%5bkey:gene_model_track,CDSProductFeats:false%5d%5bkey:alignment_track,name:other%20alignments,annots:NG%20Alignments|Refseq%20Alignments|Gnomon%20Alignments|Unnamed,shown:false%5d&v=6254:6288&appname=ncbiblast&link_loc=fromHSP)Next MatchPrevious Match[First Match](https://blast.ncbi.nlm.nih.gov/Blast.cgi#hspQuery_89091_1)

| Alignment statistics for match #4 | | | | |
| --- | --- | --- | --- | --- |
| **Score** | **Expect** | **Identities** | **Gaps** | **Strand** |
| 33.7 bits(36) | 0.007 | 27/33(82%) | 0/33(0%) | Plus/Plus |

Query 2789 CAAAGGGAGCCCTTACTGGATGGCACCAGAGGT 2821

|||||| | || ||||||||||| || |||||

Sbjct 6255 CAAAGGAACTCCATACTGGATGGCTCCTGAGGT 6287

**Supplementary Fig. S8. Alignments of maize sequence GGRMZM6G513881 (NC_024462.2:15133270-15137604 *Zea mays* cultivar B73 chromosome 4) with *PN_QGJ* and *PN_LNC_QGJ* isotigs.** Top: scheme of the BLAST alignments. Bottom: alignments between query GGRMZM6G513881 and each one of the *PN_ QG*J/*PN_LNC_QGJ* isotigs.
